# Supplementary figures and images for: Canine urothelial cell model to study intracellular bacterial community development by uropathogenic Escherichia coli
Source: PLoS One. 2025 Jan 9;20(1):e0316834. doi: 10.1371/journal.pone.0316834 (PMC11717241; doi:10.1371/journal.pone.0316834)

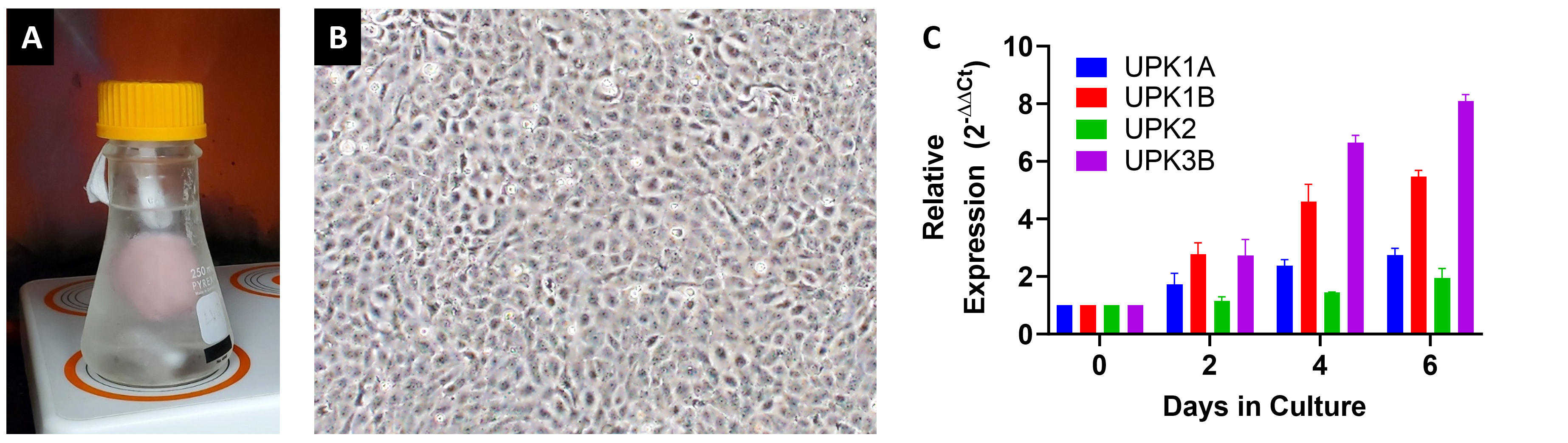

Supplement: S1 Fig — Canine bladders (n = 10) were aseptically collected postmortem from apparently healthy dogs after planned euthanasia by an animal control facility. (A) The bladder was removed, turned inside out to expose the mucosa and suspended in an Erlenmeyer flask under constant rotation. The tissue was treated with Dispase to remove the urothelial cells and cells were subsequently seeded onto collagen coated flasks and grown in culture. (B) By 7 days a pure urothelial cell culture was appreciated by brightfield microscopy (10x) and analysis of cellular morphology as described by others [40,84,85,88]. Urothelial cells were frozen in aliquots and stored in liquid nitrogen until future use. (C) Canine urothelial cells were thawed, and seeded collagen coated tissue culture plates and grown until confluency. Thereafter, the culture media was changed to promote urothelial cell differentiation for expression of uroplakins required for UPEC pathogenesis as described elsewhere[41,86,89]. Expression of various uroplakins was determined by real-time polymerase chain reaction using canine specific primers and the housekeeping gene GAPDH. Relative expression compared to the day 0 (before changing to UPDM) is presented at 2-ΔΔCt. (TIF) [file pone.0316834.s001.tif]

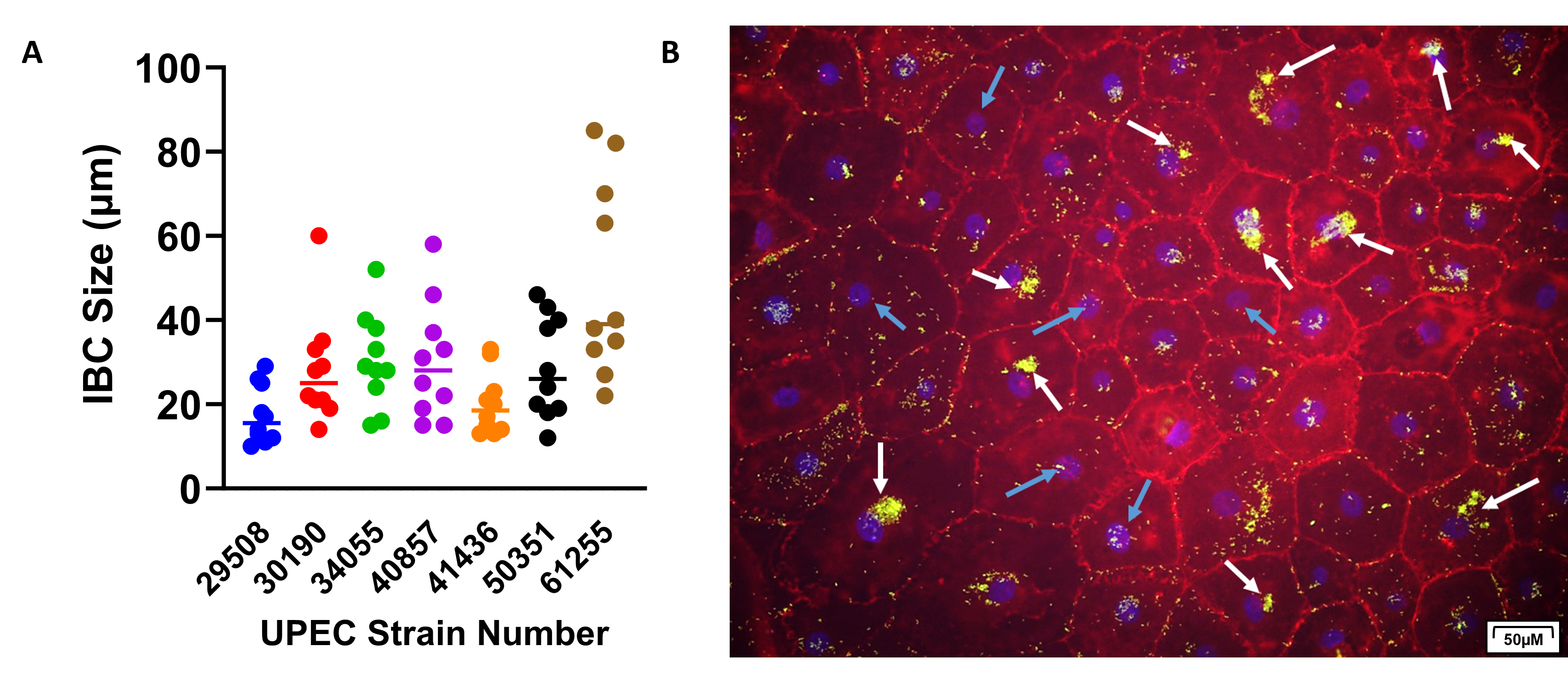

Supplement: S2 Fig — Intracellular bacterial communities were evaluated via immunofluorescence microscopy. (A) Canine urothelial cells were infected with canine specific UPECs designated by a strain number. Ten IBCs per strain were measured. The minimum size of the communities was observed as 10uM with the maximum size being 85uM with an average of 29μm. The average of the length and width was taken to denote size as these images were taken in two dimensions not three. (B) Examples of cells with IBCs (white arrows) that were counted and examples of cells with intracellular bacteria but not IBCs that were not counted (blue arrows). (TIF) [file pone.0316834.s002.tif]

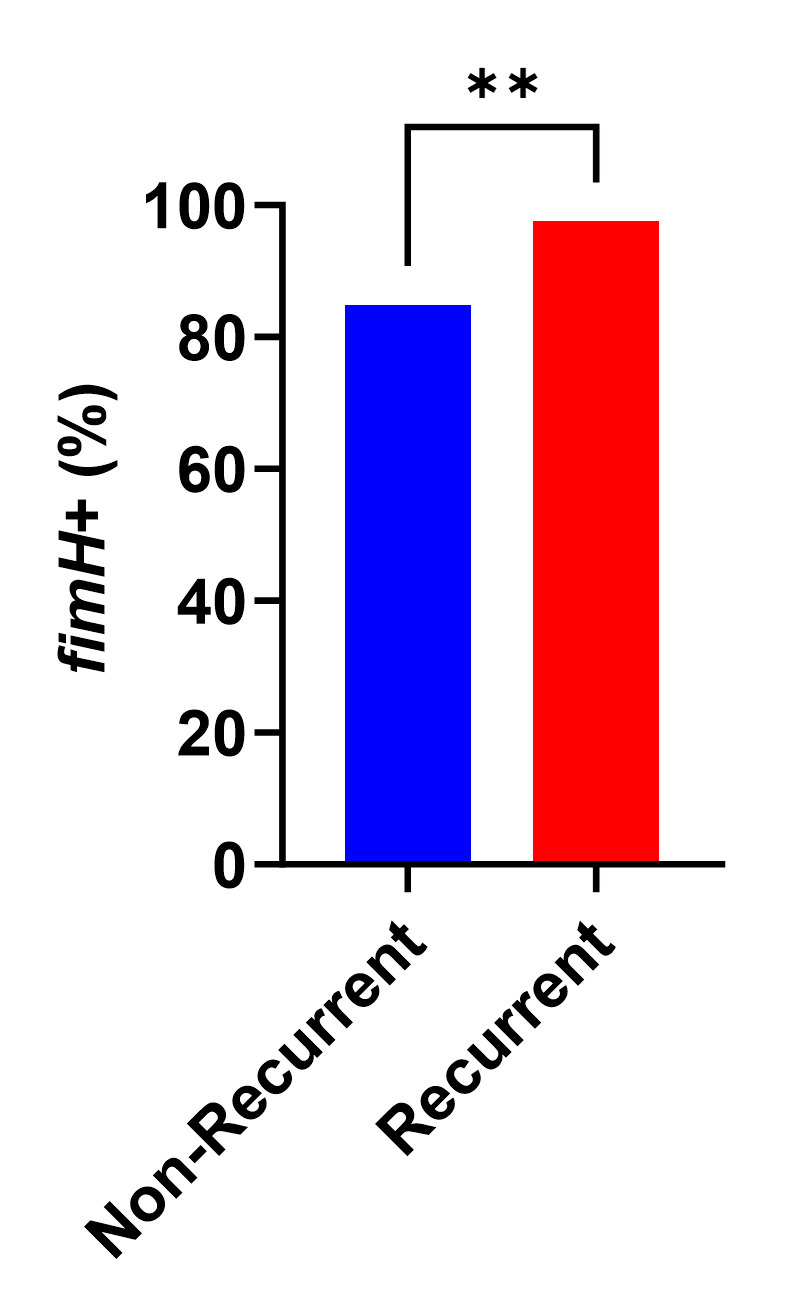

Supplement: S3 Fig — Canine UPEC isolates (n = -206) were classified as recurrent with well-documented recurrence of the same phenotypic strain within 6 months of the initial UTI. (A) Each isolate was evaluated for the presence or absence of the fimH gene by PCR. (B) Incidence of multidrug resistance was defined by resistance to greater than or equal to three antimicrobial drug classes. Bars are means and standard deviations and significant differences (p<0.05) were determined by an unpaired, nonparametric t-test. Statistically significant differences are denoted with asterisks as follows: *p<0.05, **p<0.01, ***p<0.001, and ****p<0.0001. (TIF) [file pone.0316834.s003.tif]
