# Supplementary material for: Canine urothelial cell model to study intracellular bacterial community development by uropathogenic Escherichia coli
Source: PLoS One. 2025 Jan 9;20(1):e0316834. doi: 10.1371/journal.pone.0316834 (PMC11717241; doi:10.1371/journal.pone.0316834)
Supplement: Attachment — Submitted filename: PONE-D-22-30262_R1_reviewer_.pdf [file pone.0316834.s005.pdf]

PLOS ONE

Canine urothelial cell model to study intracellular bacterial community development by uropathogenic Escherichia coli

--Manuscript Draft--

**Manuscript Number:**	PONE-D-22-30262R1
**Article Type:**	Research Article
**Full Title:**	Canine urothelial cell model to study intracellular bacterial community development by uropathogenic Escherichia coli
**Short Title:**	Canine urothelial cells support intracellular Escherichia coli
**Corresponding Author:**	Megan Jacob<br>North Carolina State University<br>Raleigh, UNITED STATES
**Keywords:**	Escherichia coli, uropathogen, urinary tract infection, animal model, canine, dog, biofilms
**Abstract:**	Urinary tract infections (UTIs) are among the most common bacterial infections of both dogs and humans, with most caused by uropathogenic Escherichia coli (UPEC). Recurrent UPEC infections are a major concern in the treatment and management of UTIs in both species. In humans, the ability of UPECs to form intracellular bacterial communities (IBCs) within urothelial cells has been implicated in recurrent UTIs. However, the role of IBCs has not been explored in the pathogenesis of canine recurrent UTIs. In this study, we identified IBCs in both urine and bladder tissue from dogs with UPEC associated UTIs. In addition, we showed that UPECs derived from canine UTIs form IBCs within primary canine urothelial cells. As in human UTIs, formation of IBCs by canine UPECs correlated with the presence of the fimH gene as those isolates lacking the fimH gene formed fewer IBCs in canine urothelial cells then those carrying the fimH gene. Additionally, UPEC strains from clinical cases classified as recurrent UTIs had higher rates of IBC formation than UPEC strains from non-recurrent UTIs. These IBCs were tolerant to treatment with enrofloxacin, cefpodoxime and doxycycline at 150, 50 and 50 µg/mL respectively, which are representative of the concentrations achieved in canine urine after standard dosing. This is consistent with the clinical perspective that current UTIs are a common condition of dogs and are difficult to manage through antimicrobial treatment. Additionally, the dog could prove to be a powerful model of IBC formation as they are natural models of UPEC-causing UTIs and have similar pathophysiology of IBC formation.
**Order of Authors:**	Jessica Gilbertie
	Megan Jacob
	Breanna Sheahan
	Shelly Vaden
**Response to Reviewers:**	We thank the reviewers for their time and thoughtful suggestions on improving this manuscript. There are detailed responses to each concern noted.<br><br>Reviewer #1: In this paper, Gilbertie and colleagues describe a series of experiments identifying intracellular uropathogenic E. coli (UPEC) in the urothelial tissues of dogs with UTIs, as well as the capability of canine uroepithelial cells to be propagated and to support an intracellular, IBC-like state for UPEC. Several suggestions for improvement are given below.<br><br>Major points:<br>1. The Introduction could do a better job of specifying what is and what is not known about canine UTI prior to the present study. In the passage starting on line 69, the authors state that “dogs have similar UTI pathogenesis” to humans and that dogs have similar rates of drug-resistant uropathogens, giving multiple references for these statements. Then, in the subsequent paragraph, the authors state that the goal of the present study was to “determine if dogs demonstrate similar UTI pathogenesis” to humans, and that the present paper “demonstrated that dogs are clinically affected by

similar uropathogens with similar resistance rates…" On a related note, the authors state (line 70) that dogs have "urinary biology comparable to humans" but this statement needs more specifics. What aspects of urinary biology are similar between the two species?

We agree that the introduction was confusing, and we appreciate the reviewers point. We have edited the introduction to state that we know dogs have similar UTI epidemiology in that they are infected with similar etiologic agents, are infected by similar UPEC sequence types, have similar MDR and ESBL carriage within their UPEC population and are afflicted by similar recurrence rates. However, if canine and human UTIs have a similar pathogenesis is unknown. Therefore, the goal of this manuscript was to determine if IBCs form in canine UTIs in a similar manner as observed in humans.

2. The letter-labels for the panels in Figure 1 don't match between the legend (lines 116-128) and the figure itself.

Thank you for noting this mistake. This has now been corrected.

3. In the paragraph starting on line 250, it appears that 12 recurrent UTI strains and 12 non-recurrent strains were evaluated. But then "97.5%" and "84.9%" of the strains carry fimH, which presumably reflects n=206 (Figure S2 legend)? The text in this paragraph should be clarified as to how many strains were evaluated for each analysis.

We edited this experimental description for clarity.

4. In Figure 7, it isn't clear what is meant by "significant differences are indicated by differing letters" – in other words, what the a's and b's mean in panels A and B.

Letter designations are given when using a Tukey post hoc for multiple comparisons. Therefore, a bar with an "a" above it is statistically different from a bar with any other letter designations such as "b" or "c" but it not different from another "a" or a bar with an "a,b" above it. We tried to clarify that in the figure legend more.

Accompanying this, it would be helpful if in the Discussion, the authors commented on these differences and how they might correlate with the likely intracellular penetration of the chosen antibiotics. Specifically, fluoroquinolones (e.g., enrofloxacin) and doxycycline would be expected to penetrate cells better than a beta-lactam (here cefpodoxime), and there appears to be a correlation between this property and the antibacterial effect as measured by CFU and IBC numbers.

Good point! We have added this observation to the discussion section; in the interest of discussion length as previously noted by reviewers, we did not go into detail as it was not the primary outcome of interest; we can certainly develop this further if the reviewer feels like it would be important.

5. In Figure S2, the measurements of both fimH carriage (panel A) and MDR (panel B) are binary in nature. So, what do the bars represent here? There should not be standard deviations in this type of data. Accompanying that, one would expect that a chi-square analysis rather than "an unpaired, nonparametric t-test" (line 279) would be appropriate for comparisons.

We removed the error bars and performed a chi-square test instead of a t-test. The figure has been updated to represent the edits.

6. The vertebrate animals information would benefit from additional clarification. The authors state that all the samples were collected for clinical purposes and prior to the start of any analysis (i.e., they were banked). What was the clinical indication for bladder biopsies in dogs with active UTIs?

Bladder biopsies are taken during uroscopy at the discretion of the treating veterinarian. The biopsies are sent to the pathology service to evaluate the bladder tissue for the presence of inflammation, infection, or neoplasm. These samples are banked and can be used for research purposes. We have tried to clarify this in the

Powered by Editorial Manager® and ProduXion Manager® from Aries Systems Corporation

	manuscript by adding that these are diagnostic specimens submitted for the management of a patient and not because of inclusion in the study. Still, all samples submitted to the NCSU CVM are approved to be used for research purposes.<br><br>Minor points:<br>1. Given that no methodologic details are not fully available in the abstract, one should remove the specific numerical data from the abstract. For example, “4.2 log10 CFU/well” doesn’t have specific meaning as the reader hasn’t yet read the methods.<br><br>This numerical data has been removed from the abstract per this reviewer’s request.<br><br>2. Line 44: “20% of dogs with UTIs will develop a recurrent infection…” during what period of follow-up (in reference 4)?<br><br>This has been updated.<br><br>3. Line 182: Beyond “similar morphology,” can the authors be more specific as to the size of intracellular communities or the apparent number of bacteria, in canine uroepithelial cells vs the immortalized human bladder cell line?<br><br>Not at this stage but this would be a good idea for future work.<br><br>4. Some of the figure legends could be shortened by reducing methodologic detail (these details already appear in the Methods section).<br><br>We have followed this suggestion and reduced the figure legends.<br><br>5. The Discussion could probably also be shortened by reducing the degree to which the current Results are recapped.<br><br>We have followed this advice and removed paragraph 1 that recapped the results.<br><br>6. Panels A and B of Figure 2 could probably be eliminated, as they reflect simple percentages that are described in the text.<br><br>We prefer to leave panels A and B in Figure 2 in but will leave this to the discretion of the editor.<br><br>Reviewer #2: The authors have written an informative manuscript. However, the authors should proofread the manuscript and correct some minor spelling mistakes and strictly follow the journal's author guidelines.<br><br>The sentence in line 22 and 23 is incomplete. Corrected.<br><br>The sentence in line 63 and 64 is not well understood. We have edited this sentence.<br><br>Please check the spelling of ' their) in line 66. Corrected.<br><br>Please elaborate more on the statistical analysis. Most of the statement have been made in the 'results' section. For example, non-parametric t-test. We have added to the statistical analysis section with statements from our figures/results sections.<br><br>The authors did not include control strains in the experimental section. We did not use any control strains only clinical UPEC strains from canine UTIs.<br><br>Authors should please take a second look at the clarity of the labels. We have double checked the labels and corrected where appropriate. Most of this clarification was addressed through other reviewer comments.<br><br>Authors should make comments on the ethical approval. We have added further comments on the ethical approval.
**Additional Information:**	
**Question**	**Response**

Powered by Editorial Manager® and ProduXion Manager® from Aries Systems Corporation

**Financial Disclosure**<br><br>Enter a financial disclosure statement that describes the sources of funding for the work included in this submission. Review the submission guidelines for detailed requirements. View published research articles from *PLOS ONE* for specific examples.<br><br>This statement is required for submission and **will appear in the published article** if the submission is accepted. Please make sure it is accurate.<br><br>**Unfunded studies**<br>Enter: *The author(s) received no specific funding for this work.*<br><br>**Funded studies**<br>Enter a statement with the following details:<br>• Initials of the authors who received each award<br>• Grant numbers awarded to each author<br>• The full name of each funder<br>• URL of each funder website<br>• Did the sponsors or funders play any role in the study design, data collection and analysis, decision to publish, or preparation of the manuscript?<br>• **NO** - Include this sentence at the end of your statement: *The funders had no role in study design, data collection and analysis, decision to publish, or preparation of the manuscript.*<br>• **YES** - Specify the role(s) played.<br><br>* typeset	The author(s) received no specific funding for this work.
**Competing Interests**<br><br>Use the instructions below to enter a competing interest statement for this submission. On behalf of all authors, disclose any competing interests that could be perceived to bias this work—acknowledging all financial support and any other relevant financial or non-financial competing interests.<br><br>This statement is required for submission	The authors have declared that no competing interests exist.

and **will appear in the published article** if the submission is accepted. Please make sure it is accurate and that any funding sources listed in your Funding Information later in the submission form are also declared in your Financial Disclosure statement.<br><br>View published research articles from *PLOS ONE* for specific examples.<br><br>**NO authors have competing interests**<br><br>Enter: *The authors have declared that no competing interests exist.*<br><br>**Authors with competing interests**<br><br>Enter competing interest details beginning with this statement:<br><br>*I have read the journal's policy and the authors of this manuscript have the following competing interests: [insert competing interests here]*<br><br>* typeset	
**Ethics Statement**<br><br>Enter an ethics statement for this submission. This statement is required if the study involved:<br><br>• Human participants<br>• Human specimens or tissue<br>• Vertebrate animals or cephalopods<br>• Vertebrate embryos or tissues<br>• Field research<br><br>Write "N/A" if the submission does not require an ethics statement.<br><br>General guidance is provided below. Consult the submission guidelines for detailed instructions. **Make sure that all information entered here is included in the Methods section of the manuscript.**	All diagnostic samples used in this study were acquired retrospectively; the decision to submit samples was at the discretion of the treating clinician. Canine urothelial cells were obtained from dogs humanely euthanized at a local animal control facility for reasons unrelated to the study and at the discretion of the facility; IACUC approval was not required.

Powered by Editorial Manager® and ProduXion Manager® from Aries Systems Corporation

**Format for specific study types**<br><br>**Human Subject Research (involving human participants and/or tissue)**<br>• Give the name of the institutional review board or ethics committee that approved the study<br>• Include the approval number and/or a statement indicating approval of this research<br>• Indicate the form of consent obtained (written/oral) or the reason that consent was not obtained (e.g. the data were analyzed anonymously)<br><br>**Animal Research (involving vertebrate animals, embryos or tissues)**<br>• Provide the name of the Institutional Animal Care and Use Committee (IACUC) or other relevant ethics board that reviewed the study protocol, and indicate whether they approved this research or granted a formal waiver of ethical approval<br>• Include an approval number if one was obtained<br>• If the study involved *non-human primates*, add *additional details* about animal welfare and steps taken to ameliorate suffering<br>• If anesthesia, euthanasia, or any kind of animal sacrifice is part of the study, include briefly which substances and/or methods were applied<br><br>**Field Research**<br><br>Include the following details if this study involves the collection of plant, animal, or other materials from a natural setting:<br>• Field permit number<br>• Name of the institution or relevant body that granted permission	
**Data Availability**<br><br>Authors are required to make all data underlying the findings described fully available, without restriction, and from the time of publication. PLOS allows rare exceptions to address legal and ethical concerns. See the PLOS Data Policy and FAQ for detailed information.	Yes - all data are fully available without restriction

A Data Availability Statement describing where the data can be found is required at submission. Your answers to this question constitute the Data Availability Statement and **will be published in the article**, if accepted.<br><br>**Important:** Stating 'data available on request from the author' is not sufficient. If your data are only available upon request, select 'No' for the first question and explain your exceptional situation in the text box.<br><br>Do the authors confirm that all data underlying the findings described in their manuscript are fully available without restriction?	
**Describe where the data may be found in full sentences. If you are copying our sample text, replace any instances of XXX with the appropriate details.**<br><br>• If the data are **held or will be held in a public repository**, include URLs, accession numbers or DOIs. If this information will only be available after acceptance, indicate this by ticking the box below. For example: *All XXX files are available from the XXX database (accession number(s) XXX, XXX.)*.<br>• If the data are all contained **within the manuscript and/or Supporting Information files**, enter the following: *All relevant data are within the manuscript and its Supporting Information files.*<br>• If neither of these applies but you are able to provide **details of access elsewhere**, with or without limitations, please do so. For example:<br><br>*Data cannot be shared publicly because of [XXX]. Data are available from the XXX Institutional Data Access / Ethics Committee (contact via XXX) for researchers who meet the criteria for access to confidential data.*<br><br>*The data underlying the results presented in the study are available from (include the name of the third party*	All relevant data are within the manuscript and its supplementary figures.

Powered by Editorial Manager® and ProduXion Manager® from Aries Systems Corporation

and contact information or URL).<br>• This text is appropriate if the data are owned by a third party and authors do not have permission to share the data.<br><br>* typeset	
Additional data availability information:	

Powered by Editorial Manager® and ProduXion Manager® from Aries Systems Corporation

Click here to access/download;Manuscript;Gilbertie Canine UPEC Model Manuscript FINAL methods no tc.docx

Canine urothelial cell model to study intracellular bacterial community development by uropathogenic *Escherichia coli*

Canine urothelial cells support intracellular *Escherichia coli*

Jessica M. Gilbertie[1,2], Breanna J. Sheahan[2,3], Shelly L. Vaden[3], Megan E. Jacob[1,2 *]

[1] Department of Population Health and Pathobiology, College of Veterinary Medicine, North Carolina State University, Raleigh, North Carolina, USA

[2]Comparative Medicine Institute, North Carolina State University, Raleigh, NC, USA

[3]Department of Clinical Sciences, College of Veterinary Medicine, North Carolina State University, Raleigh, North Carolina, USA

***Corresponding author:**

Email: mejacob@ncsu.edu

Abstract

Urinary tract infections (UTIs) are the most common bacterial infection of both dogs and humans, with most caused by uropathogenic *Escherichia coli* (UPEC). Recurrent UPEC infections are a major concern in the treatment and management of UTIs in both dogs and humans. In humans, the ability of UPECs to form intracellular bacterial communities (IBCs) within urothelial cells has been implicated in recurrent UTIs. However, the role of IBCs has not been explored in the pathogenesis of canine recurrent UTIs. In this study, we identified IBCs in both urine and bladder tissue from dogs with UPEC associated. In addition, we showed that

UPECs derived from canine UTIs form IBCs within primary canine urothelial cells (5.8 IBCs/HPF and 4.3 $\log_{10}$CFU/well). As in human UTIs, formation of IBCs by canine UPECs correlated with the presence of the *fimH* gene as those isolates lacking the *fimH* gene formed fewer IBCs in canine urothelial cells (0.5 IBCs/HPF and 0.8 $\log_{10}$CFU/well) then those harboring the *fimH* gene (7.7 IBCs/HPF and 5.1 $\log_{10}$CFU/well). Additionally, UPEC strains from clinical cases classified as recurrent UTIs had higher rates of IBC formation (12.8 IBCs/HPF and 7.6 $\log_{10}$CFU/well) than UPEC strains from non-recurrent UTIs (5.5 IBCs/HPF and 4.2 $\log_{10}$CFU/well). These IBCs were tolerant to treatment with enrofloxacin, cefpodoxime and doxycycline at 150, 50 and 50 µg/mL respectively, which are representative of the concentrations achieved in canine urine after standard dosing. This is consistent with the clinical perspective that current UTIs are a common condition of dogs and are difficult to manage through antimicrobial treatment. Additionally, the dog could prove to be a powerful model of IBC formation as they are natural models of UPEC-causing UTIs and have similar pathophysiology of IBC formation.

Introduction

Urinary tract infections (UTI) are the most common bacterial infection of dogs accounting for 14% of medical cases. Uropathogenic *Escherichia coli* (UPEC) are the most common etiological agents in both human and canine UTIs[1,2]. Recurrent infections are a major concern in the treatment and management of UTIs[3]. Previous reports show that 20% of dogs with UTIs will develop a recurrent infection despite aggressive antimicrobial therapy[4]. In humans, recurrent UTIs are associated with the ability of UPEC to form intracellular bacterial communities (IBCs)

that are tolerant to antimicrobial therapy[5–8]; however, the role of IBCs in canine UTIs has not been demonstrated.

There has been an increasing interest in the relationship between UPEC biofilm potential and infection persistence in recurrent UTIs; however, a previous study from our group was unable to identify associations between traditional biofilm formation *in vitro* and clinical recurrence of UTI in dogs[9]. The traditional definition of a biofilm is a community of bacteria within a polymeric matrix that is attached to an abiotic or biotic surface[10]. However, recent advancements in biofilm research suggest that, in the urinary tract, IBCs are considered non-surface attached, intracellular biofilms [11].

In human isolates, the ability of UPEC to adhere to and invade the urothelium to form IBCs is facilitated by the type 1 pilus-associated adhesin FimH[12]. The majority of UPECs encode the *fimH* gene, a part of the *fim* operon, which is transcribed to the FimH adhesion protein[13]. FimH binds to mannose-containing glycoprotein receptors on host cell membranes, which in the urinary tract, are the mannosylated residues on uroplakin proteins that line the apical surface of urothelial cells in the bladder[14]. UPEC utilize binding of FimH to the uroplakin receptor complex to enable adhesion to the urothelial surface[12]. Subsequently, the binding of FimH to uroplakin triggers host cell signaling promoting bacterial internalization[15]. Thereafter, UPEC grow and divide intracellularly forming small clusters of bacteria termed IBCs[16]. Once an IBC develops UPEC are protected, able to tolerate the -cidal activity of antimicrobials[6,17].

Dogs are an ideal translational model for urinary tract infection research. Their size allows for repeated access to large volumes of urine using minimally invasive techniques[18]. Additionally, biopsies of bladder tissue can be easily accomplished via uroscopy. Dogs are commonly used as models of human disease and are great pharmacokinetic models for novel

therapeutics[19–21]. In addition, dogs have similar urinary tract infection pathogenesis, are afflicted by the same types of bacteria, and have urinary biology comparable to humans[2,4,22,23]. For UPEC specific UTIs, dogs are infected by similar sequence types (STs), have similar rates of multi-drug resistant strains, and canine *E. coli* isolates have shown similar virulence traits and biofilm phenotypes as human isolates [24–26].

The goal of this study was to determine if dogs demonstrate similar UPEC associated UTI pathogenesis predominated by IBC formation as observed in humans and murine models[27]. Through retrospective data analysis, we demonstrated that dogs are clinically afflicted by similar uropathogens with similar resistance rates and recurrent infection frequencies as human UTIs. In clinical specimens, we demonstrated that UPEC-causing UTIs were associated with IBCs on both urine cytologies and bladder biopsies in symptomatic dogs; additionally, we validated an *in vitro* IBC model using primary canine urothelial cells. Using this *in vitro* IBC model, we validated the role of *fimH* in IBC formation in urothelial cells, assessed IBCs tolerance to treatment with several classes of clinically relevant antimicrobials, and compared IBCs *in vitro* from UPEC isolated from recurrent UTI cases compared to UPEC isolated from nonrecurrent UTI cases.

Results

Canine UTIs exhibit similar uropathogen distribution, antimicrobial resistance and recurrent infection frequencies as human UTIs

To compare the epidemiology of canine and human UTIs we performed a retrospective analysis of canine uropathogens collected at a veterinary teaching hospital over a 5-year period (2015-2019). During that time, 797 canine urine isolates were obtained, of which 87.3% were

monospecies cultures and 12.7% were polymicrobial. The most common UTI associated pathogen was *Escherichia coli* (39.1% of positive cultures), followed by *Enterococcus* spp. (15.7%), *Streptococcus* spp. (10.7%), *Staphylococcus pseudintermedius* (8%), *Klebsiella pneumoniae* (5.6%), and *Proteus mirabilis* (5.6%) (Fig 1A). In humans, *E. coli* is the most common pathogen causing UTIs as well; however, occurrence is higher with reported rates from 65-75%[28,29]. The second most common UTI associated pathogen of humans varies by study and are similar to canine UTI associated pathogens including *Staphylococcus saprophyticus*, *K. pneumoniae*, *P. mirabilis* and *Enterococcus* spp.[30]. Of the polymicrobial infections in our analysis, the most commonly isolated combination was *Escherichia coli* and *Enterococcus* spp. (typically *faecium* and *faecalis*) (24.5%). The *E. coli*-*Enterococcus* spp. combination is also seen in approximately 36% of human polymicrobial UTIs[31]. The sex distribution was 60.9% female (female intact and female spayed) and 39.1% male (male intact and male castrated) and the median age at presentation was 5.6 years (middle aged adult). Similarly, in human medicine a higher rate of UTIs is observed in women and the highest UTIs rates are seen patients from 18-65 years of age[32].

Of the *Escherichia coli* isolates obtained from either mono- or polymicrobial canine UTIs, 45% were defined as multidrug resistant (MDR; resistant to ≥ 3 antimicrobial classes) (Fig 1B) and 33% demonstrated resistance to beta-lactam antimicrobials based on the presence of *blaCMY* and/or *blaTEM* genes (Fig 1C). In the human literature, 49-68% of UPEC are classified as MDR and 24-40% were extended spectrum beta-lactamase (ESBL) producers[34–36].

Canine patients diagnosed with a recurrent UTI, defined as more than 1 positive urine culture within 6 months of the first urine culture and with the same uropathogen despite antimicrobial therapy, occurred in 42% of cases (Fig 1D). Comparable recurrence rates of 27-53% are observed in human UTIs [28,32]. In parallel to human studies, the most common bacterial

pathogens causing recurrent UTIs in our retrospective analysis were *Escherichia coli* (51.6%) and *Enterococcus* spp. (20.8%) (Fig 1E)[33].

Fig 1. Incidence of various uropathogens in canine urinary tract infections. A retrospective analysis of positive urine cultures submitted to the Clinical Microbiology Laboratory at the North Carolina State University College of Veterinary Medicine were reviewed over a 5-year period (2015-2019). (A) Pathogen incid[illegible]e of all positive urine cultures (n=797). Corresponding medical records were reviewed to determine if the canine patient was experiencing a recurrent UTI defined by more than one positive culture of the same uropathogen within 6 months of one another. (B) Recurrence rates observed over the 5-year period of the retrospective (C) Pathogen incidence or urine cultures from a recurrent infection. Antimicrobial susceptibility profiles and gene expression for several ESBL genes (standard screening with the Clinical Microbiology Laboratory for any *Escherichia coli*) were reviewed. (D) Incidence of multidrug resistance in the *Escherichia coli* population (n=312) only defined by resistance to greater than or equal to three antimicrobial drug classes. (E) Incidence of at least 1 positive ESBL gene in the *Escherichia coli* population only.

IBCs and filamentous bacteria are observed on urine cytologies and bladder biopsies from dogs diagnosed with UPEC associated UTIs

During human and murine-model UPEC pathogenesis, the bacteria bind to and invade into the cells of the urothelium where they replicate and form IBCs[11,16,37]. After IBCs mature, they will disperse out of the cell to infect neighboring facet cells[38]. When the bacteria emerge from the urothelial cell, they adopt a filamentous phenotype. Both IBCs and filamentous bacteria have been identified in the urine of humans with UTIs[27]. To determine if dogs with naturally occurring UTIs exhibit the presence of IBCs and filamentous bacteria, we evaluated urine

cytologies (n=30) and bladder biopsies (n=10) from dogs with UPEC associated UTIs. Immunofluorescence was used to identify IBCs and filamentous bacteria (Fig 2). In our analysis of urine cytologies by immunofluorescence, we found UPEC IBCs in 60% of the cytologies evaluated (Fig 2A; 18 of 30) and observed filamentous bacteria in 77% of the cytologies evaluated (Fig 2B; 23 of 30). Specifically, IBCs (anti-*E. coli*, green) were appreciated within sheets of urothelial cells (anti-uroplakin III, purple) (Fig 2CD) and individual urothelial cells (Fig 2EF). We also identified filamentous bacteria dispersing from the urothelium-associated IBCs (Fig 2GH). Upon evaluation of the bladder biopsies, we observed IBCs (anti-*E. coli*, green) within the urothelium (anti-Ecadherin, red) in 4 out of the 10 dogs evaluated (Fig 3). The morphology of the IBCs observed in these clinical canine samples reflect those seen in humans and mice[21].

Fig 2. Canine urine cytologies show evidence of IBCs and filamentous bacteria. Urine cytologies were evaluated from dogs exhibiting clinical symptoms of a UTI and a positive *Escherichia coli* culture (n=30). The cytologies were made by the Clinical Pathology Laboratory at North Carolina State University's College of Veterinary Medicine according to their standard operating procedures. The cytologies were then fixed, permeabilized, blocked and stained with anti-Escherichia coli FITC (green) antibody, an anti-uroplakin IIIB antibody with an AlexaFluor 647 secondary antibody (purple) (Abcam, Cambridge, UK) and DAPI (blue) to stain nuclei. (A) The number of urine cytologies evaluated that had observable IBCs within shed urothelial cells. (B) The number of urine cytologies evaluated that had observable filamentous bacteria. (C,D) Lower magnification of large urothelial cell sheets containing both IBCs and filamentous bacteria. (E,F) Higher magnification with white arrows pointing to discrete IBCS within single shed urothelial cells. (G,H) Higher magnification with white arrows pointing to filamentous bacteria emerging from the urothelium.

Fig 3. IBCs are observed within the urothelium of dogs with UPEC-causing UTIs. (A-D) Representative images of IBCs noted within the bladder epithelium of four different canine patients. Briefly, bladder biopsies of dogs presenting to the Internal Medicine Service at North Carolina State University's College of Veterinary Medicine (NCSU-CVM) were evaluated. Biopsies were only evaluated from dogs with clinical signs of a UTI and a positive UPEC culture. Paraffin-embedded sections underwent heat induced antigen retrieval followed by staining with the following antibodies: E-cadherin with an AlexaFluor 555 secondary (red), Escherichia coli-FITC (green) and DAPI to stain nuclei (blue).

Canine urothelial cell cultures support the formation of IBCs by clinical UPEC isolates

Previous research has shown that an immortalized human bladder epithelial cell line (PD07i) supports the growth of IBCs by UPEC *in vitro*[39]. Our aim was to develop an *in vitro* canine UTI model using canine primary urothelial cells and clinical canine UPEC strains. Urothelial cells were harvested and cultured from canine bladders collected post-mortem from healthy dogs without signs of urinary or kidney disease as previously described (Fig S1)[40]. Primary canine urothelial cells (n=3) were then infected with UPEC strains from symptomatic canines with confirmed UTIs (n=30). IBCs were observed 24 hours post-infection (anti-*E. coli*, green) within cultured urothelial cells (F-actin, phalloidin, red) (Fig 4). Specifically, we observed several of the UPEC strains forming IBCs within our canine urothelial cell model with an average of 5.8±5.15 IBCs observed per high powered field (HPF) (Fig 4A) and an average of 4.3±2.63 $\log_{10}$CFU per well (Fig 4B). Five of the 30 (16.7%) UPEC isolates evaluated had no observable IBCs, correlating with <1 $\log_{10}$CFU/mL (Fig 4AB). The IBCs of the canine clinical UPEC strains

grown in canine primary urothelial cells exhibited similar morphology to the IBCs of human UPEC strains grown in an immortalized bladder cell line (Fig 4C-H)[41,42].

Fig S1. Canine urothelial cell isolation and differentiation. Canine bladders (n=10) were aseptically collected postmortem from apparently healthy dogs after planned euthanasia by an animal control facility. The bladder was removed by severing of the ureters and ligaments and excising the bladder at the neck. The bladder was turned inside out to expose the mucosa. The neck of the bladder was sutured closed to ensure only mucosa was exposed. (A) The tissue was then suspended in an Erlenmeyer flask in 200mL HBSS containing antibiotic-antimycotic and 2.4U/mL Dispase II and incubated for 2 hours at 5% CO2, 90% humidity, and 37°C under constant rotation. After 2 hours, any remaining tissue on the mucosal surface was gently scraped off. The cell were pelleted by centrifugation, washed and resuspended in fresh HBSS. Cells were then seeded onto collagen coated flasks at 40,000 cells/cm2 in UCPM. The culture medium was changed every 48 hours for 5-7 days. UCPM is a serum-free media and was used to proliferate urothelial cells while decreasing fibroblast contamination. (B) By 7 days a pure urothelial cell culture was appreciated by brightfield microscopy (10x) and analysis of cellular morphology as described by others[37,63,65]. Urothelial cells were frozen in aliquots and stored in liquid nitrogen until future use. (C) Canine urothelial cells were thawed, and seeded collagen coated 24-well tissue culture plates in UCPM. Cells were grown for 72 hours, or until confluency. Thereafter, the culture media was changed to promote urothelial cell differentiation for expression of uroplakins required for UPEC pathogenesis (termed UCDM) as described elsewhere[39,83,84]. Cells were cultured in UCDM for 48 hours, followed by a media change, and then cultured for an additional 48 hours. Expression of various uroplakins was determined by real-time polymerase chain reaction using

canine specific primers and the housekeeping gene GAPDH. Relative expression compared to the day 0 (before changing to UPDM) is presented at $2^{-\Delta\Delta Ct}$.

Fig 4. Canine primary urothelial cells support IBC formation by canine origin UPECs. Urothelial cells isolated from healthy canine bladders (n=3) were infected with clinical UPEC isolates from cases of canine symptomatic UTIs (n=30) in triplicate for each canine donor. Two hours after infection, gentamicin was added to kill any remaining extracellular bacteria. The infected cultures were then incubated for another 22 hours. Twenty-four hours post-infection, the cultures were washed gently with warm PBS and fixed with paraformaldehyde. After fixation, the cells were permeabilized and blocked prior to staining with an anti-Escherichia coli FITC antibody (green). Cells were also stained with phallodin (red) to stain actin and DAPI (blue) to stain nuclei. (A) The number of IBC per high powered field (HPF) were counted for each donor/UPEC combination. The graph represents the average number of IBCs for each UPEC isolate counted in each donor and in each replicate. (B) The bacterial load per well was measured by lysing the cells in a second culture that was unfixed to release the intracellular bacteria. Each well was serial diluted and plate counted for colony forming units (CFUs). Data is represented as a log CFU per well. (C-E) High-powered representative images (20x) of IBCs observed in infected canine urothelial cells with various canine origin UPEC stains. (F-H) Low-powered representative images (10x).

IBC formation in canine urothelial cell culture is dependent upon *fimH*

Previous research in humans has shown that IBC formation is dependent upon the presence and expression of type 1 pilus or *fimH* in UPEC[12,43]. Therefore, we infected canine urothelial cells from three donors (n=3) with clinical canine UPEC strains that were *fimH* positive (n=9) or

negative (n=9) to determine the association with IBC formation. We found that clinical UPEC isolates containing the *fimH* gene formed significantly more IBCs in canine urothelial cells (7.7 IBCs/HPF) as compared to isolates lacking the *fimH* gene (0.5 IBCs/HPF) as compared (Fig 5A, $p<0.0001$). *FimH* status also affected the bacterial load within urothelial infections, with *fimH* positive isolates establishing a higher CFU/well (5.1 $\log_{10}$CFU/well) as compared to *fimH* negative isolates (0.8 $\log_{10}$CFU/well) (Fig 5B, $p<0.0002$). Representative images of urothelial infections from a *fimH*- UPEC infection (Fig 5C) and *fimH*+ UPEC infection (Fig 5D) further demonstrate that canine origin UPEC stains lacking *fimH* are unable to establish robust IBCs in canine urothelial cells which correlates with results seen in the murine model[12,44,45].

Fig 5. Canine UPEC isolates lacking *fimH* do not form robust IBCs in comparison to isolates that harbor an intact *fimH* gene. Urothelial cells from canine donors (n=3) were isolated, cultured, infected and evaluated as in Fig 4. Specifically, canine urothelial cells were infected with canine UPEC isolates lacking the *fimH* gene (n=9) or those that harbored the *fimH* gene (n=9). The presence or absence of the *fimH* gene was confirmed by PCR as previously described [23]. (A) The number of IBCs per high powered field (HPF) were counted for each donor/UPEC combination. The graph represents the average number of IBCs for each UPEC isolate counted in each donor and in each replicate. (B) The bacterial load per well was measured by lysing the cells in a second culture that was unfixed to release the intracellular bacteria. Each well was serial diluted and plate counted for colony forming units (CFUs). Data is represented as a log CFU per well. (C) Representative images of a urothelial cell infection by a *fimH*- UPEC. (D) Representative images of a urothelial cell infection by a *fimH*+ UPEC. Bars are means and standard deviations and significant differences ($p<0.05$) were determined by an unpaired, nonparametric t-test (for IBCs/HPF) and parametric t-test (for logCFU/mL).

Canine urothelial cell IBC formation is more robust in UPEC isolates derived from cases of recurrent UTIs

To date, the genotypic or phenotypic relationship between UPEC strains and recurrent UTIs is unknown. Due to the variability in clinical UPEC isolates ability to form IBCs, we hypothesized that UPEC strains from recurrent UTI cases would form more IBCs *in vitro*. Therefore, we evaluated UPEC strains from recurrent cases (n=12) of symptomatic UTIs treated with appropriate antimicrobials with at least once recurrence within 6 months of the first UTI. We compared these recurrent UPEC strains with matched UPEC strains from non-recurrent cases of symptomatic UTIs. The non-recurrent cases all had well documented follow-up to ensure a second UPEC-associated UTI did not occur within 6 months of the initial UTI. We also tested for the presence of the *fimH* gene within this group. Interestingly, UPEC isolates from recurrent UTIs expressed more *fimH* (97.5%) versus UPEC isolates from non-recurrent cases (84.9%) (Fig S2A). Therefore, we only used UPEC isolates that were *fimH* positive to compare IBC formation between UPEC isolates from recurrent and non-recurrent cases. We observed a significant difference in both number of IBCs and the size of the IBCs from recurrent UPEC strains. UPEC strains from recurrent cases formed more IBCs (12.8 IBCs/HPF) as compared to UPEC from non-recurrent cases (5.5 IBCs/HPF) (Fig 6A, $p<0.001$). UPEC strains from recurrent cases also had a higher intracellular bacterial load per well (7.6 $\log_{10}$CFU/well) then UPEC from non-recurrent cases (4.2 $\log_{10}$CFU/well) (Fig 6B, $p<0.004$). Representative images of urothelial infections show that UPECs from non-recurrent cases (Fig 6C) form less IBCs then UPECs from recurrent cases (Fig 6D). As all the UPEC strains from recurrent and non-recurrent cases were confirmed *fimH*+, further investigation into the mechanisms behind the increased ability of recurrent UPEC strains to form robust IBCs is warranted as *fimH* is not the only gene involved in IBC formation.

Fig S2. Canine UPEC isolates from recurrent UTIs correlate with fimH gene expression but not multidrug resistance (MDR). Canine UPEC isolates (n=-206) were classified as recurrent with well-documented recurrence of the same strain within 6 months of the initial UTI. (A) Each isolate was evaluated for the presence or absence of the *fimH* gene was confirmed by PCR as previously described [23]. (B) Incidence of multidrug resistance was defined by resistance to greater than or equal to three antimicrobial drug classes. Bars are means and standard deviations and significant differences ($p<0.05$) were determined by an unpaired, nonparametric t-test.

Fig 6. Canine UPEC isolates from patients with recurrent UTIs form more robust IBCs then isolates from patients with non-recurrent UTIs. Urothelial cells from canine donors (n=3) were isolated, cultured, infected, and evaluated as in Fig 4. Specifically, canine urothelial cells were infected with canine UPEC isolates from cases with a well-documented recurrence of the same strain within 6 months of the initial UTI (n=12). We matched these UPEC isolates with those from non-recurrent cases with well document follow-up in their medical records to ensure that a second UPEC-causing UTI did not occur within 6 months of the initial episode (n=12). (A) The number of IBCs per high powered field (HPF) were counted for each donor/UPEC combination. The graph represents the average number of IBCs for each UPEC isolate counted in each donor and in each replicate. (B) The bacterial load per well was measured by lysing the cells in a second culture that was unfixed to release the intracellular bacteria. Each well was serial diluted and plate counted for colony forming units (CFUs). Data is represented as a log CFU per well. (C) Representative images of a urothelial cell infection by a non-recurrent UPEC isolate. (D) Representative images of a urothelial cell infection by recurrent UPEC isolate. Bars are means and

standard deviations and significant differences ($p<0.05$) were determined by an unpaired, nonparametric t-test (for IBCs/HPF) and parametric t-test (for logCFU/mL).

IBCs in canine urothelial cell culture are tolerant to antimicrobial treatment

Both *in vitro* and *in vivo* studies have shown that UPEC IBCs persist intracellularly despite treatment with antimicrobials[6,46]. In the clinical setting, dogs with recurrent UTIs typically receive first-line antimicrobials, which despite demonstrating *in vitro* susceptibility, may result in persistent infections, and the dog will remain culture-positive. Therefore, we wished to investigate the antimicrobial tolerance of IBC-forming UPECs to antimicrobials commonly used to treat canine UTIs. We infected canine primary urothelial cells from three separate donors (n=3) with three UPEC isolates (n=3) known to form robust IBCs and allowed IBCs to develop for 24 hours. Subsequently, we treated with infected urothelial cells cultures with enrofloxacin (ENRO, 150µg/mL), cefpodoxime (POD, 100 µg/mL) and doxycycline (DOX, 50µgmL) at the Cmax observed in urine in canine pharmacokinetic studies[47–49]. All the UPEC clinical isolates used for the urothelial infections were susceptible to these antimicrobials as determined by *in vitro* susceptibility testing. We found that no antimicrobial treatment tested could eradicate IBCs considerably at concentrations at least 10x the minimum inhibitory concentration (MIC), (No Treatment (NT) 9.1 IBCs/HPF versus enrofloxacin (ENRO) 5.2 IBCs/HPF, cefpodoxime (POD) 7.9 IBCs/HPF, and doxycycline (DOX) 6.7 IBCs/HPF) (Fig 7A). Additionally, we did not appreciate a greater than 2 log reduction in bacterial load (NT 5.3 log_{10}CFU/well versus ENRO 3.8 log_{10}CFU/well, POD 4.7 log_{10}CFU/well, and DOX 4.1 log_{10}CFU/well, respectively) (Fig 7B). Representative images showing the presence of IBCs (Fig 7C-F) despite antimicrobial treatment at well above the MIC. These results show that, similar to traditional biofilms, IBCs grown in

canine urothelial cells are tolerant to antimicrobials and therefore are likely to persist intracellularly in the face of antimicrobial treatment.

Fig 7. IBCs formed in canine urothelial cells by canine-origin canine UPECs are tolerant to treatment with several antimicrobials from different classes. Urothelial cells from canine donors (n=3) were isolated, cultured, infected and evaluated as in Fig 4. Specifically, canine urothelial cells were infected with canine UPEC isolates known to form robust IBCs based those in Fig 4 (n=3) and susceptible to the antimicrobials tested herein. (A) The number of IBCs per high powered field (HPF) were counted for each donor/UPEC/treatment combination. The graph represents the average number of IBCs for each UPEC isolate counted in each donor and in each replicate. (B) The bacterial load per well was measured by lysing the cells in a second culture that was unfixed to release the intracellular bacteria. Each well was serial diluted and plate counted for colony forming units (CFUs). Data is represented as the average log CFU per well. (C) Representative images of an untreated urothelial cell infection. (D) Representative images of urothelial cell infection treated with 150 µg/mL enrofloxacin (ENRO). (E) Representative images of urothelial cell infection treated with 50 µg/mL cefpodoxime (POD). (F) Representative images of urothelial cell infection treated with 50 µg/mL doxycycline (DOX). Bars are means and standard deviations and significant differences ($p<0.05$) as determined by ANOVA with Tukey post-hoc are indicated by differing letters

Discussion

We established the ability of canine-derived uropathogenic *Escherichia coli* (UPEC) to form intracellular bacterial communities (IBCs) in canine urothelial cells. This study also demonstrated the feasibility of the dog as a translational model of human urinary tract infections (UTIs). Using a 5-year retrospective analysis, we found that UTIs in dogs are dominated by similar

uropathogens as those found in human UTIs with similar frequencies of antimicrobial resistance, multidrug resistance, and recurrent infection as human UTIs[28–30,34–36]. Next, we evaluated and observed IBCs in urine cytologies and biopsies from dogs with positive *E. coli* urine cultures. After validating the clinical appearance of IBCs in dogs, we sought to determine if UPECs of canine origin could establish IBCs in canine urothelial cells *in vitro*. We discovered that canine UPEC isolates formed robust IBCs in canine urothelial cells and that IBC formation was dependent upon *fimH*. Canine UPEC isolates from recurrent cases formed more robust IBCs then those from non-recurrent cases. Lastly, we also found that IBCs were tolerant to treatment with several classes of antimicrobials when used at concentrations achievable in canine urine after standard dosing. Overall, the results of this manuscript show the potential of the dog as a model of human UTIs and demonstrates a similar pathogenesis in canine UPEC UTIs as in human UPEC UTIs. Future experiments using an *in vivo* canine model of IBCs will be required to validate the model for pathogenesis and drug development studies.

Urinary tract infections (UTI) are one of the most common bacterial infections in both humans and dogs[1,50]. Uropathogenic Escherichia coli (UPEC) account for >75% of human UTIs[28]. We found that UPEC accounted for 40% of canine urinary tract infections. Although UPEC rates were lower in dogs, UPEC is still the main uropathogen causing UTIs in dogs. Recurrent infections are a major concern during the treatment and management of UTIs in both dogs and humans[4,51]. In fact, approximately 20-30% of women with UTIs will develop a recurrent infection within 6 months of the initial UTI[52]. In dogs, we found a slightly higher recurrence rate of 42% which may be due to the case selection: all cases were from a referral hospital and not a primary care clinic. UPEC account for most human recurrent infections associated with the same UPEC strain in >65% of human recurrent UTIs[32,52]. Similarly, in

dogs we found that UPEC accounted for the majority (52%) of recurrent cases. In recent studies, multi-drug resistant UPEC strains are identified in >60% of human isolates[53,54]. We found that 45% of canine UPEC isolates were resistance to at least 3 classes of antimicrobials. In addition, extended spectrum beta-lactamase production (ESBL) has been identified in >30% of human UPEC isolates[36,53]. In our canine population, we observed 33% of canine UPEC isolates harboring one or more beta-lactamase or ESBL associated genes.

In both human and murine UTI studies, the formation of intracellular bacterial communities (IBCs) within the urothelium is key to UPEC pathogenesis[55]. UPEC readily attach, invade, and colonize urinary epithelial cells[11,56] and this ability is often implicated in recurrent UTIs[57,58]. A study in mice showed UPEC persisting within the bladder epithelium and causing recurrence in one third of the mice[59]. A publication from Rosen et al,. 2007 was the first to show IBCs within sloughed urothelial cells from urine of women with UTI symptoms[27]. In that study they found evidence of IBCs in 1 out of 5 urine samples and filamentous bacteria, which are associated with the IBC lifecycle, in 50% of the urine samples. We evaluated urine samples from dogs with UTI symptoms and a positive UPEC culture. We found evidence of IBC formation and filamentous bacteria in 60% and 77% of urine cultures, respectively. We speculate that the higher rates of IBCs and filamentous bacteria observed in our canine study was because we only evaluated dogs with a positive UPEC culture while Rosen et al., 2007 evaluated UTIs caused by a variety of uropathogens. Other uropathogens such as *Klebsiella pneumoniae* and *Proteus mirabilis* can also form IBCs, but no evidence of IBC formation in gram-positive species has been reported[60–62]. We also evaluated canine bladder biopsies for the presence of IBCs to visualize IBCs i*n situ* versus sloughed urothelial cells in urine only. We found evidence of IBCs within the urothelium of the bladder of dogs with a positive UPEC culture and UTI symptoms. These IBCs were similar in

structure and appearance to the IBCs seen within murine urothelium during experimental UPEC infection[11,27].

Other research groups have shown that human primary urothelial cells, human urothelial cells lines and bladder-on-chip models are able to support IBC formation by UPECs[41,63–65]. Therefore, we hypothesized that canine urothelial cells might be able to support IBC formation as well. Indeed, we demonstrated that most UPEC isolates were able to form IBCs in our *in vitro* canine urothelial cell model. These results correlate well with a murine study showing that most human derived UPECs could form IBCs in a murine *in vivo* UTI model[12,59,64].

The ability of UPEC to adhere to and invade into the urothelium to form IBCs is facilitated by the type 1 pilus-associated adhesin FimH, a protein encoded by the *fimH* gene, and which binds to mannosylated uroplakin-proteins that line the apical surface of urothelial cells in the bladder, triggering bacterial internalization about 3-hours post-infection[12,16,37,38]. Other studies in mice have shown that UPEC lacking the *fimH* gene are unable to form IBCs[12,43]. Therefore, we investigated the ability of canine derived UPEC harboring or lacking the fimH gene to from IBCs in our canine urothelial cell model. We found that UPEC isolates lacking the *fimH* gene formed fewer IBCs than those isolates with harboring the *fimH* gene. However, in those UPEC isolates harboring the *fimH* gene we observed a range of IBCs from rare to robust.

We also found that UPECs isolated from recurrent UTI cases formed more IBCs then those UPEC isolates from non-recurrent cases. Other studies have shown that certain SNPs within the *fimH* gene can results in a gain or loss of function in binding to urothelial cells[44,45,69]. If UPEC are unable to bind to the urothelial cell, they are subsequently unable to invade into the cell to form IBCs. Therefore, we hypothesize that this variation and association with recurrence could be due

to the FimH type or SNPs within the *fimH* gene resulting from pathoadaptation to the urinary environment which will be a line of inquiry for future work.

UPEC within IBCs are protected from killing by antimicrobials[6,17]. This protection arises from both decreased penetration of the antimicrobial into the bacterial community within the urothelial cell and the reduced metabolic state of bacteria within the IBC[63,70,71]. Therefore, we set out to determine if our canine derived UPECs forming IBCs with canine urothelial cells were protected from antimicrobial effect, including killing. We infected canine urothelial cells with UPEC strains know to form robust IBCs from previous screening for 24 hours prior to treatment with a variety of first-line antimicrobials from different drug classes used to treat canine UTIs[72,73]; specifically, the fluoroquinolone enrofloxacin, the cephalosporin cefpodoxime, and the tetracycline doxycycline. We challenged the UPEC-infected urothelial cells with concentrations achievable in canine urine after standard dosing of each antimicrobial[74–76]. We found that none of the antimicrobials at clinically relevant concentrations were able to substantially reduce the number of IBCs or the bacterial load greater than 2-log. These results are supported by several studies showing that IBCs are tolerant to antimicrobial treatment[6,17,77]. For example, in a murine bladder infection model treatment with trimethoprim-sulfamethoxazole did not eradicate bacteria within the bladder[17]. Another study showed persistence of UPEC within the bladder epithelium using multiple antibiotics with urine concentration well above the therapeutic threshold of planktonic (single cell) UPEC[6]. In a bladder on chip model, treatment with a beta-lactam, ampicillin, did not reduce the IBC size or distribution within the tissue[46]. These findings correlate with the clinical observations that approximately 14% of women require a second antibiotic prescription within 28 days of diagnosis[77].

In conclusion, we found that the dog is a robust translational model for human UTIs. A recent review article calls for the development of new animal models that better recapitulate human disease[78]. As the dog is a naturally occurring model of UPEC UTIs it holds promise as a more clinically relevant model then the induced murine or porcine models currently in use[79–83]. The results of this study show that the pathogenesis of canine UPEC UTIs, with the formation of antimicrobial tolerant *fimH* dependent IBCs, are similar to human UTIs caused by UPECs. Importantly, the use of the dog would serve as a One Health model as results from these studies could directly apply to both human and veterinary medicine. However, future work is needed that expands upon the presented in this manuscript into an *in vivo* experimental model that can be used to study pathogenesis and develop novel therapeutics. In addition, direct genomic comparisons of canine and human UPECs could be useful in further assessing the canine UTI model.

Methods

Bacterial strains

Clinical UPEC isolates (n = 206) were obtained from routine clinical aerobic cultures of canine urine submitted to the North Carolina State University's College of Veterinary Medicine (NCSU-CVM), Microbiology and Molecular Diagnostics Laboratory (Raleigh, NC) at the discretion of the treating clinician. Isolates were included if 1) *E. coli* was the sole etiological agent (monospecies) 2) urine samples were collected via cystocentesis 3) the quantity of *E. coli* recovered was greater than 100,000 CFU/mL as determined by plating of a standard urine volume and 4) the dog was considered to have a UTI based on clinical presentation and diagnosis by the attending veterinarian. All isolates were confirmed as *E. coli* using matrix assisted laser desorption/ionisation time of flight mass spectrometry (MALDI-TOF)(Vitek MS, Biomerieux;

Marcy-l'Étoile, France). Isolates were saved as frozen stocks on glycerol at -80°C. Antimicrobial susceptibility testing was performed on all isolates using the broth microdilution method by a commercial system for the clinical report (Sensititre™; ThermoFisher Scientific, Waltham, MA) using the Companion Animal MIC Plate (Companion Animal MIC Plate). The minimum inhibitory concentrations (MIC) of each antimicrobial reagent were classified as susceptible (including intermediate) or resistant based on the Clinical & Laboratory Standards Institute (CLSI) breakpoints when available[85]. Isolates are characterized for the following UPEC-associated virulence genes attributed to extraintestinal *E. coli* (ExPEC): *cnf-1, hlyA, fimH* and *papG* using previously described PCR assays[26].

Recurrent UTI Classification

Medical records of the North Carolina State University (Raleigh, NC) Veterinary Teaching Hospital (VTH) were searched to identify UPEC isolates from dogs that were diagnosed with recurrent UTIs. Isolates were designated as recurrent isolates based on at least ≥2 UTIs confirmed by positive aerobic bacterial cultures of *E. coli* during a 6-month period.

Clinical Specimens

Urine cytologies and bladder biopsies were sourced from North Carolina State University, College of Veterinary Medicine, Clinical Pathology, Immunology and Histopathology Laboratories (Raleigh, NC). Cytologies and biopsies were submitted at the discretion of the treating clinician as part of a clinical work-up and were not submitted for inclusion in the study. Retrospectively, cytologies and biopsies were chosen for evaluation if the dog had a concurrent positive *Escherichia coli* monoculture by NCSU-CVM's Clinical Microbiology and Molecular Diagnostics Laboratory, presented with a symptomatic UTI, and had evidence of bacteria by Diff-Quick staining for cultures as assessed by a qualified clinical pathology technician and evidence

of inflammation and infection on histology as assessed by a board-certified clinical pathologist. Cytologies and biopsies were then assessed for the presence of IBCs by immunofluorescence. For cytologies (n=30), prepared cytospin slides were fixed with 2% paraformaldehyde, washed with sterile phosphate-buffered saline (PBS) and permeabilized with 1% Bovine Serum Albumin (BSA)/0.3% Triton X-100 for 1 hour at room temperature and blocked for 1 hour with 5% BSA. Slides were then incubated overnight with Rabbit Anti-*E. coli* FITC antibody (1:100, ab30522, Abcam, Cambridge, UK) and Mouse Anti-Uroplakin III primary antibody (1:300, ab78196, Abcam, Cambridge, UK). Slides were washed three times in sterile PBS and incubated with the secondary antibody Goat Anti-Mouse IgG H&L Alexa Fluor® 647 (1:1000, ab150115, Abcam, Cambridge, UK) for 1 hour. Slides were then washed again with sterile PBS and mounted with DAPI Fluoromount-G (Southern Biotech, Birmingham, AL). For biopsies (n=10), paraffin-embedded tissue was deparaffinized with Histo-Clear II® (Electron Microscopy Sciences, VWR International, Radnor, PA) followed by ethanol dehydration and heat induced antigen retrieval. Slides were then permeabilized in 1% BSA/0.3% Triton X-100 for 10 minutes and blocked for 1 hour with 5% BSA. Slides were then incubated overnight with Rabbit Anti-*E. coli* FITC antibody (1:100, ab30522, Abcam, Cambridge, UK) and Rabbit Anti-E Cadherin (1:250, ab15148, Abcam, Cambridge, UK). Slides were washed three times in sterile PBS and the secondary antibody Goat Anti-Rabbit IgG H&L Alexa Fluor® 555 (ab150078) (1:1000, ab150115, Abcam, Cambridge, UK) for 1 hour. Slides were then washed again with sterile PBS and mounted with DAPI Fluoromount-G (Southern Biotech, Birmingham, AL). Stained cytologies and biopsies were imaged on an Olympus IX73 inverted scope with DP80 camera using appropriate fluorescent channels (Olympus Corporation, Shinjuku, Tokyo, Japan).

Canine Urothelial Cell Isolation

Whole canine bladders (n=10) were aseptically collected postmortem from apparently healthy dogs after planned humane euthanasia unrelated to our study by animal control facility personnel using facility established protocols. All dogs were euthanized by the animal control facility for reasons not reported to the study team, and patients were not recruited by study personnel. No decision, input, or demographics were provided from the study team; IACUC approval was not required as the animals were deceased at the time of sample collection. The use of these deceased patients as the bladder cell source was due to the invasive sample collection process, number of cells needed to establish a cell culture model and the biological importance of a bladder to living patients; we also considered the ability to preserve cell integrity by collecting immediately postmortem. The study team was notified the morning of a planned euthanasia and would respond if we could ensure that a specimen would be made available and our laboratory could immediately utilize the specimen. At the time of collection, sterile urine was collected for culture after surgical incision into the distal abdomen by cystocentesis. The bladder was removed by severing of the ureters and ligaments and excising the bladder at the neck before placing the bladder in PBS containing antibiotic-antimycotic (Gibco® Antibiotic-Antimycotic ThermoFisher Scientific, Waltham, MA). Bladders were transported to the laboratory on ice and all subsequent work was completed in a biosafety cabinet. The bladder was turned inside out to expose the mucosa. Mucosa was evaluated for any noticeable gross pathology. If a bladder had a positive urine culture or the mucosa appeared grossly abnormal, the bladder or any isolated cells were excluded from further use. The mucosa neck of the bladder was sutured closed to ensure only mucosa was exposed. The tissue was then suspended in an Erlenmeyer flask in HBSS containing antibiotic-antimycotic and 2.4U/mL Gibco® Dispase II (ThermoFisher Scientific, Waltham, MA) and incubated for 2 hours at 5% CO_2, 90% humidity, and 37°C under constant rotation (Fig S1A).

After 2 hours, any remaining tissue on the mucosal surface was gently scraped off and added to the dispersed cells, passed through a 100μm filter, and centrifuged at 300g for 10 min. The cell pellet was washed and resuspended in fresh HBSS and a live cell count was determined using a Cellometer® Auto 2000 and ViaStainTM AOPI Staining Solution (Nexcelom Bioscience LLC, Lawrence, MA). Cells were then seeded onto Nunc™ Collagen I Coated EasYFlasks™ (ThermoFisher Scientific, Waltham, MA) at 40,000 cells/cm^2 in Urothelial Cell Proliferation Media (UCPM, Gibco™ Defined Keratinocyte SFM containing Defined Keratinocyte-SFM Growth Supplement and antibiotic-antimycotic) and incubated overnight at 5% CO2, 90% humidity, and 37°C. Dead or non-adherent cells were removed the next day by washing with warm PBS three times and adding fresh UCPM. The culture medium was changed every 48 hours for 5-7 days. UCPM is a serum-free media and was used to proliferate urothelial cells while decreasing fibroblast contamination; by the end of 5-7 days, depending on the donor, a pure urothelial cell culture was appreciated by brightfield microscopy and analysis of cellular morphology as described by others (Fig S1B) [40,66,68]. When cultures reached a confluency of 75-95% pure morphologically epithelial-like cells, cells were detached from the growth surface with the use of Invitrogen™ Accutase (0.12 mL/cm^2) (ThermoFisher Scientific, Waltham, MA). Cells were counted again as above and frozen in aliquots of 1×10^6 cells/mL in liquid nitrogen until future use.

Canine Urothelial Cell Infection and Antimicrobial Treatment

Canine urothelial cells were thawed and seeded at 40,000 cells/cm^2 onto collagen coated 24-well tissue culture plates (Nunc™ Collagen I Coated Multidishes, ThermoFisher Scientific, Waltham, MA) in UCPM for 24 hours. Dead or non-adherent cells were removed the next day by washing with warm PBS three times and adding fresh UCPM. Cells were grown for another 48

hours, or until 75-95% confluence was reached. Thereafter, the culture media was changed to promote urothelial cell differentiation for expression of uroplakins required for UPEC pathogenesis as described elsewhere[42,86,87]. Briefly, media was changed to Urothelial Cell Differentiation Media (UCDM, 3 parts Dulbecco's modified Eagle's medium to 1-part F12 containing 5% fetal bovine serum, antibiotic-antimycotic and 2mM calcium). Cells were cultured in UCDM for 48 hours, followed by a media change, and then cultured for an additional 48 hours. We have shown that this time frame allows for optimal uroplakin expression in our model (Fig S1C). Thereafter, urothelial cells (n=3 biological replicates per experiment) were infected with various individual UPEC strains (n=30) described above in "Bacterial strains" at a multiplicity of infection (MOI) of 10:1 for 3 hours at 37°C and 5% CO_2. After 3 hours, cells were washed five times with sterile PBS and incubated with media containing 100 µg/ml gentamicin to kill any extracellular bacteria. Infections were carried out for 21 hours after the gentamicin treatment. For antimicrobial challenge experiments, 24 hours post-infection, the gentamicin containing-media was removed, and the cellular monolayer was washed five times with sterile PBS before media containing the appropriate antimicrobial was added for an additional 24 hours. Enrofloxacin (ENRO), cefpodoxime (POD) or doxycycline (DOX) were used at 150, 50 and 50 µg/mL respectively which reflect achievable canine urine concentrations[47–49].

Intracellular Bacterial Community (IBC) Analysis

IBCs were assessed by two methods. First, the intracellular bacterial load was assessed in each culture by lysing urothelial cells and performing serial dilutions and plate counting to determine intracellular CFU[39]. Second, we visualized and quantified IBCs by immunofluorescence. Briefly, after infection and/or treatment, cultures were washed five times with PBS and fixed with 2% paraformaldehyde. Cultures were then permeabilized with 0.3% Triton X-100 and blocked

with 5% BSA prior to staining with Rabbit Anti-*E. coli* FITC (1:100, ab30522, Abcam, Cambridge, UK) and Alexa Fluor™ 555 Phalloidin according to manufacturer's directions (ThermoFisher Scientific, Waltham, MA). Nuclei were stained with DAPI Fluoromount-G® (SouthernBiotech, Birmingham, AL) prior to imaging on an Olympus IX73 inverted scope with DP80 camera using appropriate fluorescent channels (Olympus Corporation, Shinjuku, Tokyo, Japan). To quantify the number of IBCs by visualization, the average number of IBCs observed per high powered field (HPF) were counted; five HPFs at 10x magnification were counted per well. A secondary quantification method was performed to determine the intracellular bacterial load. Using this method, urothelial cell monolayers were washed 3x with PBS and lysed with 0.1% Triton X for 10 minutes. After lysis, serial dilutions were performed, and spot plated in triplicate at each serial dilution for colony counts.

Statistical Analysis

Data was analyzed for normality by the Shapiro- Wilk test. Single data points such as CFU were analyzed using 1-way non-parametric (Kruskal-Wallis Test) or 1-way/2-way ANOVA based on normality with Tukey's post hoc tests. Sex and other covariates were included as fixed effects in the model if indicated. All of the analyses were performed using SAS 9.4 software.

Acknowledgements

The authors would like to thank Ms. Jasmin Huang and Ms. Melissa Byrd from the Clinical Microbiology Laboratory at North Carolina State University's College of Veterinary Medicine (NCSU-CVM), Ms. Laura Miller from the Histology Laboratory at NCSU-CVM, Ms. Lynnette McCall from the Clinical Pathology Laboratory at NCSU-CVM, and Ms. Cathryn Hubbard from the Clinical Skills Laboratory at NCSU-CVM.

References

1. Klein RD, Hultgren SJ. Urinary tract infections: microbial pathogenesis, host–pathogen interactions and new treatment strategies. Nat Rev Microbiol. 2020; 1–16. doi:10.1038/s41579-020-0324-0
2. Seguin MA, Vaden SL, Altier C, Stone E, Levine JF. Persistent Urinary Tract Infections and Reinfections in 100 Dogs (1989-1999). Sep, 2003 pp. 622–631. doi:10.1111/j.1939-1676.2003.tb02492.x
3. Mysorekar IU, Hultgren SJ. Mechanisms of uropathogenic Escherichia coli persistence and eradication from the urinary tract. Proceedings of the National Academy of Sciences. 2006;103: 14170–14175. doi:10.1073/pnas.0602136103
4. Norris C, Williams B, Ling G, Franti C, Johnson, Ruby A. Recurrent and persistent urinary tract infections in dogs: 383 cases (1969-1995). J Am Anim Hosp Assoc. 2000;36: 484–492. doi:10.5326/15473317-36-6-484
5. Robino L, Scavone P, Araujo L, Algorta G, Zunino P, Vignoli R. Detection of intracellular bacterial communities in a child with escherichia coli recurrent urinary tract infections. Pathog Dis. 2013;68: 78–81. doi:10.1111/2049-632X.12047
6. Blango MG, Mulvey MA. Persistence of uropathogenic Escherichia coli in the face of multiple antibiotics. Antimicrob Agents Chemother. 2010;54: 1855–63. doi:10.1128/AAC.00014-10
7. Chen SL, Wu M, Henderson JP, Hooton TM, Hibbing ME, Hultgren SJ, et al. Genomic diversity and fitness of E. coli strains recovered from the intestinal and urinary tracts of women with recurrent urinary tract infection. Sci Transl Med. 2013;5. doi:10.1126/SCITRANSLMED.3005497/SUPPL_FILE/5-184RA60_TABLES_S1_TO_S9.ZIP
8. Scott VCS, Haake DA, Churchill BM, Justice SS, Kim JH. Intracellular Bacterial Communities: A Potential Etiology for Chronic Lower Urinary Tract Symptoms. Urology. 2015;86: 425. doi:10.1016/J.UROLOGY.2015.04.002
9. Kern ZT, Jacob ME, Gilbertie JM, Vaden SL, Lyle SK. Characteristics of Dogs with Biofilm-Forming Escherichia Coli Urinary Tract Infections. J Vet Intern Med. 2018;32. doi:10.1111/jvim.15231
10. Hall-Stoodley L, Stoodley P. Evolving concepts in biofilm infections. Cell Microbiol. 2009;11: 1034–1043. doi:10.1111/j.1462-5822.2009.01323.x
11. Anderson GG, Palermo JJ, Schilling JD, Roth R, Heuser J, Hultgren SJ. Intracellular Bacterial Biofilm-Like Pods in Urinary Tract Infections. Science (1979). 2003;301: 105–107. doi:10.1126/science.1084550
12. Martinez JJ. Type 1 pilus-mediated bacterial invasion of bladder epithelial cells. EMBO J. 2000;19: 2803–2812. doi:10.1093/emboj/19.12.2803
13. Dias RCS, Moreira BM, Riley LW. Use of fimH single-nucleotide polymorphisms for strain typing of clinical isolates of Escherichia coli for epidemiologic investigation. 2010;48: 483–8. doi:10.1128/JCM.01858-09
14. Wang H, Min G, Glockshuber R, Sun T-T, Kong X-P. Uropathogenic E. coli adhesin-induced host cell receptor conformational changes: implications in transmembrane signaling transduction. J Mol Biol. 2009;392: 352–61. doi:10.1016/j.jmb.2009.06.077
15. Mulvey MA, Lopez-Boado YS, Wilson CL, Roth R, Parks WC, Heuser J, et al. Induction and Evasion of Host Defenses by Type 1-Piliated Uropathogenic Escherichia coli. Science (1979). 1998;282: 1494–1497. doi:10.1126/science.282.5393.1494
16. Anderson GG, Dodson KW, Hooton TM, Hultgren SJ. Intracellular bacterial communities of uropathogenic Escherichia coli in urinary tract pathogenesis. Trends Microbiol. 2004;12: 424–430. doi:10.1016/J.TIM.2004.07.005
17. Schilling JD, Lorenz RG, Hultgren SJ. Effect of trimethoprim-sulfamethoxazole on recurrent bacteriuria and bacterial persistence in mice infected with uropathogenic Escherichia coli. Infect Immun. 2002;70: 7042–9. doi:10.1128/iai.70.12.7042-7049.2002

18. Barber AE, Norton JP, Wiles TJ, Mulvey MA. Strengths and Limitations of Model Systems for the Study of Urinary Tract Infections and Related Pathologies. Microbiol Mol Biol Rev. 2016;80: 351–67. doi:10.1128/MMBR.00067-15
19. Leenaars CHC, Kouwenaar C, Stafleu FR, Bleich A, Ritskes-Hoitinga M, de Vries RBM, et al. Animal to human translation: A systematic scoping review of reported concordance rates. J Transl Med. 2019;17: 1–22. doi:10.1186/S12967-019-1976-2/FIGURES/11
20. Schneider B, Balbas-Martinez V, Jergens AE, Troconiz IF, Allenspach K, Mochel JP. Model-Based Reverse Translation Between Veterinary and Human Medicine: The One Health Initiative. CPT Pharmacometrics Syst Pharmacol. 2018;7: 65–68. doi:10.1002/PSP4.12262
21. Chiou WL, Jeong HY, Chung SM, Wu TC. Evaluation of using dog as an animal model to study the fraction of oral dose absorbed of 43 drugs in humans. Pharm Res. 2000;17: 135–140. doi:10.1023/A:1007552927404
22. Ogeer-Gyles J, Mathews K, Weese JS, Prescott JF, Boerlin P. Evaluation of catheter-associated urinary tract infections and multi–drug-resistant *Escherichia coli* isolates from the urine of dogs with indwelling urinary catheters. J Am Vet Med Assoc. 2006;229: 1584–1590. doi:10.2460/javma.229.10.1584
23. Windahl U, Holst BS, Nyman A, Grönlund U, Bengtsson B. Characterisation of bacterial growth and antimicrobial susceptibility patterns in canine urinary tract infections. BMC Vet Res. 2014;10: 217. doi:10.1186/s12917-014-0217-4
24. Wong C, Epstein SE, Westropp JL. Antimicrobial Susceptibility Patterns in Urinary Tract Infections in Dogs (2010-2013). J Vet Intern Med. 2015;29: 1045–1052. doi:10.1111/jvim.13571
25. Johnson JR, O'Bryan TT, Low DA, Ling G, Delavari P, Fasching C, et al. Evidence of Commonality between Canine and Human Extraintestinal Pathogenic Escherichia coli Strains That Express papG Allele III. Infect Immun. 2000;68: 3327. doi:10.1128/IAI.68.6.3327-3336.2000
26. Gilbertie J, Levent G, Norman K, Vinasco J, Scott H, Jacob M. Comprehensive phenotypic and genotypic characterization and comparison of virulence, biofilm, and antimicrobial resistance in urinary Escherichia coli isolated from canines. Vet Microbiol. 2020;249. doi:10.1016/J.VETMIC.2020.108822
27. Rosen DA, Hooton TM, Stamm WE, Humphrey PA, Hultgren SJ. Detection of Intracellular Bacterial Communities in Human Urinary Tract Infection. Opal SM, editor. PLoS Med. 2007;4: e329. doi:10.1371/journal.pmed.0040329
28. Foxman B. The epidemiology of urinary tract infection. Nat Rev Urol. 2010;7: 653–660. doi:10.1038/nrurol.2010.190
29. Flores-Mireles AL, Walker JN, Caparon M, Hultgren SJ. Urinary tract infections: epidemiology, mechanisms of infection and treatment options. 2015;13. Available: http://www.ncbi.nlm.nih.gov/pubmed/25853778
30. Medina M, Castillo-Pino E. An introduction to the epidemiology and burden of urinary tract infections. Therapeutic Advances in Urology. SAGE Publications Inc.; 2019. doi:10.1177/1756287219832172
31. Kline KA, Lewis AL. Gram-Positive Uropathogens, Polymicrobial Urinary Tract Infection, and the Emerging Microbiota of the Urinary Tract. Microbiol Spectr. 2016;4. doi:10.1128/microbiolspec.uti-0012-2012
32. Foxman B. Epidemiology of urinary tract infections: incidence, morbidity, and economic costs. Am J Med. 2002;113 Suppl 1A: 5S-13S. doi:10.1016/s0002-9343(02)01054-9
33. Glover M, Moreira CG, Sperandio V, Zimmern P. Recurrent urinary tract infections in healthy and nonpregnant women. Urol Sci. 2014;25: 1–8. doi:10.1016/j.urols.2013.11.007
34. Ramírez-Castillo FY, Moreno-Flores AC, Avelar-González FJ, Márquez-Díaz F, Harel J, Guerrero-Barrera AL. An evaluation of multidrug-resistant Escherichia coli isolates in urinary tract

infections from Aguascalientes, Mexico: Cross-sectional study. Ann Clin Microbiol Antimicrob. 2018;17: 34. doi:10.1186/s12941-018-0286-5

35. Ali I, Rafaque Z, Ahmed S, Malik S, Dasti JI. Prevalence of multi-drug resistant uropathogenic Escherichia coli in Potohar region of Pakistan. Asian Pac J Trop Biomed. 2016;6: 60–66. doi:10.1016/j.apjtb.2015.09.022

36. Naziri Z, Derakhshandeh A, Borchaloee AS, Poormaleknia M, Azimzadeh N. Treatment failure in urinary tract infections: A warning witness for virulent multi-drug resistant ESBL-producing Escherichia coli. Infect Drug Resist. 2020;13: 1839–1850. doi:10.2147/IDR.S256131

37. Justice SS, Hung C, Theriot JA, Fletcher DA, Anderson GG, Footer MJ, et al. Differentiation and developmental pathways of uropathogenic Escherichia coli in urinary tract pathogenesis. Proceedings of the National Academy of Sciences. 2004;101: 1333–1338. doi:10.1073/pnas.0308125100

38. Lewis AJ, Richards AC, Mulvey MA. Invasion of Host Cells and Tissues by Uropathogenic Bacteria. Microbiol Spectr. 2016;4. doi:10.1128/microbiolspec.UTI-0026-2016

39. Berry RE, Klumpp DJ, Schaeffer AJ. Urothelial cultures support intracellular bacterial community formation by uropathogenic Escherichia coli. Infect Immun. 2009;77: 2762–72. doi:10.1128/IAI.00323-09

40. Pokrywczynska M, Czapiewska M, Jundzill A, Bodnar M, Balcerczyk D, Kloskowski T, et al. Optimization of porcine urothelial cell cultures: Best practices, recommendations, and threats. Cell Biol Int. 2016;40: 812–820. doi:10.1002/CBIN.10614

41. Berry RE, Klumpp DJ, Schaeffer AJ. Urothelial Cultures Support Intracellular Bacterial Community Formation by Uropathogenic Escherichia coli. Infect Immun. 2009;77: 2762. doi:10.1128/IAI.00323-09

42. Thumbikat P, Berry RE, Schaeffer AJ, Klumpp DJ. Differentiation-induced uroplakin III expression promotes urothelial cell death in response to uropathogenic E. coli. Microbes Infect. 2009;11: 57–65. doi:10.1016/j.micinf.2008.10.008

43. Wright KJ, Seed PC, Hultgren SJ. Development of intracellular bacterial communities of uropathogenic *Escherichia coli* depends on type 1 pili. Cell Microbiol. 2007;9: 2230–2241. doi:10.1111/j.1462-5822.2007.00952.x

44. Schwartz DJ, Kalas V, Pinkner JS, Chen SL, Spaulding CN, Dodson KW, et al. Positively selected FimH residues enhance virulence during urinary tract infection by altering FimH conformation. Proceedings of the National Academy of Sciences. 2013;110: 15530–15537. doi:10.1073/pnas.1315203110

45. Sokurenko E v., Chesnokova V, Dykhuizen DE, Ofek I, Wu X-R, Krogfelt KA, et al. Pathogenic adaptation of Escherichia coli by natural variation of the FimH adhesin. Proceedings of the National Academy of Sciences. 1998;95: 8922–8926. doi:10.1073/pnas.95.15.8922

46. Sharma K, Dhar N, Thacker V v, Simonet TM, Signorino-Gelo F, William Knott G, et al. Dynamic persistence of UPEC intracellular bacterial communities in a human bladder-chip model of urinary tract infection. Elife. 2021;10. doi:10.7554/ELIFE.66481

47. Daniels J, Tracy G, Irom S, Lakritz J. Fluoroquinolone levels in healthy dog urine following a 20-mg/kg oral dose of enrofloxacin exceed mutant prevention concentration targets against Escherichia coli isolated from canine urinary tract infections. J Vet Pharmacol Ther. 2014;37: 201–204. doi:10.1111/JVP.12069

48. Brown S, Arnold T, Hamlow P, Speedy A, Deleeuw N, Hubbard V, et al. Plasma and urine disposition and dose proportionality of ceftiofur and metabolites in dogs after subcutaneous administration of ceftiofur sodium. J Vet Pharmacol Ther. 1995;18: 363–369. doi:10.1111/J.1365-2885.1995.TB00605.X

49. Wilson B, Norris J, Malik R, Martin P, Wigney D, Baral R, et al. Susceptibility of bacteria from feline and canine urinary tract infections to doxycycline and tetracycline concentrations attained in urine four hours after oral dosage. Aust Vet J. 2006;84: 8–11. doi:10.1111/J.1751-0813.2006.TB13115.X
50. Thompson MF, Litster AL, Platell JL, Trott DJ. Canine bacterial urinary tract infections: New developments in old pathogens. The Veterinary Journal. 2011;190: 22–27. doi:10.1016/j.tvjl.2010.11.013
51. Hooton TM. Recurrent urinary tract infection in women. International Journal of Antimicrobial Agents. Elsevier; 2001. pp. 259–268. doi:10.1016/S0924-8579(00)00350-2
52. Glover M, Moreira CG, Sperandio V, Zimmern P. Recurrent urinary tract infections in healthy and nonpregnant women. Urological Science. Elsevier B.V.; 2014. pp. 1–8. doi:10.1016/j.urols.2013.11.007
53. Parajuli NP, Maharjan P, Parajuli H, Joshi G, Paudel D, Sayami S, et al. High rates of multidrug resistance among uropathogenic Escherichia coli in children and analyses of ESBL producers from Nepal. Antimicrob Resist Infect Control. 2017;6: 9. doi:10.1186/s13756-016-0168-6
54. Dehbanipour R, Rastaghi S, Sedighi M, Maleki N, Faghri J. High prevalence of multidrug-resistance uropathogenic Escherichia coli strains, Isfahan, Iran. J Nat Sci Biol Med. 2016;7: 22–6. doi:10.4103/0976-9668.175020
55. Hatt JK, Rather PN. Role of Bacterial Biofilms in Urinary Tract Infections. Springer, Berlin, Heidelberg; 2008. pp. 163–192. doi:10.1007/978-3-540-75418-3_8
56. Delcaru C, Alexandru I, Podgoreanu P, Grosu M, Stavropoulos E, Chifiriuc M, et al. Microbial Biofilms in Urinary Tract Infections and Prostatitis: Etiology, Pathogenicity, and Combating strategies. Pathogens. 2016;5: 65. doi:10.3390/pathogens5040065
57. Soto SM, Smithson A, Horcajada JP, Martinez JA, Mensa JP, Vila J. Implication of biofilm formation in the persistence of urinary tract infection caused by uropathogenic Escherichia coli. Clinical Microbiology and Infection. 2006;12: 1034–1036. doi:10.1111/j.1469-0691.2006.01543.x
58. Ejrnæs K, Stegger M, Reisner A, Ferry S, Monsen T, Holm SE, et al. Characteristics of Escherichia coli causing persistence or relapse of urinary tract infections: Phylogenetic groups, virulence factors and biofilm formation. http://dx.doi.org/104161/viru2618189. 2011;2: 528–537. doi:10.4161/VIRU.2.6.18189
59. Mulvey MA, Schilling JD, Hultgren SJ. Establishment of a persistent Escherichia coli reservoir during the acute phase of a bladder infection. Infect Immun. 2001;69: 4572–9. doi:10.1128/IAI.69.7.4572-4579.2001
60. Rosen DA, Pinkner JS, Jones JM, Walker JN, Clegg S, Hultgren SJ. Utilization of an Intracellular Bacterial Community Pathway in Klebsiella pneumoniae Urinary Tract Infection and the Effects of FimK on Type 1 Pilus Expression. Infect Immun. 2008;76: 3337–3345. doi:10.1128/IAI.00090-08
61. Schaffer JN, Norsworthy AN, Sun T-T, Pearson MM. Proteus mirabilis fimbriae- and urease-dependent clusters assemble in an extracellular niche to initiate bladder stone formation. Proc Natl Acad Sci U S A. 2016;113: 4494–9. doi:10.1073/pnas.1601720113
62. Nielubowicz GR, Mobley HLT. Host–pathogen interactions in urinary tract infection. Nat Rev Urol. 2010;7: 430–441. doi:10.1038/nrurol.2010.101
63. Conover MS, Hadjifrangiskou M, Palermo JJ, Hibbing ME, Dodson KW, Hultgren SJ. Metabolic requirements of Escherichia coli in intracellular bacterial communities during urinary tract infection pathogenesis. mBio. 2016;7: e00104-16. doi:10.1128/mBio.00104-16
64. Garofalo CK, Hooton TM, Martin SM, Stamm WE, Palermo JJ, Gordon JI, et al. Escherichia coli from urine of female patients with urinary tract infections is competent for intracellular bacterial community formation. Infect Immun. 2007;75: 52–60. doi:10.1128/IAI.01123-06

65. Sharma K, Dhar N, Thacker V v., Simonet TM, Signorino-Gelo F, Knott G, et al. Dynamic persistence of intracellular bacterial communities of uropathogenic escherichia coli in a human bladder-chip model of urinary tract infections. Elife. 2021;10. doi:10.7554/ELIFE.66481
66. Kloskowski T, Uzarska M, Gurtowska N, Olkowska J, Joachimiak R, Bajek A, et al. How to isolate urothelial cells? Comparison of four different methods and literature review. Hum Cell. 2014;27: 85–93. doi:10.1007/s13577-013-0070-y
67. Zhang Y, Atala A. Urothelial Cell Culture: Stratified Urothelial Sheet and Three-Dimensional Growth of Urothelial Structure. Humana Press, Totowa, NJ; 2012. pp. 383–399. doi:10.1007/978-1-62703-125-7_23
68. Sangha N. Isolation of Urothelial Cells from Bladder Tissue. Methods in molecular biology (Clifton, NJ). 2013. pp. 21–33. doi:10.1007/978-1-62703-363-3_3
69. Weissman SJ, Beskhlebnaya V, Chesnokova V, Chattopadhyay S, Stamm WE, Hooton TM, et al. Differential Stability and Trade-Off Effects of Pathoadaptive Mutations in the Escherichia coli FimH Adhesin. Infect Immun. 2007;75: 3548–3555. doi:10.1128/IAI.01963-06
70. Walters MC, Roe F, Bugnicourt A, Franklin MJ, Stewart PS, Stewart PS. Contributions of antibiotic penetration, oxygen limitation, and low metabolic activity to tolerance of Pseudomonas aeruginosa biofilms to ciprofloxacin and tobramycin. Antimicrob Agents Chemother. 2003;47: 317–23. doi:10.1128/AAC.47.1.317-323.2003
71. Stewart PS. Antimicrobial Tolerance in Biofilms. Microbial Biofilms, 2nd Edition. 2015;3: 269–285. doi:10.1128/microbiolspec.MB-0010-2014
72. Weese JS, Blondeau JM, Boothe D, Breitschwerdt EB, Guardabassi L, Hillier A, et al. Antimicrobial use guidelines for treatment of urinary tract disease in dogs and cats: antimicrobial guidelines working group of the international society for companion animal infectious diseases. Vet Med Int. 2011;2011: 263768. doi:10.4061/2011/263768
73. Weese JS, Blondeau J, Boothe D, Guardabassi LG, Gumley N, Papich M, et al. International Society for Companion Animal Infectious Diseases (ISCAID) guidelines for the diagnosis and management of bacterial urinary tract infections in dogs and cats. The Veterinary Journal. 2019;247: 8–25. doi:10.1016/J.TVJL.2019.02.008
74. Frazier DL, Thompson L, Trettien A, Evans EI. Comparison of fluoroquinolone pharmacokinetic parameters after treatment with marbofloxacin, enrofloxacin, and difloxacin in dogs. J Vet Pharmacol Ther. 2008;23: 293–302. doi:10.1111/j.1365-2885.2000.00285.x
75. Daniels JB, Tracy G, Irom SJ, Lakritz J. Fluoroquinolone levels in healthy dog urine following a 20-mg/kg oral dose of enrofloxacin exceed mutant prevention concentration targets against Escherichia coli isolated from canine urinary tract infections. J Vet Pharmacol Ther. 2014;37: 201–204. doi:10.1111/JVP.12069
76. Kumar V, Madabushi R, Lucchesi MBB, Derendorf H. Pharmacokinetics of cefpodoxime in plasma and subcutaneous fluid following oral administration of cefpodoxime proxetil in male beagle dogs. J Vet Pharmacol Ther. 2011;34: 130–135. doi:10.1111/J.1365-2885.2010.01198.X
77. Lawrenson RA, Logie JW. Antibiotic failure in the treatment of urinary tract infections in young women. Journal of Antimicrobial Chemotherapy. 2001;48: 895–901. doi:10.1093/jac/48.6.895
78. Murray BO, Flores C, Williams C, Flusberg DA, Marr EE, Kwiatkowska KM, et al. Recurrent Urinary Tract Infection: A Mystery in Search of Better Model Systems. Front Cell Infect Microbiol. 2021;11: 440. doi:10.3389/FCIMB.2021.691210/BIBTEX
79. Nielsen TK, Petersen NA, Stærk K, Grønnemose RB, Palarasah Y, Nielsen LF, et al. A Porcine Model for Urinary Tract Infection. Front Microbiol. 2019;10. doi:10.3389/FMICB.2019.02564
80. Barber AE, Norton JP, Wiles TJ, Mulvey MA. Strengths and Limitations of Model Systems for the Study of Urinary Tract Infections and Related Pathologies. Microbiol Mol Biol Rev. 2016;80: 351–367. doi:10.1128/MMBR.00067-15

81. Chockalingam A, Stewart S, Xu L, Gandhi A, Matta MK, Patel V, et al. Evaluation of Immunocompetent Urinary Tract Infected Balb/C Mouse Model For the Study of Antibiotic Resistance Development Using Escherichia Coli CFT073 Infection. Antibiotics 2019, Vol 8, Page 170. 2019;8: 170. doi:10.3390/ANTIBIOTICS8040170
82. Hannan TJ, Hunstad DA. A Murine Model for Escherichia coli Urinary Tract Infection. Methods Mol Biol. 2016;1333: 159–75. doi:10.1007/978-1-4939-2854-5_14
83. Hung C-S, Dodson KW, Hultgren SJ. A murine model of urinary tract infection. Nat Protoc. 2009;4: 1230–1243. doi:10.1038/nprot.2009.116
84. LeCuyer TE, Byrne BA, Daniels JB, Diaz-Campos D v, Hammac GK, Miller CB, et al. Population Structure and Antimicrobial Resistance of Canine Uropathogenic Escherichia coli. J Clin Microbiol. 2018;56. doi:10.1128/JCM.00788-18
85. CSLI. Performance Standards for Antimicrobial Susceptibility Testing; Twenty-Seventh Informational Supplement. CLSI Document M100-S27, Clinical and Laboratory Standards Institute, Wayne, Pa. 2017.
86. Thumbikat P, Berry RE, Zhou G, Billips BK, Yaggie RE, Zaichuk T, et al. Bacteria-Induced Uroplakin Signaling Mediates Bladder Response to Infection. Finlay BB, editor. PLoS Pathog. 2009;5: e1000415. doi:10.1371/journal.ppat.1000415
87. Cross WR, Eardley I, Leese HJ, Southgate J. A biomimetic tissue from cultured normal human urothelial cells: analysis of physiological function. American Journal of Physiology-Renal Physiology. 2005;289: F459–F468. doi:10.1152/ajprenal.00040.2005

Fig 1

Click here to access/download;Figure;Fig 1.tif

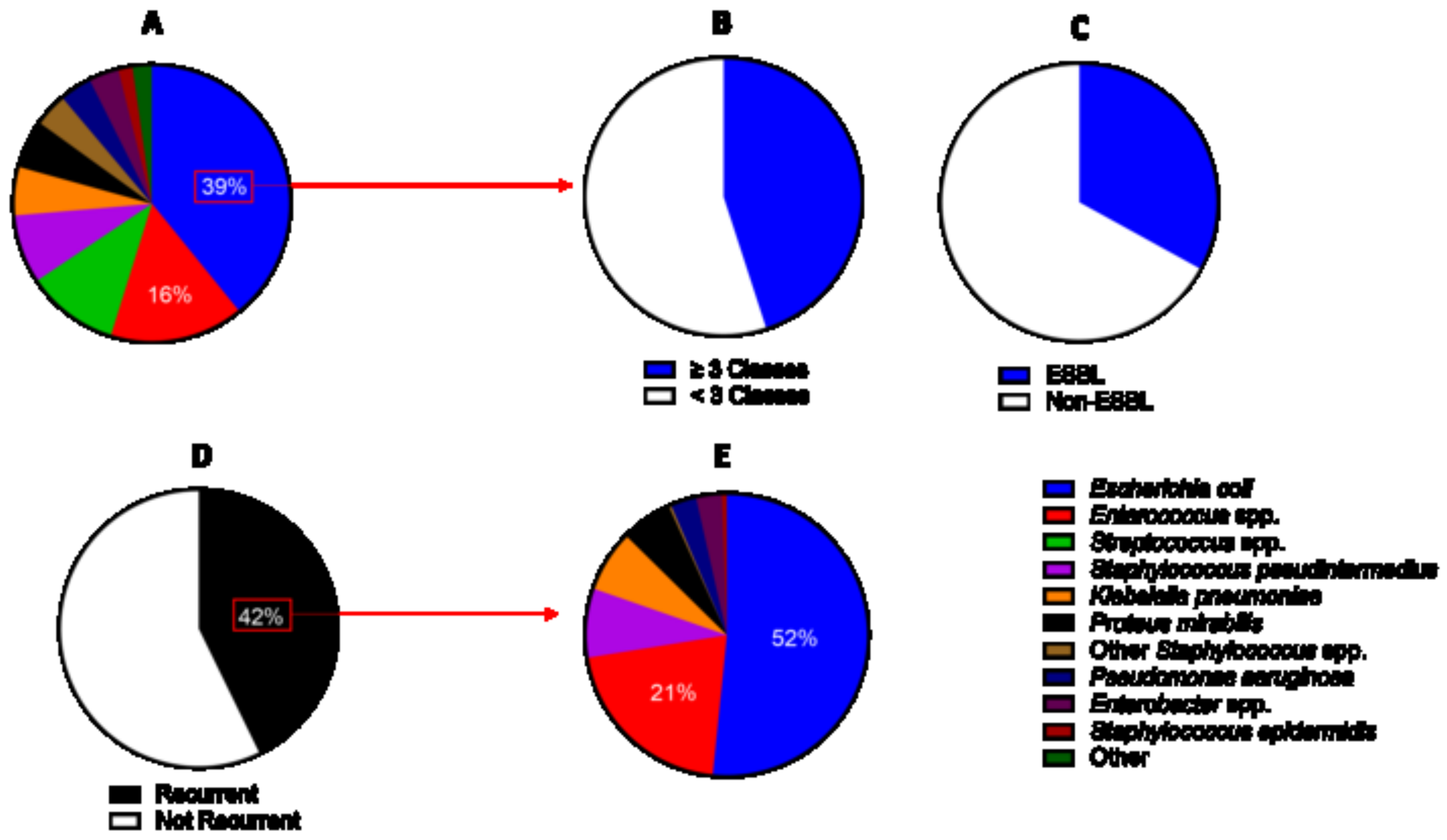

Fig 3

Click here to access/download;Figure;Fig 3.tif

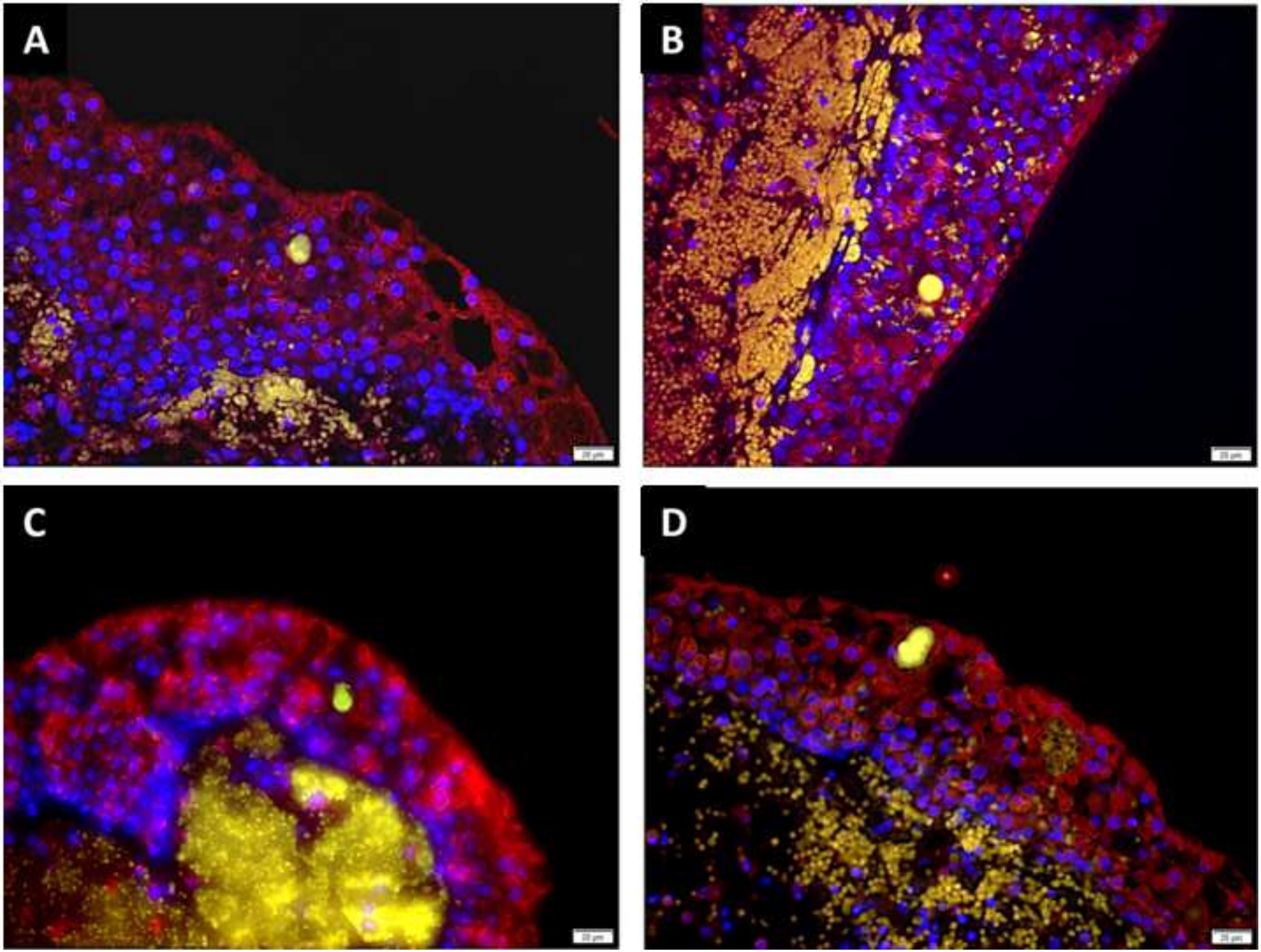

Click here to access/download;Figure;Fig 4.tif

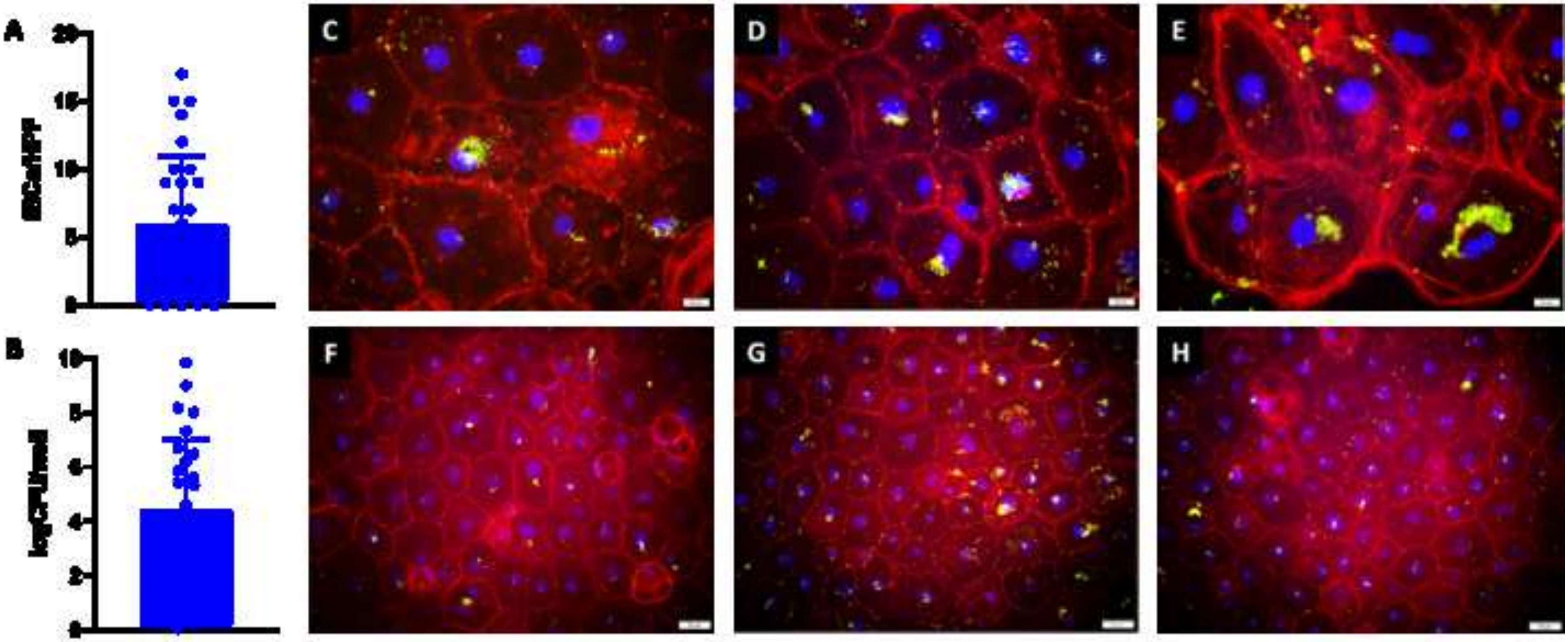

Fig 5 Click here to access/download;Figure;Fig 5.tif

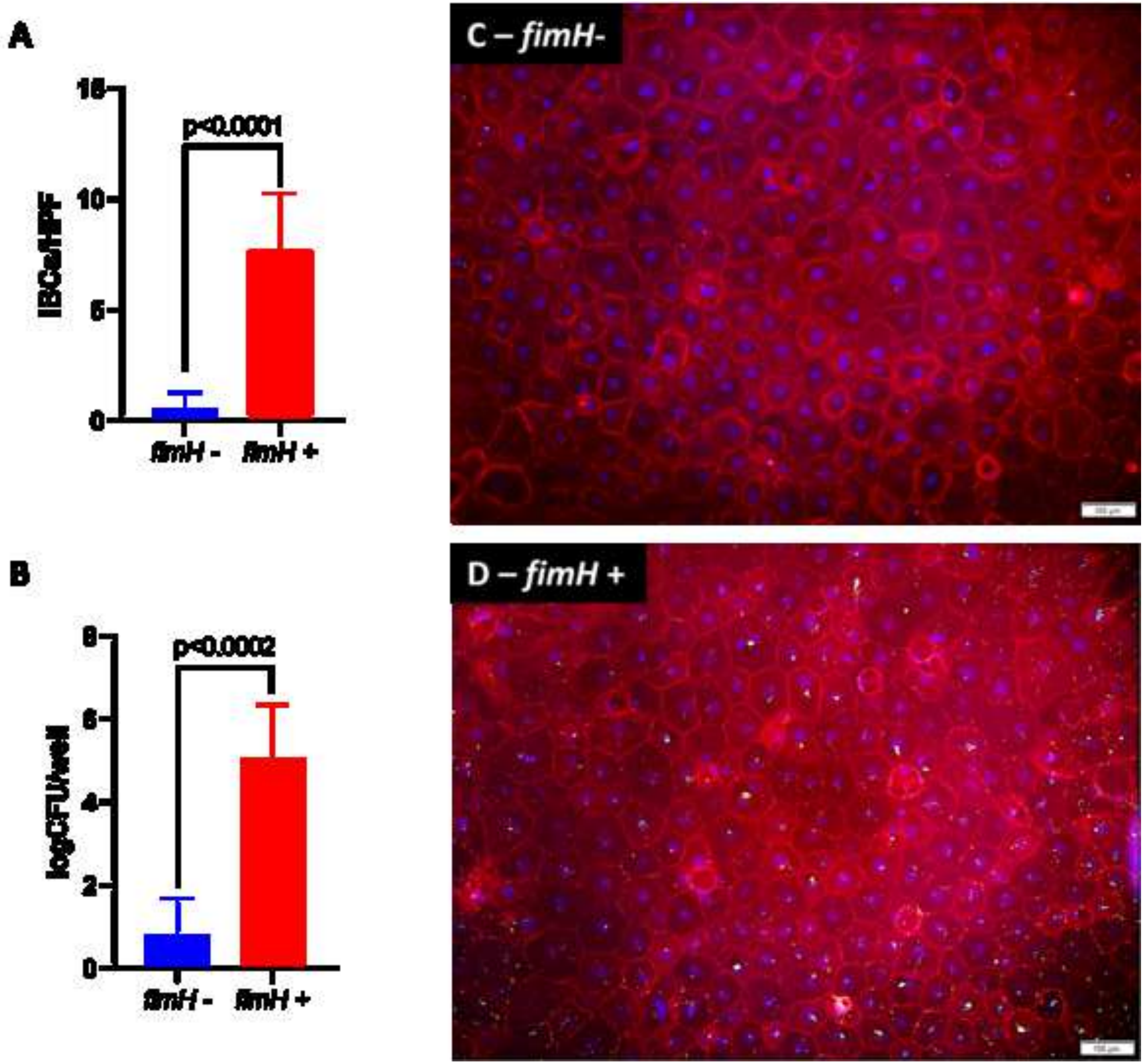

Click here to access/download;Figure;Fig 6.tif

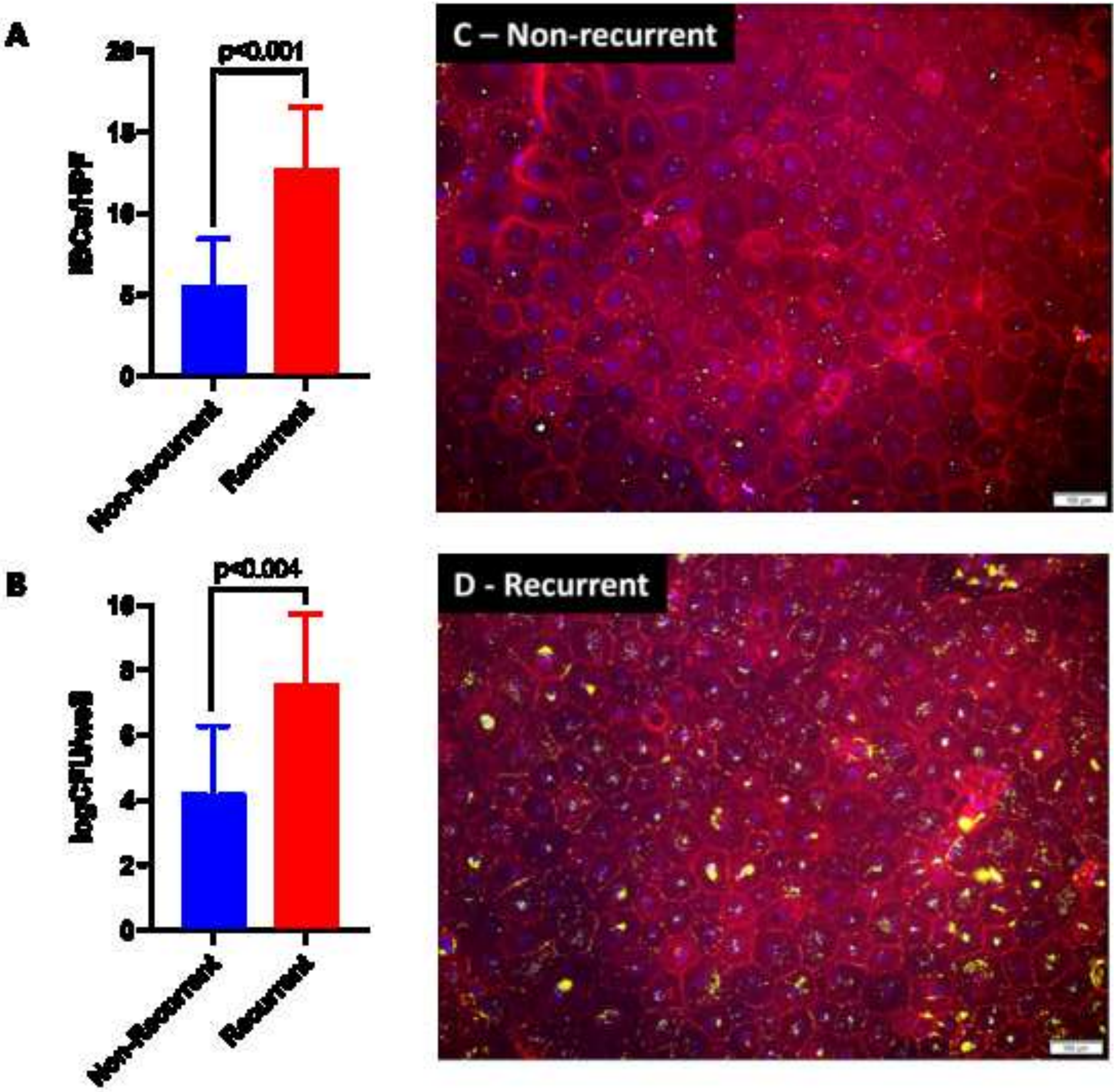

Fig 7 Click here to access/download;Figure;Fig 7.tif

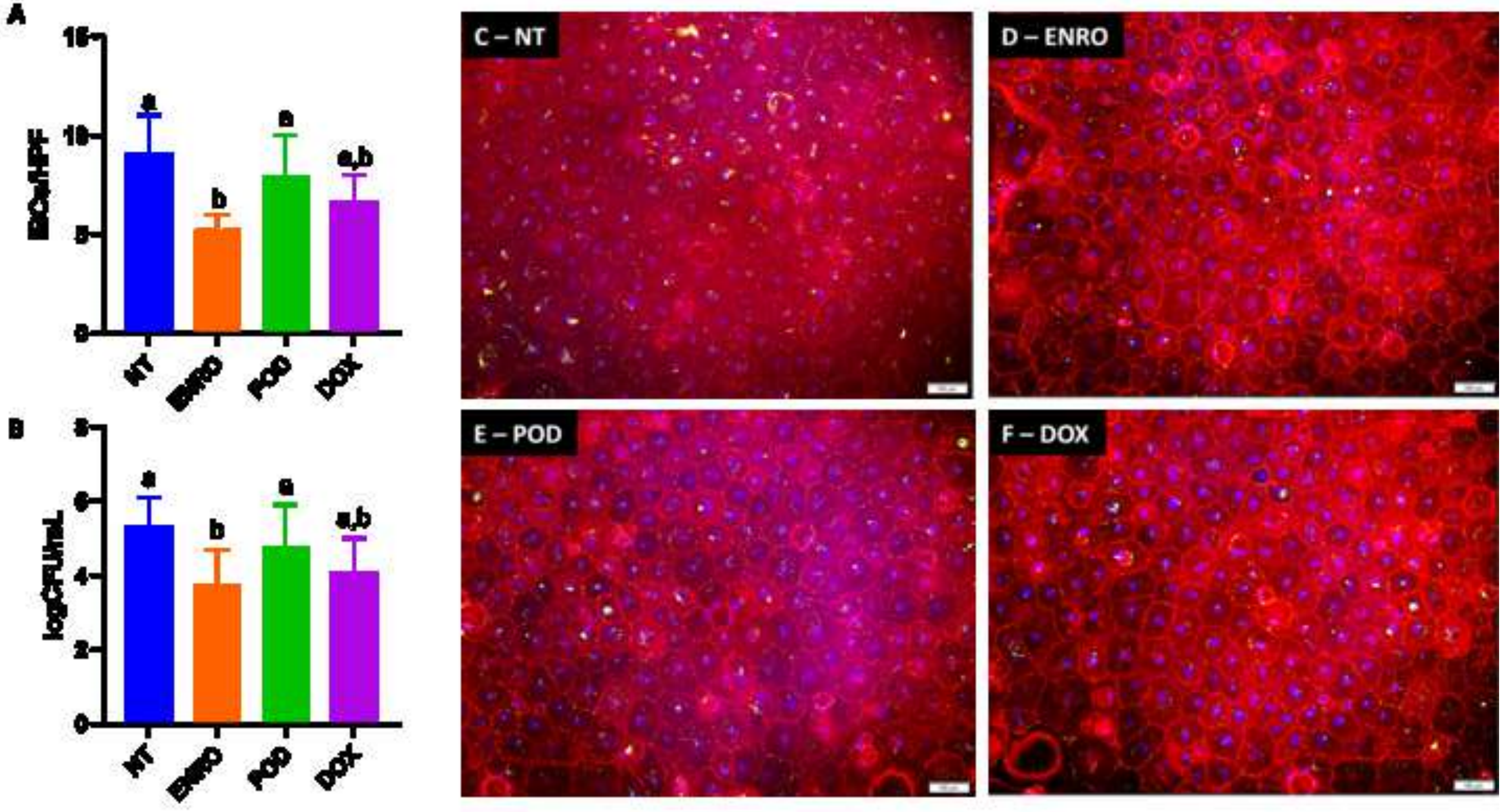

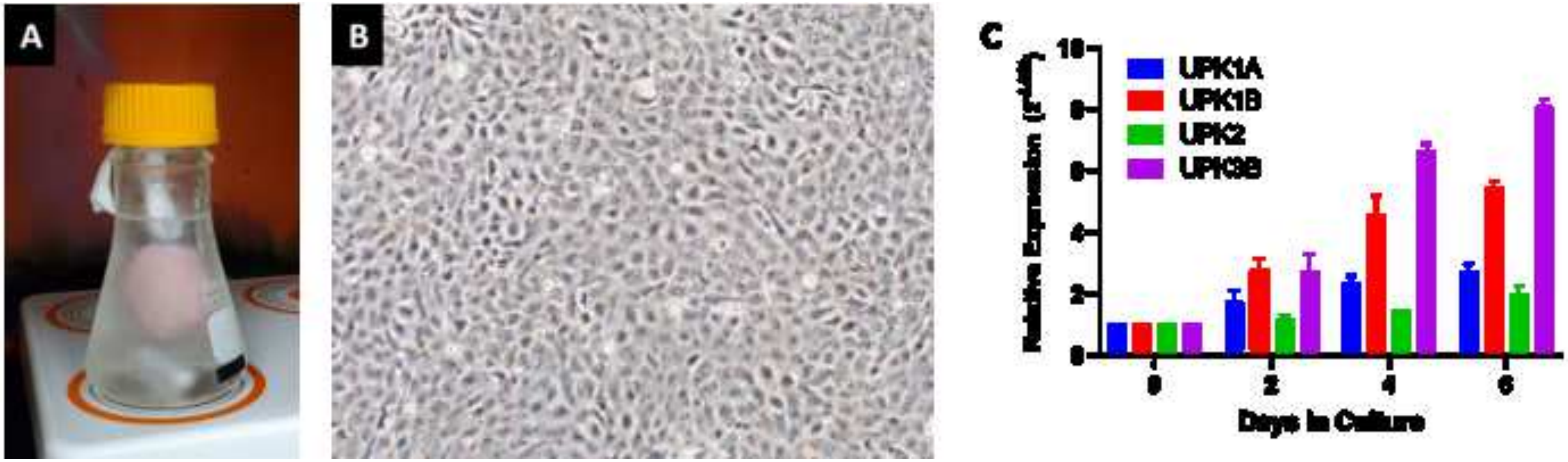
A
B
C
10
8
6
4
2
0
UPK1A
UPK1B
UPK2
UPK3B
Relative Expression
0
2
4
6
Days in Culture

Fig S2

Click here to access/download;Figure;Fig S2.tif

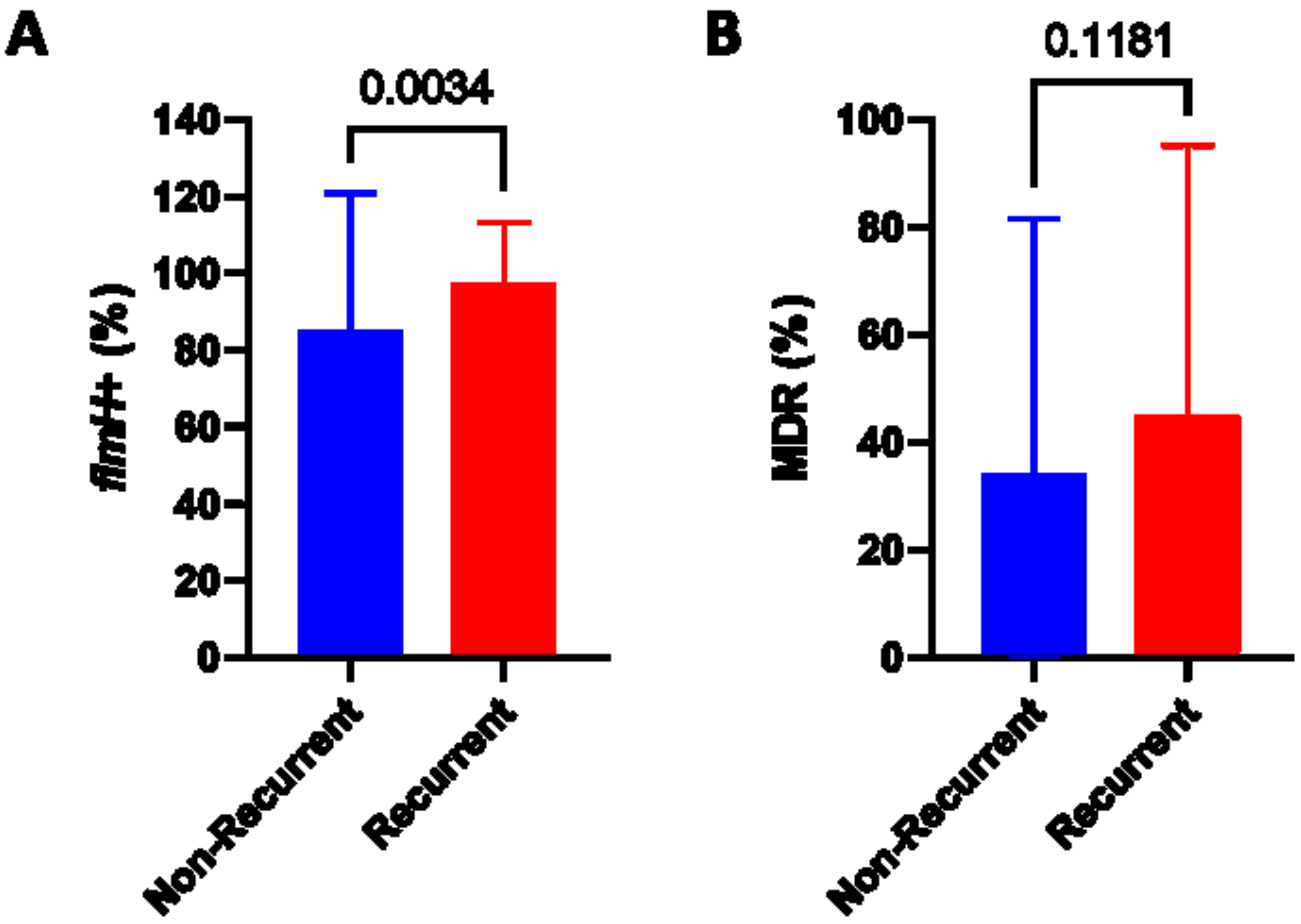

Fig 2

Click here to access/download;Figure;Fig 2 Revised.tif

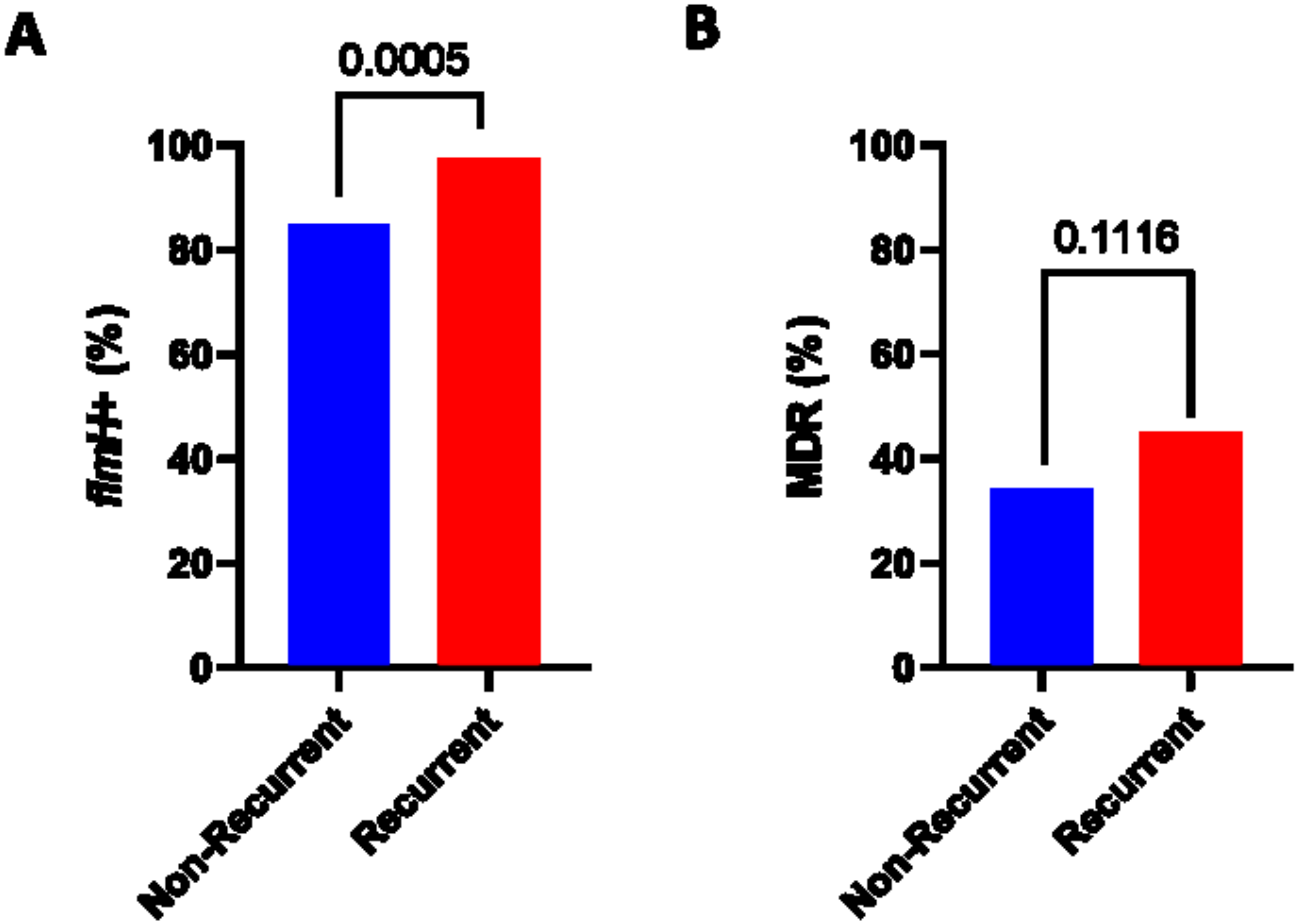

Canine urothelial cell model to study intracellular bacterial community development by uropathogenic *Escherichia coli*

Canine urothelial cells support intracellular *Escherichia coli*

Jessica M. Gilbertie[1,2], Breanna J. Sheahan[2,3], Shelly L. Vaden[3], Megan E. Jacob[1,2 *]

[1] Department of Population Health and Pathobiology, College of Veterinary Medicine, North Carolina State University, Raleigh, North Carolina, USA

[2]Comparative Medicine Institute, North Carolina State University, Raleigh, NC, USA

[3]Department of Clinical Sciences, College of Veterinary Medicine, North Carolina State University, Raleigh, North Carolina, USA

***Corresponding author:**

Email: mejacob@ncsu.edu

Abstract

Urinary tract infections (UTIs) are among the most common bacterial infections of both dogs and humans, with most caused by uropathogenic *Escherichia coli* (UPEC). Recurrent UPEC infections are a major concern in the treatment and management of UTIs in both ~~dogs and humans~~species. In humans, the ability of UPECs to form intracellular bacterial communities (IBCs) within urothelial cells has been implicated in recurrent UTIs. However, the role of IBCs has not been explored in the pathogenesis of canine recurrent UTIs. In this study, we identified IBCs in both urine and bladder tissue from dogs with UPEC associated UTIs. In addition, we

showed that UPECs derived from canine UTIs form IBCs within primary canine urothelial cells (5.8 IBCs/HPF and 4.3 log$_{10}$CFU/well). [illegible] in human UTIs, formation of IBCs by canine UPECs correlated with the presence of the *fimH* gene as those isolates lacking the *fimH* gene formed fewer IBCs in canine urothelial cells (0.5 IBCs/HPF and 0.8 log$_{10}$CFU/well) then those harboring carrying the *fimH* gene (7.7 IBCs/HPF and 5.1 log$_{10}$CFU/well). Additionally, UPEC strains from clinical cases classified as recurrent UTIs had higher rates of IBC formation (12.8 IBCs/HPF and 7.6 log$_{10}$CFU/well) than UPEC strains from non-recurrent UTIs. (5.5 IBCs/HPF and 4.2 log$_{10}$CFU/well). These IBCs were tolerant to treatment with enrofloxacin, cefpodoxime and doxycycline at 150, 50 and 50 µg/mL respectively, which are representative of the concentrations achieved in canine urine after standard dosing. This is consistent with the clinical perspective that current UTIs are a common condition of dogs and are difficult to manage through antimicrobial treatment. Additionally, the dog could prove to be a powerful model of IBC formation as they are natural mod[illegible]f UPEC-causing UTIs and have similar pathophysiology of IBC formation.

Introduction

Urinary tract infections (UTI) are among the most common bacterial infectioninfections of dogs accounting for 14% of medical cases. Uropathogenic *Escherichia coli* (UPEC) are the most common etiological agents in both human and canine UTIs[1,2]. Recurrent infections are a major concern in the treatment and management of UTIs of both species[3]. Previous reports show that 20% of dogs with UTIs will develop a recurrent infection with the same uropathogen, despite aggressive appropriate antimicrobial therapy, within 6-months of the original infection[4]. In humans, recurrent UTIs are associated with the ability of UPEC to form intracellular bacterial

Commented [GJ1]: Meg does this sound ok? I looked at Shelly's paper for a reference

communities (IBCs) that are tolerant to antimicrobial therapy[5–8]; however, the role of IBCs in canine UTIs has not been demonstrated.

There has been an increasing interest in the relationship between UPEC biofilm potential and infection persistence in recurrent UTIs; however, a previous study from our group was unable to identify associations between traditional biofilm formation *in vitro* and clinical recurrence of UTI in dogs[9]. The traditional definition of a biofilm is a community of bacteria within a polymeric matrix that is attached to an abiotic or biotic surface[10]. However, recent advancements in biofilm research suggest that, in the urinary tract, IBCs are considered non-surface attached, intracellular biofilms [11].

In human isolates, the ability of UPEC to adhere to and invade the urothelium to form IBCs is facilitated by the type 1 pilus-associated adhesin FimH[12]. The majority of UPECs encode the *fimH* gene, a part of the *fim* operon, which is transcribed to the FimH adhesion protein[13]. FimH binds to mannose-containing glycoprotein receptors on host cell membranes, which in the urinary tract, are the mannosylated residues on uroplakin proteins that line the apical surface of urothelial cells in the bladder[14]. UPEC utilize binding of FimH to the uroplakin receptor complex to enable adhesion to the urothelial surface[12]. Subsequently, the binding of FimH to uroplakin triggers host cell signaling promoting bacterial internalization[15]. Thereafter, UPEC grow and divide intracellularly forming small clusters of bacteria termed IBCs[16]. Once an IBC develops, UPEC are protected, able to tolerate the -cidal activity of many antimicrobials[6,17].

Dogs are an ideal translational model for urinary tract infection research as . tTtheirheir size allows for repeated access to large volumes of urine using minimally invasive techniques[18] and . Additionally, biopsies of bladder tissue can be easily accomplished via uroscopy. In addition, Ddogs are commonly used as models of human disease and are great pharmacokinetic models for

novel therapeutics[19–21]. In additionAlso, dogs have similar urinary tract infectionUTI pathogenesisepidemiology , are afflicted by the same types of bacteria, and have urinary biology comparable to humans[2,4,22,23]. FFor UPEC specific UTIs, dogs are infected by similar sequence types (STs), have similar rates of multi-drug resistant strains, and canine *E. coli* isolates have shown similar virulence traits and biofilm phenotypes as human isolates [24–26]. However, it is currently unknown if canine UTIs have a similar pathogenesis to human UTIs. More specifically if IBCs are part of UTI pathogenesis in canines.

Commented [GJ2]: Meg changed this to epidemiology -- does that work since we actually don't know if the pathogenesis is similar

The Therefore, the goal of this study was to determine if dogs demonstrate similar UPEC associated UTI pathogenesis predominated by IBC formation as observed in humans and murine models[27]. Through retrospective data analysis, we demonstrated that dogs are clinically afflicted by similar uropathogens with similar resistance rates and recurrent infection frequencies as human UTIs. In clinical specimens, we demonstrated that UPEC-causing UTIs were associated with IBCs on both urine cytologies and bladder biopsies in symptomatic dogs; additionally, we validated an *in vitro* IBC model using primary canine urothelial cells. Using this *in vitro* IBC model, we validated the role of *fimH* in IBC formation in urothelial cells, assessed IBCs tolerance to treatment with several classes of clinically relevant antimicrobials, and compared IBCs *in vitro* from UPEC isolated from recurrent UTI cases compared to UPEC isolated from nonrecurrent UTI cases.

Results

Canine UTIs exhibit similar uropathogen distribution, antimicrobial resistance and recurrent infection frequencies as human UTIs

To compare the epidemiology of canine and human UTIs we performed a retrospective analysis of canine uropathogens collected at a veterinary teaching hospital over a 5-year period (2015-2019). During that time, 797 canine urine isolates were obtained, of which 87.3% were monospecies cultures and 12.7% were polymicrobial. The most common UTI associated pathogen was *Escherichia coli* (39.1% of positive cultures), followed by *Enterococcus* spp. (15.7%), *Streptococcus* spp. (10.7%), *Staphylococcus pseudintermedius* (8%), *Klebsiella pneumoniae* (5.6%), and *Proteus mirabilis* (5.6%) (Fig 1A). In humans, *E. coli* is the ~~most common pathogen causing UTIs~~dominant uropathogen as well; however, occurrence is higher with reported rates from 65-75%[28,29]. The second most common UTI associated pathogen of humans varies by study and are similar to canine UTI associated pathogens including *Staphylococcus saprophyticus*, *K. pneumoniae*, *P. mirabilis* and *Enterococcus* spp.[30]. Of the polymicrobial infections in our analysis, the most commonly isolated combination was *Escherichia coli* and *Enterococcus* spp. (typically *faecium* and *faecalis*) (24.5%). The *E. coli-Enterococcus* spp. combination is also seen in approximately 36% of human polymicrobial UTIs[31]. The sex distribution of affected dogs was 60.9% female (female intact and female spayed) and 39.1% male (male intact and male castrated) and the median age at presentation was 5.6 years (middle aged adult). Similarly, in human medicine a higher rate of UTIs is observed in women and the highest UTIs rates are seen patients from 18-65 years of age[32].

[illegible]e *Escherichia coli* isolates obtained from either mono- or polymicrobial canine UTIs, 45% were defined as multidrug resistant (MDR; resistant to ≥ 3 antimicrobial classes) (Fig 1B) and 33% demonstrated resistance to beta-lactam antimicrobials based on the presence of *blaCMY* and/or *blaTEM* genes (Fig 1C). In the human literature, 49-68% of UPEC are classified as MDR and 24-40% were extended spectrum beta-lactamase (ESBL) producers[34–36].

Canine patients diagnosed with a recurrent UTI, defined as more than 1 positive urine culture within 6 months of the first urine culture and with the same uropathogen despite antimicrobial therapy, occurred in 42% of cases (Fig 1D). Comparable recurrence rates of 27-53% are observed in human UTIs [28,32]. In parallel to human studies, the most common bacterial pathogens causing recurrent UTIs in our retrospective analysis were *Escherichia coli* (51.6%) and *Enterococcus* spp. (20.8%) (Fig 1E)[33].

Fig 1. ~~**Incidence**~~ **Frequency of various uropathogens in canine urinary tract infections**. ~~A retrospective analysis of positive urine cultures submitted to the Clinical Microbiology Laboratory at the North Carolina State University College of Veterinary Medicine were reviewed over a 5-year period (2015-2019).~~ (A) Pathogen ~~incidence~~ ~~occurence~~occurrence of all positive urine cultures (n=797). Corresponding medical records were reviewed to determine if the canine patient was experiencing a recurrent UTI defined by more than one positive culture of the same uropathogen within 6 months of one another. (B) ~~Incidence~~~~Occurence~~Occurrence of multidrug resistance in the *Escherichia coli* population (n=312) ~~only~~ defined by resistance to greater than or equal to three antimicrobial drug classes. ~~Recurrence rates observed over the 5-year period of the retrospective~~ (C) ~~Pathogen incidence or urine cultures from a recurrent infection.~~ Antimicrobial susceptibility profiles and gene ~~expression~~ presence of common~~for several~~ ESBL genes~~, which are standard characterization parameters within~~ ~~(standard screening with~~ the Clinical Microbiology Laboratory for any *Escherichia coli*~~)~~ were reviewed. ~~Incidence~~~~Occurence~~Occurrence of at least 1 positive ESBL gene in the *Escherichia coli* population only. (D) Recurrence rates observed over the 5-year period of the retrospective ~~Incidence of multidrug resistance in the *Escherichia coli* population (n=312) only defined by resistance to greater than or equal to three antimicrobial drug classes.~~ (E) Pathogen ~~incidence~~presence or urine

cultures from a recurrent infection. Incidence of at least 1 positive ESBL gene in the *Escherichia coli* population only.

IBCs and filamentous bacteria are observed on urine cytologies and bladder biopsies from dogs diagnosed with UPEC associated UTIs

During human and murine-model UPEC pathogenesis, the bacteria bind to and invade into the cells of the urothelium where they replicate and form IBCs[11,16,37]. After IBCs mature, they will disperse out of the cell to infect neighboring facet cells[38]. When the bacteria emerge from the urothelial cell, they adopt a filamentous phenotype. Both IBCs and filamentous bacteria have been identified in the urine of humans with UTIs[27]. To determine if dogs with naturally occurring UTIs exhibit the presence of IBCs and filamentous bacteria, we evaluated urine cytologies (n=30) and bladder biopsies (n=10) from dogs with UPEC associated UTIs. Immunofluorescence was used to identify IBCs and filamentous bacteria (Fig 2). In our analysis of urine cytologies by immunofluorescence, we found UPEC IBCs in 60% of the cytologies evaluated (Fig 2A; 18 of 30) and observed filamentous bacteria in 77% of the cytologies evaluated (Fig 2B; 23 of 30). Specifically, IBCs (anti-*E. coli*, green) were appreciated within sheets of urothelial cells (anti-uroplakin III, purple) (Fig 2CD) and individual urothelial cells (Fig 2EF). We also identified filamentous bacteria dispersing from the urothelium-associated IBCs (Fig 2GH). Upon evaluation of the bladder biopsies, we observed IBCs (anti-*E. coli*, green) within the urothelium (anti-Ecadherin, red) in 4 out of the 10 dogs evaluated (Fig 3). The morphology of the IBCs observed in these clinical canine samples reflect those seen in humans and mice[21].

Fig 2. Canine urine cytologies show evidence of IBCs and filamentous bacteria. Urine cytologies were evaluated from dogs exhibitingfrom dogs with clinical symptoms of a UTI and a positive *Escherichia coli* culture (n=30). The cytologies were made by the Clinical Pathology

~~Laboratory at North Carolina State University's College of Veterinary Medicine according to their standard operating procedures. The cytologies were then fixed, permeabilized, blocked and~~) were stained with an anti-Escherichia coli FITC (green) antibody, an anti-uroplakin IIIB antibody with an AlexaFluor 647 secondary antibody (purple) ~~(Abcam, Cambridge, UK)~~ and DAPI (blue) to stain nuclei. (A) The number of urine cytologies evaluated that had observable IBCs within shed urothelial cells. (B) The number of urine cytologies evaluated that had observable filamentous bacteria. (C,D) Lower magnification of large urothelial cell sheets containing both IBCs and filamentous bacteria. (E,F) Higher magnification with white arrows pointing to discrete IBCS within single shed urothelial cells. (G,H) Higher magnification with white arrows pointing to filamentous bacteria emerging from the urothelium.

Fig 3. IBCs are observed within the urothelium of dogs with UPEC-causing UTIs. (A-D) Representative images of IBCs noted within the bladder epithelium of four different canine patients. ~~Briefly, bladder biopsies of dogs presenting to the Internal Medicine Service at North Carolina State University's College of Veterinary Medicine (NCSU CVM) were evaluated. Biopsies~~ Bladder biopsies were only evaluated from dogs with clinical signs of a UTI and a positive UPEC culture. ~~Paraffin-embedded sections~~Tissue samples were stained ~~underwent heat induced antigen retrieval followed by staining~~ with the following antibodies: E-cadherin with an AlexaFluor 555 secondary (red), Escherichia coli-FITC (green) and DAPI to stain nuclei (blue).

Canine urothelial cell cultures support the formation of IBCs by clinical UPEC isolates

Previous research has shown that an immortalized human bladder epithelial cell line (PD07i) supports the growth of IBCs by UPEC *in vitro*[39]. Our aim was to develop an *in vitro* canine UTI model using canine primary urothelial cells and clinical canine UPEC strains.

Urothelial cells were harvested and cultured from canine bladders collected post-mortem from ~~healthy~~ dogs without signs of urinary or kidney disease as previously described (Fig S1)[40]. Primary canine urothelial cells (n=3) were then infected with UPEC strains originally isolated from~~from~~ symptomatic canines with confirmed UTIs (n=30). IBCs were observed 24 hours post-infection (anti-*E. coli*, green) within cultured urothelial cells (F-actin, phalloidin, red) (Fig 4). Specifically, we observed several of the UPEC strains forming IBCs within our canine urothelial cell model with an average of 5.8±5.15 IBCs observed per high powered field (HPF) (Fig 4A) and an average of 4.3±2.63 log_{10}CFU per well (Fig 4B). Five of the 30 (16.7%) UPEC isolates evaluated had no observable IBCs, correlating with <1 log_{10}CFU/mL (Fig 4AB). The IBCs of the canine clinical UPEC strains grown in canine primary urothelial cells exhibited similar morphology to the IBCs of human UPEC strains grown in an immortalized bladder cell line (Fig 4C-H)[41,42].

Fig S1. Canine urothelial cell isolation and differentiation. Canine bladders (n=10) were aseptically collected postmortem from apparently healthy dogs after planned euthanasia by an animal control facility. (A) The bladder was removed, turned ~~by severing of the ureters and ligaments and excising the bladder at the neck. The bladder was turned~~ inside out to expose the mucosa and~~. The neck of the bladder was sutured closed to ensure only mucosa was exposed. (A) The tissue was then~~ suspended in an Erlenmeyer flask ~~in 200mL HBSS containing antibiotic-antimycotic and 2.4U/mL Dispase II and incubated for 2 hours at 5% CO2, 90% humidity, and 37°C~~ under constant rotation. The tissue was treated with Dispase to remove the urothelial cells and cells were subsequently ~~After 2 hours, any remaining tissue on the mucosal surface was gently scraped off. The cell were pelleted by centrifugation, washed and resuspended in fresh HBSS. Cells were then~~ seeded onto collagen coated flasks ~~at 40,000 cells/cm2 in UCPM. The culture~~

medium was changed every 48 hours forand grown in culture 5-7 days. UCPM is a serum-free media and was used to proliferate urothelial cells while decreasing fibroblast contamination. (B) By 7 days a pure urothelial cell culture was appreciated by brightfield microscopy (10x) and analysis of cellular morphology as described by others[37,63,65]. Urothelial cells were frozen in aliquots and stored in liquid nitrogen until future use. (C) Canine urothelial cells were thawed, and seeded collagen coated 24-well tissue culture plates in UCPM and grown. Cells were grown for 72 hours, or until confluency. Thereafter, the culture media was changed to promote urothelial cell differentiation for expression of uroplakins required for UPEC pathogenesis (termed UCDM) as described elsewhere[39,83,84]. Cells were cultured in UCDM for 48 hours, followed by a media change, and then cultured for an additional 48 hours. Expression of various uroplakins was determined by real-time polymerase chain reaction using canine specific primers and the housekeeping gene GAPDH. Relative expression compared to the day 0 (before changing to UPDM) is presented at $2^{-\Delta\Delta Ct}$.

Fig 4. Canine primary urothelial cells support IBC formation by canine origin associated UPECs. Urothelial cells isolated from healthy canine bladders (n=3) were infected with clinical UPEC isolates from cases of canine symptomaticclinical canine UTIs (n=30) in triplicate for each canine donor . Two hours after infection, gentamicin was added to kill any remaining extracellular bacteria. The infected cultures were then incubated for another 22 hours. Twenty-four hours post-infection, the cultures were washed gently with warm PBS and fixed with paraformaldehyde. After fixationInfected cells stained, the cells were permeabilized and blocked prior to staining with an anti-Escherichia coli FITC antibody (green). Cells were also stained), with phallodin (red) to stain actin and DAPI (blue) to stain nuclei. (A) The number of IBC per high powered field (HPF) were counted for each donor/UPEC combination. The graph represents

the average number of IBCs for each UPEC isolate counted in each donor and in each replicate. (B) The bacterial load per well was measured by lysing the cells in a second culture that was unfixed to release the intracellular bacteria. ~~Each well was serial diluted and plate counted for colony forming units (CFUs).~~ Data is represented as a log CFU per well. (C-E) High-powered representative images (20x) of IBCs observed in infected canine urothelial cells with various canine origin UPEC stains. (F-H) Low-powered representative images (10x).

IBC formation in canine urothelial cell culture is dependent upon *fimH*

Previous research in humans has shown that IBC formation is dependent upon the presence and expression of type 1 pilus or ~~fimH~~ FimH in UPEC[12,43]. Therefore, we infected canine urothelial cells from three donors (n=3) with clinical canine UPEC strains that were *fimH* positive (n=9) or negative (n=9) to determine the association with IBC formation. We found that clinical UPEC isolates containing the *fimH* gene formed significantly more IBCs in canine urothelial cells (7.7 IBCs/HPF) as compared to isolates lacking the *fimH* gene (0.5 IBCs/HPF) ~~as compared~~ (Fig 5A, p<0.0001). The presence of ~~F~~*fimH* ~~status~~ also affected the ~~bacterial *load*~~*E. coli concentration* within urothelial infections, with *fimH* positive isolates establishing a higher CFU/well (5.1 log$_{10}$CFU/well) as compared to *fimH* negative isolates (0.8 log$_{10}$CFU/well) (Fig 5B, p<0.0002). Representative images of urothelial infections from a *fimH*- UPEC infection (Fig 5C) and *fimH*+ UPEC infection (Fig 5D) further ~~demonstrate~~ suggest that canine origin UPEC stains lacking *fimH* are unable to establish robust IBCs in canine urothelial cells which correlates with results seen in the murine model[12,44,45].

Fig 5. Canine UPEC isolates ~~lacking *fimH* do not form robust IBCs in comparison to isolates that harbor~~with ~~an intact~~ with the *fimH* gene form more robust IBCs compared to

Formatted: Font: Not Italic

Formatted: Font: Italic

isolates lacking *fim*H. Urothelial cells from canine donors (n=3) were isolated, cultured, infected and evaluated as in Fig 4. Specifically, canine urothelial cells were infected with canine UPEC isolates lacking the *fimH* gene (n=9) or those that harbored the *fimH* gene (n=9). ~~The presence or absence of the *fimH* gene was confirmed by PCR as previously described [23].~~ (A) The number of IBCs per high powered field (HPF) were counted for each donor/UPEC combination. The graph represents the average number of IBCs for each UPEC isolate counted in each donor and in each replicate. (B) The bacterial ~~load~~ concentration per well was measured by lysing the cells in a second culture that was unfixed to release the intracellular bacteria. ~~Each well was serial diluted and plate counted for colony forming units (CFUs).~~ Data is represented as a log CFU/~~per~~ well. (C) Representative images of a urothelial cell infection by a *fimH*- UPEC. (D) Representative images of a urothelial cell infection by a *fimH*+ UPEC. Bars are means and standard deviations and significant differences (p<0.05) were determined by an unpaired, nonparametric t-test (for IBCs/HPF) and parametric t-test (for log CFU/mL).

Canine urothelial cell IBC formation is more robust in UPEC isolates derived from cases of recurrent UTIs

To date, the genotypic or phenotypic relationship between UPEC strains and recurrent UTIs is unknown. Due to the variability in clinical UPEC isolates ability to form IBCs, we hypothesized that UPEC strains from recurrent UTI cases would form more IBCs *in vitro*. Above we showed that IBC formation was reliant upon the presence of *fimH*. ~~We also tested for the presence of the *fimH* gene within this group.~~ Interestingly, UPEC isolates from recurrent UTIs ~~expressed~~ were positive more frequently for *fimH* (97.5%) ~~versus~~ than UPEC isolates from non-recurrent cases (84.9%) (p<0.004; Fig S2A). Therefore, in this experiment we only used UPEC isolates that were *fimH* positive. W~~to compare IBC formation between UPEC isolates from~~

Commented [GJ3]: Meg -- I swapped the order in which I presented the fimH data and recurrent data so hopefully its clearer now. Let me know if not and I can edit more.

~~recurrent and non-recurrent cases. Therefore, w~~e evaluated *fimH+* UPEC strains from recurrent canine cases (n=12) of symptomatic UTIs treated with standard ~~appropriate~~ antimicrobial protocols with at least once recurrence within 6 months of the first UTI~~. We compared these recurrent UPEC strains with~~ ~~versus~~ and compared to matched *fimH+* UPEC strains from non-recurrent cases of symptomatic UTIs. The non-recurrent cases all had well documented follow-up to ensure a second UPEC-associated UTI did not occur within 6 months of the initial UTI. ~~We also tested for the presence of the *fimH* gene within this group. Interestingly, UPEC isolates from recurrent UTIs expressed more *fimH* (97.5%) versus UPEC isolates from non-recurrent cases (84.9%) (Fig S2A). Therefore, we only used UPEC isolates that were *fimH* positive to compare IBC formation between UPEC isolates from recurrent and non-recurrent cases. We observed a significant difference in both number of IBCs and the size of the IBCs from recurrent UPEC strains.~~ UPEC strains from recurrent cases formed more IBCs (12.8 IBCs/HPF) as compared to UPEC from non-recurrent cases (5.5 IBCs/HPF) (Fig 6A, $p<0.001$). UPEC strains from recurrent cases also had a higher intracellular bacterial ~~load per well~~concentration (7.6 log_{10}CFU/well) then UPEC from non-recurrent cases (4.2 log_{10}CFU/well) (Fig 6B, $p<0.004$). Representative images of urothelial infections show that UPECs from non-recurrent cases (Fig 6C) form less IBCs then UPECs from recurrent cases (Fig 6D). As all the UPEC strains from recurrent and non-recurrent cases were confirmed *fimH+*, further investigation into the mechanisms behind the increased ability of recurrent UPEC strains to form robust IBCs is warranted as *fimH* is not the only gene involved in IBC formation.

Fig S2. Canine UPEC isolates from recurrent UTIs correlate with *fimH* gene ~~expression~~ presence but not multidrug resistance (MDR). Canine UPEC isolates (n=-206) were classified as recurrent with well-documented recurrence of the same phenotypic strain within 6

months of the initial UTI. (A) Each isolate was evaluated for the presence or absence of the *fimH* gene ~~was confirmed~~ by PCR ~~as previously described~~ [23]. (B) Incidence of multidrug resistance was defined by resistance to greater than or equal to three antimicrobial drug classes. Bars are means and standard deviations and significant differences ($p<0.05$) were determined by an unpaired, nonparametric t-test.

Fig 6. Canine UPEC isolates from patients with recurrent UTIs form more robust IBCs then isolates from patients with non-recurrent UTIs. Urothelial cells from canine donors (n=3) were isolated, cultured, infected, and evaluated as shown in Fig 4. Specifically, canine urothelial cells were infected with canine *fimH+* UPEC isolates from cases with a well-documented recurrence of the same strain within 6 months of the initial UTI (n=12). We matched these UPEC isolates with ~~*those*~~ *fimH+* isolates from non-recurrent cases with well document follow-up in their medical records to ensure that a second UPEC-causing UTI did not occur within 6 months of the initial episode (n=12). (A) The number of IBCs per high powered field (HPF) were counted for each donor/UPEC combination. The graph represents the average number of IBCs for each UPEC isolate counted in each donor and in each replicate. (B) The bacterial ~~load per~~concentration ~~well~~ was measured by lysing the cells in a second culture that was unfixed to release the intracellular bacteria. ~~Each well was serial diluted and plate counted for colony forming units (CFUs).~~ Data is represented as a log CFU per well. (C) Representative images of a urothelial cell infection by a non-recurrent UPEC isolate. (D) Representative images of a urothelial cell infection by recurrent UPEC isolate. Bars are means and standard deviations and significant differences ($p<0.05$) were determined by an unpaired, nonparametric t-test (for IBCs/HPF) and parametric t-test (for $\log_{10}$CFU/mL).

Formatted: Font: Italic

IBCs in canine urothelial cell culture are tolerant to antimicrobial treatment

Both *in vitro* and *in vivo* studies have shown that UPEC IBCs persist intracellularly despite treatment with antimicrobials[6,46]. In the clinical setting, dogs with recurrent UTIs typically receive first-line antimicrobials, which despite demonstrating *in vitro* susceptibility, may result in persistent infections, and the dog will remain culture-positive. Therefore, we wished to investigate the antimicrobial tolerance of IBC-forming UPECs to antimicrobials commonly used to treat canine UTIs. We infected canine primary urothelial cells from three separate donors (n=3) with three UPEC isolates (n=3) known to form robust IBCs and allowed IBCs to develop for 24 hours. Subsequently, we treated with infected urothelial cells cultures with enrofloxacin (ENRO, 150µg/mL), cefpodoxime (POD, 100 µg/mL) and doxycycline (DOX, 50µgmL) at the Cmax observed in urine in canine pharmacokinetic studies[47–49]. All the UPEC clinical isolates used for the urothelial infections were susceptible to these antimicrobials as determined by *in vitro* susceptibility testing (data not shown). We found that no antimicrobial treatment tested could eradicate IBCs considerably at concentrations at least 10x the minimum inhibitory concentration (MIC), (No Treatment (NT) 9.1 IBCs/HPF versus enrofloxacin (ENRO) 5.2 IBCs/HPF, cefpodoxime (POD) 7.9 IBCs/HPF, and doxycycline (DOX) 6.7 IBCs/HPF) (Fig 7A). Additionally, we did not appreciate a greater than 2 log reduction in bacterial concentration~~load~~ (NT 5.3 log$_{10}$CFU/well versus ENRO 3.8 log$_{10}$CFU/well, POD 4.7 log$_{10}$CFU/well, and DOX 4.1 log$_{10}$CFU/well, respectively) (Fig 7B). Representative images showing the presence of IBCs (Fig 7C-F) despite antimicrobial treatment ~~at well~~ above the MIC. These results show that, similar to traditional biofilms, IBCs grown in canine urothelial cells are tolerant to antimicrobials and therefore are likely to persist intracellularly in the face of antimicrobial treatment.

Fig 7. IBCs formed in canine urothelial cells by ~~canine-origin~~ canine-sourced UPECs are tolerant to treatment with several antimicrobials from different classes. Urothelial cells from canine donors (n=3) were isolated, cultured, infected and evaluated as in Fig 4. Specifically, canine urothelial cells were infected with canine UPEC isolates known to form robust IBCs based those in Fig 4 (n=3) and susceptible to the antimicrobials tested herein. (A) The number of IBCs per high powered field (HPF) were counted for each donor/UPEC/treatment combination. The graph represents the average number of IBCs for each UPEC isolate counted in each donor and in each replicate. (B) The bacterial ~~load per well~~concentration was measured by lysing the cells in a second culture that was unfixed to release the intracellular bacteria. ~~Each well was serial diluted and plate counted for colony forming units (CFUs).~~ Data is represented as the average log CFU per well. (C) Representative images of an untreated urothelial cell infection. (D) Representative images of urothelial cell infection treated with 150 µg/mL enrofloxacin (ENRO). (E) Representative images of urothelial cell infection treated with 50 µg/mL cefpodoxime (POD). (F) Representative images of urothelial cell infection treated with 50 µg/mL doxycycline (DOX). Bars are means and standard deviations. Data was analyzed by an ~~and significant differences ($p<0.05$) as determined by~~ ANOVA with Tukey post-hoc. Statistical differences ($p<0.05$) between groups are ~~are~~ indicated by differing letters.

Discussion

~~We established the ability of canine derived uropathogenic *Escherichia coli* (UPEC) to form intracellular bacterial communities (IBCs) in canine urothelial cells. This study also demonstrated the feasibility of the dog as a translational model of human urinary tract infections (UTIs). Using a 5-year retrospective analysis, we found that UTIs in dogs are dominated by similar uropathogens as those found in human UTIs with similar frequencies of antimicrobial resistance,~~

~~multidrug resistance, and recurrent infection as human UTIs[28–30,34–36]. Next, we evaluated and observed IBCs in urine cytologies and biopsies from dogs with positive *E. coli* urine cultures. After validating the clinical appearance of IBCs in dogs, we sought to determine if UPECs of canine origin could establish IBCs in canine urothelial cells *in vitro*. We discovered that canine UPEC isolates formed robust IBCs in canine urothelial cells and that IBC formation was dependent upon *fimH*. Canine UPEC isolates from recurrent cases formed more robust IBCs then those from non-recurrent cases. Lastly, we also found that IBCs were tolerant to treatment with several classes of antimicrobials when used at concentrations achievable in canine urine after standard dosing. Overall, the results of this manuscript show the potential of the dog as a model of human UTIs and demonstrates a similar pathogenesis in canine UPEC UTIs as in human UPEC UTIs. Future experiments using an *in vivo* canine model of IBCs will be required to validate the model for pathogenesis and drug development studies.~~

Urinary tract infections (UTI) are one of the most common bacterial infections in both humans and dogs[1,50]. Uropathogenic *Escherichia coli* (UPEC) account for >75% of human UTIs[28]. We found that UPEC accounted for 40% of canine urinary tract infections in our retrospective analysis. Although UPEC ~~rates~~ prevalence was~~were~~ lower~~ in dogs,~~, UPEC is still the ~~main~~ most frequent uropathogen causing UTIs in dogs. Recurrent infections are a major concern during the treatment and management of UTIs in both dogs and humans[4,51]. ~~In fact, approximately 20–30% of women with UTIs will develop a recurrent infection within 6 months of the initial UTI[52]. In dogs, w~~We found a slightly higher recurrence rate of 42% in dogs when compared to women[52], which may be due to the case selection: all cases were from a referral hospital and not a primary care clinic. UPEC account for most human recurrent infections associated with the same UPEC strain in >65% of human recurrent UTIs[32,52]. Similarly, in

dogs we found that UPEC accounted for the majority (52%) of recurrent cases. In recent studies, multi-drug resistant UPEC strains are identified in >60% of human isolates[53,54]. We found that 45% of canine UPEC isolates were resistance to at least 3 classes of antimicrobials. In addition, extended spectrum beta-lactamase production (ESBL) has been identified in >30% of human UPEC isolates[36,53]. In our canine population, we observed 33% of canine UPEC isolates harboring one or more beta-lactamase or ESBL~~-~~associated genes.

In both human and murine UTI studies, the formation of intracellular bacterial communities (IBCs) within the urothelium is a key attribute of ~~to~~ UPEC pathogenesis[55]. UPEC readily attach, invade, and colonize urinary epithelial cells[11,56] and this ability is often implicated in recurrent UTIs[57,58]. A study in mice showed UPEC persisting within the bladder epithelium and causing recurrence in one third of the mice[59]. A publication from Rosen et al,. 2007 was the first to show IBCs within sloughed urothelial cells from urine of women with UTI symptoms[27]. In that study, ~~they found evidence of IBCs in~~ 1 out of 5 urine samples had evidence of IBCs and filamentous bacteria~~, which are associated with the IBC lifecycle,~~ in 50% of the urine samples. ~~We evaluated urine samples from dogs with UTI symptoms and a positive UPEC culture.~~ We found evidence of IBC formation and filamentous bacteria in 60% and 77% of urine ~~cultures~~cytologies from UPEC-associated UTIs, respectively. We speculate that the higher rates of IBCs and filamentous bacteria observed in our canine study was because we only evaluated dogs with a positive UPEC culture while Rosen et al., 2007 evaluated UTIs caused by a variety of uropathogens. Other uropathogens such as *Klebsiella pneumoniae* and *Proteus mirabilis* can also form IBCs, but no evidence of IBC formation in gram-positive species has been reported[60–62]. ~~We also evaluated canine bladder biopsies for the presence of IBCs to visualize IBCs *in situ* versus sloughed urothelial cells in urine only.~~ We also found evidence of IBCs within the urothelium of the bladder of dogs with a positive

UPEC culture and UTI symptoms. These IBCs were similar in structure and appearance to the IBCs seen within murine urothelium during experimental UPEC infection[11,27].

Other~~s research groups~~ have shown that human primary urothelial cells, human urothelial cells lines and bladder-on-chip models are able to support IBC formation by UPECs[41,63–65]. Therefore, we hypothesized that canine urothelial cells might be able to support IBC formation as well. Indeed, we demonstrated that most UPEC isolates were able to form IBCs in our *in vitro* canine urothelial cell model. These results correlate well with a murine study showing that most human derived UPECs could form IBCs in a murine *in vivo* UTI model[12,59,64].

The ability of UPEC to adhere to and invade into the urothelium to form IBCs is facilitated by the type 1 pilus-associated adhesin FimH, a protein encoded by the *fimH* gene, and which binds to mannosylated uroplakin-proteins that line the apical surface of urothelial cells in the bladder, triggering bacterial internalization about 3-hours post-infection[12,16,37,38]. Other studies in mice have shown that UPEC lacking the *fimH* gene are unable to form IBCs[12,43]. Therefore, we investigated the ability of canine derived UPEC harboring or lacking the *fimH* gene to from IBCs in our canine urothelial cell model. We found that UPEC isolates lacking the *fimH* gene formed fewer IBCs than those isolates with harboring the *fimH* gene. However, in those UPEC isolates harboring the *fimH* gene we observed a range of IBCs from rare to robust.

We also found that UPECs isolated from recurrent UTI cases formed more IBCs then those UPEC isolates from non-recurrent cases. Other studies have shown that certain SNPs within the *fimH* gene can results in a gain or loss of function in binding to urothelial cells[44,45,69]. If UPEC are unable to bind to the urothelial cell, they are subsequently unable to invade into the cell to form IBCs. Therefore, we hypothesize that this variation and association with recurrence could be due

to the FimH type or SNPs within the *fimH* gene resulting from pathoadaptation to the urinary environment which will be a line of inquiry for future work.

UPEC within IBCs are protected from killing by antimicrobials[6,17]. This protection arises from both decreased penetration of the antimicrobial into the bacterial community within the urothelial cell and the reduced metabolic state of bacteria within the IBC[63,70,71]. ~~Therefore, we set out to determine if our canine derived UPECs forming IBCs with canine urothelial cells were protected from antimicrobial effect, including killing.~~ We infected canine urothelial cells with UPEC strains know to form robust IBCs from previous screening for 24 hours prior to treatment with a variety of ~~first-line~~ antimicrobials from different drug classes used to treat canine UTIs[72,73]; specifically, the fluoroquinolone enrofloxacin, the cephalosporin cefpodoxime, and the tetracycline doxycycline. We challenged the UPEC-infected urothelial cells with concentrations achievable in canine urine after standard dosing of each antimicrobial[74–76]. ~~We found that n~~None of the antimicrobials at clinically relevant concentrations were able to ~~substantially~~ reduce the number of IBCs or the bacterial load greater than 2-log; although some variability was observed in response, this is likely due to intracellularly achievable concentration differences between antimicrobial classes. ~~These result~~Our outcomes ~~s~~ are supported by several studies showing that IBCs are tolerant to antimicrobial treatment[6,17,77]. For example, in a murine bladder infection model treatment with trimethoprim-sulfamethoxazole did not eradicate bacteria within the bladder[17]. Another study showed persistence of UPEC within the bladder epithelium using multiple antibiotics with urine concentration well above the therapeutic threshold of planktonic (single cell) UPEC[6]. In a bladder on chip model, treatment with a beta-lactam, ampicillin, did not reduce the IBC size or distribution within the tissue[46]. These findings

correlate with the clinical observations that approximately 14% of women require a second antibiotic prescription within 28 days of diagnosis[77].

In conclusion, we found that the dog is a robust translational model for human UTIs. A recent review article calls for the development of new animal models that better recapitulate human disease[78]. As the dog is a naturally occurring model of UPEC UTIs it holds promise as a more clinically relevant model then the induced murine or porcine models currently in use[79–83]. The results of this study show that the pathogenesis of canine UPEC UTIs, with the formation of antimicrobial tolerant *fimH* dependent IBCs, are similar to human UTIs caused by UPECs. Importantly, the use of the dog would serve as a One Health model as results from these studies could directly apply to both human and veterinary medicine. However, future work is needed that expands upon the presented in this manuscript into an *in vivo* experimental model that can be used to study pathogenesis and develop novel therapeutics. In addition, direct genomic comparisons of canine and human UPECs could be useful in further assessing the canine UTI model.

Methods

Bacterial strains

Clinical UPEC isolates (n = 206) were obtained from routine clinical aerobic cultures of canine urine submitted to the North Carolina State University's College of Veterinary Medicine (NCSU-CVM), Microbiology and Molecular Diagnostics Laboratory (Raleigh, NC). Isolates were included if 1) *E. coli* was the sole etiological agent (monospecies) 2) urine samples were collected via cystocentesis 3) the quantity of *E. coli* recovered was greater than 100,000 CFU/mL as determined by plating of a standard urine volume and 4) the dog was considered to have a UTI based on clinical presentation and diagnosis by the attending veterinarian. All isolates were

confirmed as *E. coli* using matrix assisted laser desorption/ionisation time of flight mass spectrometry (MALDI-TOF)(Vitek MS, Biomerieux; Marcy-l'Étoile, France). Isolates were saved as frozen stocks on glycerol at -80°C. Antimicrobial susceptibility testing was performed on all isolates using the broth microdilution method by a commercial system for the clinical report (Sensititre™; ThermoFisher Scientific, Waltham, MA) using the Companion Animal MIC Plate (Companion Animal MIC Plate). The minimum inhibitory concentrations (MIC) of each antimicrobial reagent were classified as susceptible (including intermediate) or resistant based on the Clinical & Laboratory Standards Institute (CLSI) breakpoints when available[85]. Isolates ~~are~~ were characterized for the following UPEC-associated virulence genes attributed to extraintestinal *E. coli* (ExPEC): *cnf-1*, *hlyA*, *fimH* and *papG* using previously described PCR assays[26].

Recurrent UTI Classification

Medical records of the North Carolina State University (Raleigh, NC) Veterinary Teaching Hospital (VTH) were searched to identify UPEC isolates from dogs that were diagnosed with recurrent UTIs. Isolates were designated as recurrent isolates based on at least ≥2 UTIs confirmed by positive aerobic bacterial cultures of *E. coli* during a 6-month period.

Clinical Specimens

Urine cytologies and bladder biopsies were sourced from ~~North Carolina State University, College of Veterinary Medicine~~NCSU-CVM~~,~~ Clinical Pathology~~,~~ & Immunology ~~and~~ or Histopathology Laboratories (Raleigh, NC). Cytologies and biopsies were chosen for evaluation if the dog had a concurrent positive *Escherichia coli* monoculture by NCSU-CVM’s Clinical Microbiology and Molecular Diagnostics Laboratory, presented with a symptomatic UTI, and had evidence of bacteria by Diff-Quick staining for cultures as assessed by a qualified clinical pathology technician and evidence of inflammation and infection on histology as assessed by a

board-certified clinical pathologist. All cytology and biopsy specimens would have been submitted to the laboratories as part of the clinical and diagnostic work-up at the discretion of the treating veterinarian and were not submitted for inclusion in this study. Owners are notified that remaining sample can be used for research purposes during the patient intake.

Cytologies and biopsies were then assessed for the presence of IBCs by immunofluorescence. For cytologies (n=30), prepared cytospin slides were fixed with 2% paraformaldehyde, washed with sterile phosphate-buffered saline (PBS) and permeabilized with 1% Bovine Serum Albumin (BSA)/0.3% Triton X-100 for 1 hour at room temperature and blocked for 1 hour with 5% BSA. Slides were then incubated overnight with Rabbit Anti-*E. coli* FITC antibody (1:100, ab30522, Abcam, Cambridge, UK) and Mouse Anti-Uroplakin III primary antibody (1:300, ab78196, Abcam, Cambridge, UK). Slides were washed three times in sterile PBS and incubated with the secondary antibody Goat Anti-Mouse IgG H&L Alexa Fluor® 647 (1:1000, ab150115, Abcam, Cambridge, UK) for 1 hour. Slides were then washed again with sterile PBS and mounted with DAPI Fluoromount-G (Southern Biotech, Birmingham, AL). For biopsies (n=10), paraffin-embedded tissue was deparaffinized with Histo-Clear II® (Electron Microscopy Sciences, VWR International, Radnor, PA) followed by ethanol dehydration and heat induced antigen retrieval. Slides were then permeabilized in 1% BSA/0.3% Triton X-100 for 10 minutes and blocked for 1 hour with 5% BSA. Slides were then incubated overnight with Rabbit Anti-*E. coli* FITC antibody (1:100, ab30522, Abcam, Cambridge, UK) and Rabbit Anti-E Cadherin (1:250, ab15148, Abcam, Cambridge, UK). Slides were washed three times in sterile PBS and the secondary antibody Goat Anti-Rabbit IgG H&L Alexa Fluor® 555 (ab150078) (1:1000, ab150115, Abcam, Cambridge, UK) for 1 hour. Slides were then washed again with sterile PBS and mounted with DAPI Fluoromount-G (Southern Biotech, Birmingham, AL).

Stained cytologies and biopsies were imaged on an Olympus IX73 inverted scope with DP80 camera using appropriate fluorescent channels (Olympus Corporation, Shinjuku, Tokyo, Japan).

Canine Urothelial Cell Isolation

Canine bladders (n=10) were aseptically collected postmortem from apparently healthy dogs after planned euthanasia by a North Carolina ~~n~~ animal control facility. The dogs were euthanized by the local animal control facility based on unfit temperament for adoption at the discretion of the facility; the authors had no role in the decision to euthanize and were notified of the planned euthanasia prior to attending. ~~.~~ No dogs were euthanized for the purpose of this study. Dogs were apparently healthy, in good body condition and did not show any signs of urinary or renal disease. ~~The dogs were euthanized by the local animal control facility based on unfit temperament for adoption. No dogs were euthanized for the purpose of this study.~~ At the time of collection, the distal abdomen was aseptically prepped, and a surgical incision was made to reach the urinary bladder. A~~sterile~~ urine sample was collected for culture after surgical incision ~~into the distal abdomen~~ by cystocentesis prior to removing the bladder. The bladder was removed by severing of the ureters and ligaments and excising the bladder at the neck before placing the bladder in PBS containing antibiotic-antimycotic (Gibco® Antibiotic-Antimycotic ThermoFisher Scientific, Waltham, MA). Bladders were transported to the laboratory on ice and all subsequent work was completed in a biosafety cabinet. The bladder was turned inside out to expose the mucosa. Mucosa was evaluated for any noticeable gross pathology. If a bladder had a positive urine culture or the mucosa appeared grossly abnormal, the bladder or any isolated cells were excluded from further use. The mucosa neck of the bladder was sutured closed to ensure only mucosa was exposed. The tissue was then suspended in an Erlenmeyer flask in HBSS containing

antibiotic-antimycotic and 2.4U/mL Gibco® Dispase II (ThermoFisher Scientific, Waltham, MA) and incubated for 2 hours at 5% CO2, 90% humidity, and 37°C under constant rotation (Fig S1A). After 2 hours, any remaining tissue on the mucosal surface was gently scraped off and added to the dispersed cells, passed through a 100µm filter, and centrifuged at 300g for 10 min. The cell pellet was washed and resuspended in fresh HBSS and a live cell count was determined using a Cellometer® Auto 2000 and ViaStainTM AOPI Staining Solution (Nexcelom Bioscience LLC, Lawrence, MA). Cells were then seeded onto Nunc™ Collagen I Coated EasYFlasks™ (ThermoFisher Scientific, Waltham, MA) at 40,000 cells/cm^2 in Urothelial Cell Proliferation Media (UCPM, Gibco™ Defined Keratinocyte SFM containing Defined Keratinocyte-SFM Growth Supplement and antibiotic-antimycotic) and incubated overnight at 5% CO2, 90% humidity, and 37°C. Dead or non-adherent cells were removed the next day by washing with warm PBS three times and adding fresh UCPM. The culture medium was changed every 48 hours for 5-7 days. UCPM is a serum-free media and was used to proliferate urothelial cells while decreasing fibroblast contamination; by the end of 5-7 days, depending on the donor, a pure urothelial cell culture was appreciated by brightfield microscopy and analysis of cellular morphology as described by others (Fig S1B) [40,66,68]. When cultures reached a confluency of 75-95% pure morphologically epithelial-like cells, cells were detached from the growth surface with the use of Invitrogen™ Accutase (0.12 mL/cm^2) (ThermoFisher Scientific, Waltham, MA). Cells were counted again as above and frozen in aliquots of $1{\times}10^6$ cells/mL in liquid nitrogen until future use.

Canine Urothelial Cell Infection and Antimicrobial Treatment

Canine urothelial cells were thawed and seeded at 40,000 cells/cm^2 onto collagen coated 24-well tissue culture plates (Nunc™ Collagen I Coated Multidishes, ThermoFisher Scientific,

Waltham, MA) in UCPM for 24 hours. Dead or non-adherent cells were removed the next day by washing with warm PBS three times and adding fresh UCPM. Cells were grown for another 48 hours, or until 75-95% confluence was reached. Thereafter, the culture media was changed to promote urothelial cell differentiation for expression of uroplakins required for UPEC pathogenesis as described elsewhere[42,86,87]. Briefly, media was changed to Urothelial Cell Differentiation Media (UCDM, 3 parts Dulbecco's modified Eagle's medium to 1-part F12 containing 5% fetal bovine serum, antibiotic-antimycotic and 2mM calcium). Cells were cultured in UCDM for 48 hours, followed by a media change, and then cultured for an additional 48 hours. We have shown that this time frame allows for optimal uroplakin expression in our model (Fig S1C). Thereafter, urothelial cells (n=3 biological replicates per experiment) were infected with various individual UPEC strains (n=30) described above in "Bacterial strains" at a multiplicity of infection (MOI) of 10:1 for 3 hours at 37°C and 5% CO_2. After 3 hours, cells were washed five times with sterile PBS and incubated with media containing 100 μg/ml gentamicin to kill any extracellular bacteria. Infections were carried out for 21 hours after the gentamicin treatment. For antimicrobial challenge experiments, 24 hours post-infection, the gentamicin containing-media was removed, and the cellular monolayer was washed five times with sterile PBS before media containing the appropriate antimicrobial was added for an additional 24 hours. Enrofloxacin (ENRO), cefpodoxime (POD) or doxycycline (DOX) were used at 150, 50 and 50 µg/mL respectively which reflect achievable canine urine concentrations[47–49].

Intracellular Bacterial Community (IBC) Analysis

IBCs were assessed by two methods. First, the intracellular bacterial load was assessed in each culture by lysing urothelial cells and performing serial dilutions and plate counting to determine intracellular CFU[39]. Second, we visualized and quantified IBCs by immunofluorescence.

Briefly, after infection and/or treatment, cultures were washed five times with PBS and fixed with 2% paraformaldehyde. Cultures were then permeabilized with 0.3% Triton X-100 and blocked with 5% BSA prior to staining with Rabbit Anti-*E. coli* FITC (1:100, ab30522, Abcam, Cambridge, UK) and Alexa Fluor™ 555 Phalloidin according to manufacturer's directions (ThermoFisher Scientific, Waltham, MA). Nuclei were stained with DAPI Fluoromount-G® (SouthernBiotech, Birmingham, AL) prior to imaging on an Olympus IX73 inverted scope with DP80 camera using appropriate fluorescent channels (Olympus Corporation, Shinjuku, Tokyo, Japan). To quantify the number of IBCs by visualization, the average number of IBCs observed per high powered field (HPF) were counted; five HPFs at 10x magnification were counted per well. A secondary quantification method was performed to determine the intracellular bacterial load. Using this method, urothelial cell monolayers were washed 3x with PBS and lysed with 0.1% Triton X for 10 minutes. After lysis, serial dilutions were performed, and spot plated in triplicate at each serial dilution for colony counts.

Statistical Analysis

Data in graphs is presented as bars representing the mean and standard deviations. Data was analyzed for normality by the Shapiro- Wilk test. Based on normality, ~~S~~single data points such as IBCs/HPF and log_{10} CFU/mL ~~such as CFU~~ were analyzed ~~using~~ by either a 1-way non-parametric (Kruskal-Wallis Test) or unpaired t-test. A ~~or 1-way/~~ 2-way ANOVA ~~based on normality~~ with Tukey's post hoc test was used to analyze the antimicrobial effects on IBCs~~s.~~. Frequencies were evaluated by a chi-square test. Sex and other covariates were included as fixed effects in the model if indicated. Analysis was performed using JMP Pro 11.0 software (SAS Institute Inc., Cary, NC). All graphs were generated using GraphPad Prism (GraphPad Software Inc., La Jolla California

USA). For all comparisons, $p<0.05$ was considered statistically significant.~~All of the analyses were performed using SAS 9.4 software.~~

Acknowledgements

The authors would like to thank Ms. Jasmin Huang and Ms. Melissa Byrd from the Clinical Microbiology Laboratory at North Carolina State University's College of Veterinary Medicine (NCSU-CVM), Ms. Laura Miller from the Histology Laboratory at NCSU-CVM, Ms. Lynnette McCall from the Clinical Pathology Laboratory at NCSU-CVM, and Ms. Cathryn Hubbard from the Clinical Skills Laboratory at NCSU-CVM.

References

1. Klein RD, Hultgren SJ. Urinary tract infections: microbial pathogenesis, host–pathogen interactions and new treatment strategies. Nat Rev Microbiol. 2020; 1–16. doi:10.1038/s41579-020-0324-0
2. Seguin MA, Vaden SL, Altier C, Stone E, Levine JF. Persistent Urinary Tract Infections and Reinfections in 100 Dogs (1989-1999). Sep, 2003 pp. 622–631. doi:10.1111/j.1939-1676.2003.tb02492.x
3. Mysorekar IU, Hultgren SJ. Mechanisms of uropathogenic Escherichia coli persistence and eradication from the urinary tract. Proceedings of the National Academy of Sciences. 2006;103: 14170–14175. doi:10.1073/pnas.0602136103
4. Norris C, Williams B, Ling G, Franti C, Johnson, Ruby A. Recurrent and persistent urinary tract infections in dogs: 383 cases (1969-1995). J Am Anim Hosp Assoc. 2000;36: 484–492. doi:10.5326/15473317-36-6-484
5. Robino L, Scavone P, Araujo L, Algorta G, Zunino P, Vignoli R. Detection of intracellular bacterial communities in a child with escherichia coli recurrent urinary tract infections. Pathog Dis. 2013;68: 78–81. doi:10.1111/2049-632X.12047
6. Blango MG, Mulvey MA. Persistence of uropathogenic Escherichia coli in the face of multiple antibiotics. Antimicrob Agents Chemother. 2010;54: 1855–63. doi:10.1128/AAC.00014-10
7. Chen SL, Wu M, Henderson JP, Hooton TM, Hibbing ME, Hultgren SJ, et al. Genomic diversity and fitness of E. coli strains recovered from the intestinal and urinary tracts of women with recurrent urinary tract infection. Sci Transl Med. 2013;5. doi:10.1126/SCITRANSLMED.3005497/SUPPL_FILE/5-184RA60_TABLES_S1_TO_S9.ZIP
8. Scott VCS, Haake DA, Churchill BM, Justice SS, Kim JH. Intracellular Bacterial Communities: A Potential Etiology for Chronic Lower Urinary Tract Symptoms. Urology. 2015;86: 425. doi:10.1016/J.UROLOGY.2015.04.002
9. Kern ZT, Jacob ME, Gilbertie JM, Vaden SL, Lyle SK. Characteristics of Dogs with Biofilm-Forming Escherichia Coli Urinary Tract Infections. J Vet Intern Med. 2018;32. doi:10.1111/jvim.15231
10. Hall-Stoodley L, Stoodley P. Evolving concepts in biofilm infections. Cell Microbiol. 2009;11: 1034–1043. doi:10.1111/j.1462-5822.2009.01323.x

11. Anderson GG, Palermo JJ, Schilling JD, Roth R, Heuser J, Hultgren SJ. Intracellular Bacterial Biofilm-Like Pods in Urinary Tract Infections. Science (1979). 2003;301: 105–107. doi:10.1126/science.1084550
12. Martinez JJ. Type 1 pilus-mediated bacterial invasion of bladder epithelial cells. EMBO J. 2000;19: 2803–2812. doi:10.1093/emboj/19.12.2803
13. Dias RCS, Moreira BM, Riley LW. Use of fimH single-nucleotide polymorphisms for strain typing of clinical isolates of Escherichia coli for epidemiologic investigation. 2010;48: 483–8. doi:10.1128/JCM.01858-09
14. Wang H, Min G, Glockshuber R, Sun T-T, Kong X-P. Uropathogenic E. coli adhesin-induced host cell receptor conformational changes: implications in transmembrane signaling transduction. J Mol Biol. 2009;392: 352–61. doi:10.1016/j.jmb.2009.06.077
15. Mulvey MA, Lopez-Boado YS, Wilson CL, Roth R, Parks WC, Heuser J, et al. Induction and Evasion of Host Defenses by Type 1-Piliated Uropathogenic Escherichia coli. Science (1979). 1998;282: 1494–1497. doi:10.1126/science.282.5393.1494
16. Anderson GG, Dodson KW, Hooton TM, Hultgren SJ. Intracellular bacterial communities of uropathogenic Escherichia coli in urinary tract pathogenesis. Trends Microbiol. 2004;12: 424–430. doi:10.1016/J.TIM.2004.07.005
17. Schilling JD, Lorenz RG, Hultgren SJ. Effect of trimethoprim-sulfamethoxazole on recurrent bacteriuria and bacterial persistence in mice infected with uropathogenic Escherichia coli. Infect Immun. 2002;70: 7042–9. doi:10.1128/iai.70.12.7042-7049.2002
18. Barber AE, Norton JP, Wiles TJ, Mulvey MA. Strengths and Limitations of Model Systems for the Study of Urinary Tract Infections and Related Pathologies. Microbiol Mol Biol Rev. 2016;80: 351–67. doi:10.1128/MMBR.00067-15
19. Leenaars CHC, Kouwenaar C, Stafleu FR, Bleich A, Ritskes-Hoitinga M, de Vries RBM, et al. Animal to human translation: A systematic scoping review of reported concordance rates. J Transl Med. 2019;17: 1–22. doi:10.1186/S12967-019-1976-2/FIGURES/11
20. Schneider B, Balbas-Martinez V, Jergens AE, Troconiz IF, Allenspach K, Mochel JP. Model-Based Reverse Translation Between Veterinary and Human Medicine: The One Health Initiative. CPT Pharmacometrics Syst Pharmacol. 2018;7: 65–68. doi:10.1002/PSP4.12262
21. Chiou WL, Jeong HY, Chung SM, Wu TC. Evaluation of using dog as an animal model to study the fraction of oral dose absorbed of 43 drugs in humans. Pharm Res. 2000;17: 135–140. doi:10.1023/A:1007552927404
22. Ogeer-Gyles J, Mathews K, Weese JS, Prescott JF, Boerlin P. Evaluation of catheter-associated urinary tract infections and multi–drug-resistant *Escherichia coli* isolates from the urine of dogs with indwelling urinary catheters. J Am Vet Med Assoc. 2006;229: 1584–1590. doi:10.2460/javma.229.10.1584
23. Windahl U, Holst BS, Nyman A, Grönlund U, Bengtsson B. Characterisation of bacterial growth and antimicrobial susceptibility patterns in canine urinary tract infections. BMC Vet Res. 2014;10: 217. doi:10.1186/s12917-014-0217-4
24. Wong C, Epstein SE, Westropp JL. Antimicrobial Susceptibility Patterns in Urinary Tract Infections in Dogs (2010-2013). J Vet Intern Med. 2015;29: 1045–1052. doi:10.1111/jvim.13571
25. Johnson JR, O'Bryan TT, Low DA, Ling G, Delavari P, Fasching C, et al. Evidence of Commonality between Canine and Human Extraintestinal Pathogenic Escherichia coli Strains That Express papG Allele III. Infect Immun. 2000;68: 3327. doi:10.1128/IAI.68.6.3327-3336.2000
26. Gilbertie J, Levent G, Norman K, Vinasco J, Scott H, Jacob M. Comprehensive phenotypic and genotypic characterization and comparison of virulence, biofilm, and antimicrobial resistance in urinary Escherichia coli isolated from canines. Vet Microbiol. 2020;249. doi:10.1016/J.VETMIC.2020.108822

27. Rosen DA, Hooton TM, Stamm WE, Humphrey PA, Hultgren SJ. Detection of Intracellular Bacterial Communities in Human Urinary Tract Infection. Opal SM, editor. PLoS Med. 2007;4: e329. doi:10.1371/journal.pmed.0040329
28. Foxman B. The epidemiology of urinary tract infection. Nat Rev Urol. 2010;7: 653–660. doi:10.1038/nrurol.2010.190
29. Flores-Mireles AL, Walker JN, Caparon M, Hultgren SJ. Urinary tract infections: epidemiology, mechanisms of infection and treatment options. 2015;13. Available: http://www.ncbi.nlm.nih.gov/pubmed/25853778
30. Medina M, Castillo-Pino E. An introduction to the epidemiology and burden of urinary tract infections. Therapeutic Advances in Urology. SAGE Publications Inc.; 2019. doi:10.1177/1756287219832172
31. Kline KA, Lewis AL. Gram-Positive Uropathogens, Polymicrobial Urinary Tract Infection, and the Emerging Microbiota of the Urinary Tract. Microbiol Spectr. 2016;4. doi:10.1128/microbiolspec.uti-0012-2012
32. Foxman B. Epidemiology of urinary tract infections: incidence, morbidity, and economic costs. Am J Med. 2002;113 Suppl 1A: 5S-13S. doi:10.1016/s0002-9343(02)01054-9
33. Glover M, Moreira CG, Sperandio V, Zimmern P. Recurrent urinary tract infections in healthy and nonpregnant women. Urol Sci. 2014;25: 1–8. doi:10.1016/j.urols.2013.11.007
34. Ramírez-Castillo FY, Moreno-Flores AC, Avelar-González FJ, Márquez-Díaz F, Harel J, Guerrero-Barrera AL. An evaluation of multidrug-resistant Escherichia coli isolates in urinary tract infections from Aguascalientes, Mexico: Cross-sectional study. Ann Clin Microbiol Antimicrob. 2018;17: 34. doi:10.1186/s12941-018-0286-5
35. Ali I, Rafaque Z, Ahmed S, Malik S, Dasti JI. Prevalence of multi-drug resistant uropathogenic Escherichia coli in Potohar region of Pakistan. Asian Pac J Trop Biomed. 2016;6: 60–66. doi:10.1016/j.apjtb.2015.09.022
36. Naziri Z, Derakhshandeh A, Borchaloee AS, Poormaleknia M, Azimzadeh N. Treatment failure in urinary tract infections: A warning witness for virulent multi-drug resistant ESBL-producing Escherichia coli. Infect Drug Resist. 2020;13: 1839–1850. doi:10.2147/IDR.S256131
37. Justice SS, Hung C, Theriot JA, Fletcher DA, Anderson GG, Footer MJ, et al. Differentiation and developmental pathways of uropathogenic Escherichia coli in urinary tract pathogenesis. Proceedings of the National Academy of Sciences. 2004;101: 1333–1338. doi:10.1073/pnas.0308125100
38. Lewis AJ, Richards AC, Mulvey MA. Invasion of Host Cells and Tissues by Uropathogenic Bacteria. Microbiol Spectr. 2016;4. doi:10.1128/microbiolspec.UTI-0026-2016
39. Berry RE, Klumpp DJ, Schaeffer AJ. Urothelial cultures support intracellular bacterial community formation by uropathogenic Escherichia coli. Infect Immun. 2009;77: 2762–72. doi:10.1128/IAI.00323-09
40. Pokrywczynska M, Czapiewska M, Jundzill A, Bodnar M, Balcerczyk D, Kloskowski T, et al. Optimization of porcine urothelial cell cultures: Best practices, recommendations, and threats. Cell Biol Int. 2016;40: 812–820. doi:10.1002/CBIN.10614
41. Berry RE, Klumpp DJ, Schaeffer AJ. Urothelial Cultures Support Intracellular Bacterial Community Formation by Uropathogenic Escherichia coli. Infect Immun. 2009;77: 2762. doi:10.1128/IAI.00323-09
42. Thumbikat P, Berry RE, Schaeffer AJ, Klumpp DJ. Differentiation-induced uroplakin III expression promotes urothelial cell death in response to uropathogenic E. coli. Microbes Infect. 2009;11: 57–65. doi:10.1016/j.micinf.2008.10.008

43. Wright KJ, Seed PC, Hultgren SJ. Development of intracellular bacterial communities of uropathogenic *Escherichia coli* depends on type 1 pili. Cell Microbiol. 2007;9: 2230–2241. doi:10.1111/j.1462-5822.2007.00952.x
44. Schwartz DJ, Kalas V, Pinkner JS, Chen SL, Spaulding CN, Dodson KW, et al. Positively selected FimH residues enhance virulence during urinary tract infection by altering FimH conformation. Proceedings of the National Academy of Sciences. 2013;110: 15530–15537. doi:10.1073/pnas.1315203110
45. Sokurenko E v., Chesnokova V, Dykhuizen DE, Ofek I, Wu X-R, Krogfelt KA, et al. Pathogenic adaptation of Escherichia coli by natural variation of the FimH adhesin. Proceedings of the National Academy of Sciences. 1998;95: 8922–8926. doi:10.1073/pnas.95.15.8922
46. Sharma K, Dhar N, Thacker V v, Simonet TM, Signorino-Gelo F, William Knott G, et al. Dynamic persistence of UPEC intracellular bacterial communities in a human bladder-chip model of urinary tract infection. Elife. 2021;10. doi:10.7554/ELIFE.66481
47. Daniels J, Tracy G, Irom S, Lakritz J. Fluoroquinolone levels in healthy dog urine following a 20-mg/kg oral dose of enrofloxacin exceed mutant prevention concentration targets against Escherichia coli isolated from canine urinary tract infections. J Vet Pharmacol Ther. 2014;37: 201–204. doi:10.1111/JVP.12069
48. Brown S, Arnold T, Hamlow P, Speedy A, Deleeuw N, Hubbard V, et al. Plasma and urine disposition and dose proportionality of ceftiofur and metabolites in dogs after subcutaneous administration of ceftiofur sodium. J Vet Pharmacol Ther. 1995;18: 363–369. doi:10.1111/J.1365-2885.1995.TB00605.X
49. Wilson B, Norris J, Malik R, Martin P, Wigney D, Baral R, et al. Susceptibility of bacteria from feline and canine urinary tract infections to doxycycline and tetracycline concentrations attained in urine four hours after oral dosage. Aust Vet J. 2006;84: 8–11. doi:10.1111/J.1751-0813.2006.TB13115.X
50. Thompson MF, Litster AL, Platell JL, Trott DJ. Canine bacterial urinary tract infections: New developments in old pathogens. The Veterinary Journal. 2011;190: 22–27. doi:10.1016/j.tvjl.2010.11.013
51. Hooton TM. Recurrent urinary tract infection in women. International Journal of Antimicrobial Agents. Elsevier; 2001. pp. 259–268. doi:10.1016/S0924-8579(00)00350-2
52. Glover M, Moreira CG, Sperandio V, Zimmern P. Recurrent urinary tract infections in healthy and nonpregnant women. Urological Science. Elsevier B.V.; 2014. pp. 1–8. doi:10.1016/j.urols.2013.11.007
53. Parajuli NP, Maharjan P, Parajuli H, Joshi G, Paudel D, Sayami S, et al. High rates of multidrug resistance among uropathogenic Escherichia coli in children and analyses of ESBL producers from Nepal. Antimicrob Resist Infect Control. 2017;6: 9. doi:10.1186/s13756-016-0168-6
54. Dehbanipour R, Rastaghi S, Sedighi M, Maleki N, Faghri J. High prevalence of multidrug-resistance uropathogenic Escherichia coli strains, Isfahan, Iran. J Nat Sci Biol Med. 2016;7: 22–6. doi:10.4103/0976-9668.175020
55. Hatt JK, Rather PN. Role of Bacterial Biofilms in Urinary Tract Infections. Springer, Berlin, Heidelberg; 2008. pp. 163–192. doi:10.1007/978-3-540-75418-3_8
56. Delcaru C, Alexandru I, Podgoreanu P, Grosu M, Stavropoulos E, Chifiriuc M, et al. Microbial Biofilms in Urinary Tract Infections and Prostatitis: Etiology, Pathogenicity, and Combating strategies. Pathogens. 2016;5: 65. doi:10.3390/pathogens5040065
57. Soto SM, Smithson A, Horcajada JP, Martinez JA, Mensa JP, Vila J. Implication of biofilm formation in the persistence of urinary tract infection caused by uropathogenic Escherichia coli. Clinical Microbiology and Infection. 2006;12: 1034–1036. doi:10.1111/j.1469-0691.2006.01543.x

58. Ejrnæs K, Stegger M, Reisner A, Ferry S, Monsen T, Holm SE, et al. Characteristics of Escherichia coli causing persistence or relapse of urinary tract infections: Phylogenetic groups, virulence factors and biofilm formation. http://dx.doi.org/104161/viru2618189. 2011;2: 528–537. doi:10.4161/VIRU.2.6.18189
59. Mulvey MA, Schilling JD, Hultgren SJ. Establishment of a persistent Escherichia coli reservoir during the acute phase of a bladder infection. Infect Immun. 2001;69: 4572–9. doi:10.1128/IAI.69.7.4572-4579.2001
60. Rosen DA, Pinkner JS, Jones JM, Walker JN, Clegg S, Hultgren SJ. Utilization of an Intracellular Bacterial Community Pathway in Klebsiella pneumoniae Urinary Tract Infection and the Effects of FimK on Type 1 Pilus Expression. Infect Immun. 2008;76: 3337–3345. doi:10.1128/IAI.00090-08
61. Schaffer JN, Norsworthy AN, Sun T-T, Pearson MM. Proteus mirabilis fimbriae- and urease-dependent clusters assemble in an extracellular niche to initiate bladder stone formation. Proc Natl Acad Sci U S A. 2016;113: 4494–9. doi:10.1073/pnas.1601720113
62. Nielubowicz GR, Mobley HLT. Host–pathogen interactions in urinary tract infection. Nat Rev Urol. 2010;7: 430–441. doi:10.1038/nrurol.2010.101
63. Conover MS, Hadjifrangiskou M, Palermo JJ, Hibbing ME, Dodson KW, Hultgren SJ. Metabolic requirements of Escherichia coli in intracellular bacterial communities during urinary tract infection pathogenesis. mBio. 2016;7: e00104-16. doi:10.1128/mBio.00104-16
64. Garofalo CK, Hooton TM, Martin SM, Stamm WE, Palermo JJ, Gordon JI, et al. Escherichia coli from urine of female patients with urinary tract infections is competent for intracellular bacterial community formation. Infect Immun. 2007;75: 52–60. doi:10.1128/IAI.01123-06
65. Sharma K, Dhar N, Thacker V v., Simonet TM, Signorino-Gelo F, Knott G, et al. Dynamic persistence of intracellular bacterial communities of uropathogenic escherichia coli in a human bladder-chip model of urinary tract infections. Elife. 2021;10. doi:10.7554/ELIFE.66481
66. Kloskowski T, Uzarska M, Gurtowska N, Olkowska J, Joachimiak R, Bajek A, et al. How to isolate urothelial cells? Comparison of four different methods and literature review. Hum Cell. 2014;27: 85–93. doi:10.1007/s13577-013-0070-y
67. Zhang Y, Atala A. Urothelial Cell Culture: Stratified Urothelial Sheet and Three-Dimensional Growth of Urothelial Structure. Humana Press, Totowa, NJ; 2012. pp. 383–399. doi:10.1007/978-1-62703-125-7_23
68. Sangha N. Isolation of Urothelial Cells from Bladder Tissue. Methods in molecular biology (Clifton, NJ). 2013. pp. 21–33. doi:10.1007/978-1-62703-363-3_3
69. Weissman SJ, Beskhlebnaya V, Chesnokova V, Chattopadhyay S, Stamm WE, Hooton TM, et al. Differential Stability and Trade-Off Effects of Pathoadaptive Mutations in the Escherichia coli FimH Adhesin. Infect Immun. 2007;75: 3548–3555. doi:10.1128/IAI.01963-06
70. Walters MC, Roe F, Bugnicourt A, Franklin MJ, Stewart PS, Stewart PS. Contributions of antibiotic penetration, oxygen limitation, and low metabolic activity to tolerance of Pseudomonas aeruginosa biofilms to ciprofloxacin and tobramycin. Antimicrob Agents Chemother. 2003;47: 317–23. doi:10.1128/AAC.47.1.317-323.2003
71. Stewart PS. Antimicrobial Tolerance in Biofilms. Microbial Biofilms, 2nd Edition. 2015;3: 269–285. doi:10.1128/microbiolspec.MB-0010-2014
72. Weese JS, Blondeau JM, Boothe D, Breitschwerdt EB, Guardabassi L, Hillier A, et al. Antimicrobial use guidelines for treatment of urinary tract disease in dogs and cats: antimicrobial guidelines working group of the international society for companion animal infectious diseases. Vet Med Int. 2011;2011: 263768. doi:10.4061/2011/263768
73. Weese JS, Blondeau J, Boothe D, Guardabassi LG, Gumley N, Papich M, et al. International Society for Companion Animal Infectious Diseases (ISCAID) guidelines for the diagnosis and management

of bacterial urinary tract infections in dogs and cats. The Veterinary Journal. 2019;247: 8–25. doi:10.1016/J.TVJL.2019.02.008

74. Frazier DL, Thompson L, Trettien A, Evans EI. Comparison of fluoroquinolone pharmacokinetic parameters after treatment with marbofloxacin, enrofloxacin, and difloxacin in dogs. J Vet Pharmacol Ther. 2008;23: 293–302. doi:10.1111/j.1365-2885.2000.00285.x
75. Daniels JB, Tracy G, Irom SJ, Lakritz J. Fluoroquinolone levels in healthy dog urine following a 20-mg/kg oral dose of enrofloxacin exceed mutant prevention concentration targets against Escherichia coli isolated from canine urinary tract infections. J Vet Pharmacol Ther. 2014;37: 201–204. doi:10.1111/JVP.12069
76. Kumar V, Madabushi R, Lucchesi MBB, Derendorf H. Pharmacokinetics of cefpodoxime in plasma and subcutaneous fluid following oral administration of cefpodoxime proxetil in male beagle dogs. J Vet Pharmacol Ther. 2011;34: 130–135. doi:10.1111/J.1365-2885.2010.01198.X
77. Lawrenson RA, Logie JW. Antibiotic failure in the treatment of urinary tract infections in young women. Journal of Antimicrobial Chemotherapy. 2001;48: 895–901. doi:10.1093/jac/48.6.895
78. Murray BO, Flores C, Williams C, Flusberg DA, Marr EE, Kwiatkowska KM, et al. Recurrent Urinary Tract Infection: A Mystery in Search of Better Model Systems. Front Cell Infect Microbiol. 2021;11: 440. doi:10.3389/FCIMB.2021.691210/BIBTEX
79. Nielsen TK, Petersen NA, Stærk K, Grønnemose RB, Palarasah Y, Nielsen LF, et al. A Porcine Model for Urinary Tract Infection. Front Microbiol. 2019;10. doi:10.3389/FMICB.2019.02564
80. Barber AE, Norton JP, Wiles TJ, Mulvey MA. Strengths and Limitations of Model Systems for the Study of Urinary Tract Infections and Related Pathologies. Microbiol Mol Biol Rev. 2016;80: 351–367. doi:10.1128/MMBR.00067-15
81. Chockalingam A, Stewart S, Xu L, Gandhi A, Matta MK, Patel V, et al. Evaluation of Immunocompetent Urinary Tract Infected Balb/C Mouse Model For the Study of Antibiotic Resistance Development Using Escherichia Coli CFT073 Infection. Antibiotics 2019, Vol 8, Page 170. 2019;8: 170. doi:10.3390/ANTIBIOTICS8040170
82. Hannan TJ, Hunstad DA. A Murine Model for Escherichia coli Urinary Tract Infection. Methods Mol Biol. 2016;1333: 159–75. doi:10.1007/978-1-4939-2854-5_14
83. Hung C-S, Dodson KW, Hultgren SJ. A murine model of urinary tract infection. Nat Protoc. 2009;4: 1230–1243. doi:10.1038/nprot.2009.116
84. LeCuyer TE, Byrne BA, Daniels JB, Diaz-Campos D v, Hammac GK, Miller CB, et al. Population Structure and Antimicrobial Resistance of Canine Uropathogenic Escherichia coli. J Clin Microbiol. 2018;56. doi:10.1128/JCM.00788-18
85. CSLI. Performance Standards for Antimicrobial Susceptibility Testing; Twenty-Seventh Informational Supplement. CLSI Document M100-S27, Clinical and Laboratory Standards Institute, Wayne, Pa. 2017.
86. Thumbikat P, Berry RE, Zhou G, Billips BK, Yaggie RE, Zaichuk T, et al. Bacteria-Induced Uroplakin Signaling Mediates Bladder Response to Infection. Finlay BB, editor. PLoS Pathog. 2009;5: e1000415. doi:10.1371/journal.ppat.1000415
87. Cross WR, Eardley I, Leese HJ, Southgate J. A biomimetic tissue from cultured normal human urothelial cells: analysis of physiological function. American Journal of Physiology-Renal Physiology. 2005;289: F459–F468. doi:10.1152/ajprenal.00040.2005

Canine urothelial cell model to study intracellular bacterial community development by uropathogenic *Escherichia coli*

Canine urothelial cells support intracellular *Escherichia coli*

Jessica M. Gilbertie[1,2], Breanna J. Sheahan[2,3], Shelly L. Vaden[3], Megan E. Jacob[1,2 *]

[1] Department of Population Health and Pathobiology, College of Veterinary Medicine, North Carolina State University, Raleigh, North Carolina, USA

[2]Comparative Medicine Institute, North Carolina State University, Raleigh, NC, USA

[3]Department of Clinical Sciences, College of Veterinary Medicine, North Carolina State University, Raleigh, North Carolina, USA

***Corresponding author:**

Email: mejacob@ncsu.edu

Abstract

Urinary tract infections (UTIs) are the most common bacterial infection of both dogs and humans, with most caused by uropathogenic *Escherichia coli* (UPEC). Recurrent UPEC infections are a major concern in the treatment and management of UTIs in both dogs and humans. In humans, the ability of UPECs to form intracellular bacterial communities (IBCs) within urothelial cells has been implicated in recurrent UTIs. However, the role of IBCs has not been explored in the pathogenesis of canine recurrent UTIs. In this study, we identified IBCs in both urine and bladder tissue from dogs with UPEC associated. In addition, we showed that

UPECs derived from canine UTIs form IBCs within primary canine urothelial cells (5.8 IBCs/HPF and 4.3 log_{10}CFU/well). As in human UTIs, formation of IBCs by canine UPECs correlated with the presence of the *fimH* gene as those isolates lacking the *fimH* gene formed fewer IBCs in canine urothelial cells (0.5 IBCs/HPF and 0.8 log_{10}CFU/well) then those harboring the *fimH* gene (7.7 IBCs/HPF and 5.1 log_{10}CFU/well). Additionally, UPEC strains from clinical cases classified as recurrent UTIs had higher rates of IBC formation (12.8 IBCs/HPF and 7.6 log_{10}CFU/well) than UPEC strains from non-recurrent UTIs (5.5 IBCs/HPF and 4.2 log_{10}CFU/well). These IBCs were tolerant to treatment with enrofloxacin, cefpodoxime and doxycycline at 150, 50 and 50 µg/mL respectively, which are representative of the concentrations achieved in canine urine after standard dosing. This is consistent with the clinical perspective that current UTIs are a common condition of dogs and are difficult to manage through antimicrobial treatment. Additionally, the dog could prove to be a powerful model of IBC formation as they are natural models of UPEC-causing UTIs and have similar pathophysiology of IBC formation.

Introduction

Urinary tract infections (UTI) are the most common bacterial infection of dogs accounting for 14% of medical cases. Uropathogenic *Escherichia coli* (UPEC) are the most common etiological agents in both human and canine UTIs[1,2]. Recurrent infections are a major concern in the treatment and management of UTIs[3]. Previous reports show that 20% of dogs with UTIs will develop a recurrent infection despite aggressive antimicrobial therapy[4]. In humans, recurrent UTIs are associated with the ability of UPEC to form intracellular bacterial communities (IBCs)

that are tolerant to antimicrobial therapy[5–8]; however, the role of IBCs in canine UTIs has not been demonstrated.

There has been an increasing interest in the relationship between UPEC biofilm potential and infection persistence in recurrent UTIs; however, a previous study from our group was unable to identify associations between traditional biofilm formation *in vitro* and clinical recurrence of UTI in dogs[9]. The traditional definition of a biofilm is a community of bacteria within a polymeric matrix that is attached to an abiotic or biotic surface[10]. However, recent advancements in biofilm research suggest that, in the urinary tract, IBCs are considered non-surface attached, intracellular biofilms [11].

In human isolates, the ability of UPEC to adhere to and invade the urothelium to form IBCs is facilitated by the type 1 pilus-associated adhesin FimH[12]. The majority of UPECs encode the *fimH* gene, a part of the *fim* operon, which is transcribed to the FimH adhesion protein[13]. FimH binds to mannose-containing glycoprotein receptors on host cell membranes, which in the urinary tract, are the mannosylated residues on uroplakin proteins that line the apical surface of urothelial cells in the bladder[14]. UPEC utilize binding of FimH to the uroplakin receptor complex to enable adhesion to the urothelial surface[12]. Subsequently, the binding of FimH to uroplakin triggers host cell signaling promoting bacterial internalization[15]. Thereafter, UPEC grow and divide intracellularly forming small clusters of bacteria termed IBCs[16]. Once an IBC develops UPEC are protected, able to tolerate the -cidal activity of antimicrobials[6,17].

Dogs are an ideal translational model for urinary tract infection research. Their size allows for repeated access to large volumes of urine using minimally invasive techniques[18]. Additionally, biopsies of bladder tissue can be easily accomplished via uroscopy. Dogs are commonly used as models of human disease and are great pharmacokinetic models for novel

therapeutics[19–21]. In addition, dogs have similar urinary tract infection pathogenesis, are afflicted by the same types of bacteria, and have urinary biology comparable to humans[2,4,22,23]. For UPEC specific UTIs, dogs are infected by similar sequence types (STs), have similar rates of multi-drug resistant strains, and canine *E. coli* isolates have shown similar virulence traits and biofilm phenotypes as human isolates [24–26].

The goal of this study was to determine if dogs demonstrate similar UPEC associated UTI pathogenesis predominated by IBC formation as observed in humans and murine models[27]. Through retrospective data analysis, we demonstrated that dogs are clinically afflicted by similar uropathogens with similar resistance rates and recurrent infection frequencies as human UTIs. In clinical specimens, we demonstrated that UPEC-causing UTIs were associated with IBCs on both urine cytologies and bladder biopsies in symptomatic dogs; additionally, we validated an *in vitro* IBC model using primary canine urothelial cells. Using this *in vitro* IBC model, we validated the role of *fimH* in IBC formation in urothelial cells, assessed IBCs tolerance to treatment with several classes of clinically relevant antimicrobials, and compared IBCs *in vitro* from UPEC isolated from recurrent UTI cases compared to UPEC isolated from nonrecurrent UTI cases.

Results

Canine UTIs exhibit similar uropathogen distribution, antimicrobial resistance and recurrent infection frequencies as human UTIs

To compare the epidemiology of canine and human UTIs we performed a retrospective analysis of canine uropathogens collected at a veterinary teaching hospital over a 5-year period (2015-2019). During that time, 797 canine urine isolates were obtained, of which 87.3% were

monospecies cultures and 12.7% were polymicrobial. The most common UTI associated pathogen was *Escherichia coli* (39.1% of positive cultures), followed by *Enterococcus* spp. (15.7%), *Streptococcus* spp. (10.7%), *Staphylococcus pseudintermedius* (8%), *Klebsiella pneumoniae* (5.6%), and *Proteus mirabilis* (5.6%) (Fig 1A). In humans, *E. coli* is the most common pathogen causing UTIs as well; however, occurrence is higher with reported rates from 65-75%[28,29]. The second most common UTI associated pathogen of humans varies by study and are similar to canine UTI associated pathogens including *Staphylococcus saprophyticus*, *K. pneumoniae*, *P. mirabilis* and *Enterococcus* spp.[30]. Of the polymicrobial infections in our analysis, the most commonly isolated combination was *Escherichia coli* and *Enterococcus* spp. (typically *faecium* and *faecalis*) (24.5%). The *E. coli*-*Enterococcus* spp. combination is also seen in approximately 36% of human polymicrobial UTIs[31]. The sex distribution was 60.9% female (female intact and female spayed) and 39.1% male (male intact and male castrated) and the median age at presentation was 5.6 years (middle aged adult). Similarly, in human medicine a higher rate of UTIs is observed in women and the highest UTIs rates are seen patients from 18-65 years of age[32].

Of the *Escherichia coli* isolates obtained from either mono- or polymicrobial canine UTIs, 45% were defined as multidrug resistant (MDR; resistant to ≥ 3 antimicrobial classes) (Fig 1B) and 33% demonstrated resistance to beta-lactam antimicrobials based on the presence of *blaCMY* and/or *blaTEM* genes (Fig 1C). In the human literature, 49-68% of UPEC are classified as MDR and 24-40% were extended spectrum beta-lactamase (ESBL) producers[34–36].

Canine patients diagnosed with a recurrent UTI, defined as more than 1 positive urine culture within 6 months of the first urine culture and with the same uropathogen despite antimicrobial therapy, occurred in 42% of cases (Fig 1D). Comparable recurrence rates of 27-53% are observed in human UTIs [28,32]. In parallel to human studies, the most common bacterial

pathogens causing recurrent UTIs in our retrospective analysis were *Escherichia coli* (51.6%) and *Enterococcus* spp. (20.8%) (Fig 1E)[33].

Fig 1. Incidence of various uropathogens in canine urinary tract infections. A retrospective analysis of positive urine cultures submitted to the Clinical Microbiology Laboratory at the North Carolina State University College of Veterinary Medicine were reviewed over a 5-year period (2015-2019). (A) Pathogen incidence of all positive urine cultures (n=797). Corresponding medical records were reviewed to determine if the canine patient was experiencing a recurrent UTI defined by more than one positive culture of the same uropathogen within 6 months of one another. (B) Recurrence rates observed over the 5-year period of the retrospective (C) Pathogen incidence or urine cultures from a recurrent infection. Antimicrobial susceptibility profiles and gene expression for several ESBL genes (standard screening with the Clinical Microbiology Laboratory for any *Escherichia coli*) were reviewed. (D) Incidence of multidrug resistance in the *Escherichia coli* population (n=312) only defined by resistance to greater than or equal to three antimicrobial drug classes. (E) Incidence of at least 1 positive ESBL gene in the *Escherichia coli* population only.

IBCs and filamentous bacteria are observed on urine cytologies and bladder biopsies from dogs diagnosed with UPEC associated UTIs

During human and murine-model UPEC pathogenesis, the bacteria bind to and invade into the cells of the urothelium where they replicate and form IBCs[11,16,37]. After IBCs mature, they will disperse out of the cell to infect neighboring facet cells[38]. When the bacteria emerge from the urothelial cell, they adopt a filamentous phenotype. Both IBCs and filamentous bacteria have been identified in the urine of humans with UTIs[27]. To determine if dogs with naturally occurring UTIs exhibit the presence of IBCs and filamentous bacteria, we evaluated urine

cytologies (n=30) and bladder biopsies (n=10) from dogs with UPEC associated UTIs. Immunofluorescence was used to identify IBCs and filamentous bacteria (Fig 2). In our analysis of urine cytologies by immunofluorescence, we found UPEC IBCs in 60% of the cytologies evaluated (Fig 2A; 18 of 30) and observed filamentous bacteria in 77% of the cytologies evaluated (Fig 2B; 23 of 30). Specifically, IBCs (anti-*E. coli*, green) were appreciated within sheets of urothelial cells (anti-uroplakin III, purple) (Fig 2CD) and individual urothelial cells (Fig 2EF). We also identified filamentous bacteria dispersing from the urothelium-associated IBCs (Fig 2GH). Upon evaluation of the bladder biopsies, we observed IBCs (anti-*E. coli*, green) within the urothelium (anti-Ecadherin, red) in 4 out of the 10 dogs evaluated (Fig 3). The morphology of the IBCs observed in these clinical canine samples reflect those seen in humans and mice[21].

Fig 2. Canine urine cytologies show evidence of IBCs and filamentous bacteria. Urine cytologies were evaluated from dogs exhibiting clinical symptoms of a UTI and a positive *Escherichia coli* culture (n=30). The cytologies were made by the Clinical Pathology Laboratory at North Carolina State University's College of Veterinary Medicine according to their standard operating procedures. The cytologies were then fixed, permeabilized, blocked and stained with anti-Escherichia coli FITC (green) antibody, an anti-uroplakin IIIB antibody with an AlexaFluor 647 secondary antibody (purple) (Abcam, Cambridge, UK) and DAPI (blue) to stain nuclei. (A) The number of urine cytologies evaluated that had observable IBCs within shed urothelial cells. (B) The number of urine cytologies evaluated that had observable filamentous bacteria. (C,D) Lower magnification of large urothelial cell sheets containing both IBCs and filamentous bacteria. (E,F) Higher magnification with white arrows pointing to discrete IBCS within single shed urothelial cells. (G,H) Higher magnification with white arrows pointing to filamentous bacteria emerging from the urothelium.

Fig 3. IBCs are observed within the urothelium of dogs with UPEC-causing UTIs. (A-D) Representative images of IBCs noted within the bladder epithelium of four different canine patients. Briefly, bladder biopsies of dogs presenting to the Internal Medicine Service at North Carolina State University's College of Veterinary Medicine (NCSU-CVM) were evaluated. Biopsies were only evaluated from dogs with clinical signs of a UTI and a positive UPEC culture. Paraffin-embedded sections underwent heat induced antigen retrieval followed by staining with the following antibodies: E-cadherin with an AlexaFluor 555 secondary (red), Escherichia coli-FITC (green) and DAPI to stain nuclei (blue).

Canine urothelial cell cultures support the formation of IBCs by clinical UPEC isolates

Previous research has shown that an immortalized human bladder epithelial cell line (PD07i) supports the growth of IBCs by UPEC *in vitro*[39]. Our aim was to develop an *in vitro* canine UTI model using canine primary urothelial cells and clinical canine UPEC strains. Urothelial cells were harvested and cultured from canine bladders collected post-mortem from healthy dogs without signs of urinary or kidney disease as previously described (Fig S1)[40]. Primary canine urothelial cells (n=3) were then infected with UPEC strains from symptomatic canines with confirmed UTIs (n=30). IBCs were observed 24 hours post-infection (anti-*E. coli*, green) within cultured urothelial cells (F-actin, phalloidin, red) (Fig 4). Specifically, we observed several of the UPEC strains forming IBCs within our canine urothelial cell model with an average of 5.8±5.15 IBCs observed per high powered field (HPF) (Fig 4A) and an average of 4.3±2.63 $\log_{10}$CFU per well (Fig 4B). Five of the 30 (16.7%) UPEC isolates evaluated had no observable IBCs, correlating with <1 $\log_{10}$CFU/mL (Fig 4AB). The IBCs of the canine clinical UPEC strains

grown in canine primary urothelial cells exhibited similar morphology to the IBCs of human UPEC strains grown in an immortalized bladder cell line (Fig 4C-H)[41,42].

Fig S1. Canine urothelial cell isolation and differentiation. Canine bladders (n=10) were aseptically collected postmortem from apparently healthy dogs after planned euthanasia by an animal control facility. The bladder was removed by severing of the ureters and ligaments and excising the bladder at the neck. The bladder was turned inside out to expose the mucosa. The neck of the bladder was sutured closed to ensure only mucosa was exposed. (A) The tissue was then suspended in an Erlenmeyer flask in 200mL HBSS containing antibiotic-antimycotic and 2.4U/mL Dispase II and incubated for 2 hours at 5% CO2, 90% humidity, and 37°C under constant rotation. After 2 hours, any remaining tissue on the mucosal surface was gently scraped off. The cell were pelleted by centrifugation, washed and resuspended in fresh HBSS. Cells were then seeded onto collagen coated flasks at 40,000 cells/cm2 in UCPM. The culture medium was changed every 48 hours for 5-7 days. UCPM is a serum-free media and was used to proliferate urothelial cells while decreasing fibroblast contamination. (B) By 7 days a pure urothelial cell culture was appreciated by brightfield microscopy (10x) and analysis of cellular morphology as described by others[37,63,65]. Urothelial cells were frozen in aliquots and stored in liquid nitrogen until future use. (C) Canine urothelial cells were thawed, and seeded collagen coated 24-well tissue culture plates in UCPM. Cells were grown for 72 hours, or until confluency. Thereafter, the culture media was changed to promote urothelial cell differentiation for expression of uroplakins required for UPEC pathogenesis (termed UCDM) as described elsewhere[39,83,84]. Cells were cultured in UCDM for 48 hours, followed by a media change, and then cultured for an additional 48 hours. Expression of various uroplakins was determined by real-time polymerase chain reaction using

canine specific primers and the housekeeping gene GAPDH. Relative expression compared to the day 0 (before changing to UPDM) is presented at $2^{-\Delta\Delta Ct}$.

Fig 4. Canine primary urothelial cells support IBC formation by canine origin UPECs. Urothelial cells isolated from healthy canine bladders (n=3) were infected with clinical UPEC isolates from cases of canine symptomatic UTIs (n=30) in triplicate for each canine donor. Two hours after infection, gentamicin was added to kill any remaining extracellular bacteria. The infected cultures were then incubated for another 22 hours. Twenty-four hours post-infection, the cultures were washed gently with warm PBS and fixed with paraformaldehyde. After fixation, the cells were permeabilized and blocked prior to staining with an anti-Escherichia coli FITC antibody (green). Cells were also stained with phallodin (red) to stain actin and DAPI (blue) to stain nuclei. (A) The number of IBC per high powered field (HPF) were counted for each donor/UPEC combination. The graph represents the average number of IBCs for each UPEC isolate counted in each donor and in each replicate. (B) The bacterial load per well was measured by lysing the cells in a second culture that was unfixed to release the intracellular bacteria. Each well was serial diluted and plate counted for colony forming units (CFUs). Data is represented as a log CFU per well. (C-E) High-powered representative images (20x) of IBCs observed in infected canine urothelial cells with various canine origin UPEC stains. (F-H) Low-powered representative images (10x).

IBC formation in canine urothelial cell culture is dependent upon *fimH*

Previous research in humans has shown that IBC formation is dependent upon the presence and expression of type 1 pilus or *fimH* in UPEC[12,43]. Therefore, we infected canine urothelial cells from three donors (n=3) with clinical canine UPEC strains that were *fimH* positive (n=9) or

negative (n=9) to determine the association with IBC formation. We found that clinical UPEC isolates containing the *fimH* gene formed significantly more IBCs in canine urothelial cells (7.7 IBCs/HPF) as compared to isolates lacking the *fimH* gene (0.5 IBCs/HPF) as compared (Fig 5A, $p<0.0001$). *FimH* status also affected the bacterial load within urothelial infections, with *fimH* positive isolates establishing a higher CFU/well (5.1 $\log_{10}$CFU/well) as compared to *fimH* negative isolates (0.8 $\log_{10}$CFU/well) (Fig 5B, $p<0.0002$). Representative images of urothelial infections from a *fimH*- UPEC infection (Fig 5C) and *fimH*+ UPEC infection (Fig 5D) further demonstrate that canine origin UPEC stains lacking *fimH* are unable to establish robust IBCs in canine urothelial cells which correlates with results seen in the murine model[12,44,45].

Fig 5. Canine UPEC isolates lacking *fimH* do not form robust IBCs in comparison to isolates that harbor an intact *fimH* gene. Urothelial cells from canine donors (n=3) were isolated, cultured, infected and evaluated as in Fig 4. Specifically, canine urothelial cells were infected with canine UPEC isolates lacking the *fimH* gene (n=9) or those that harbored the *fimH* gene (n=9). The presence or absence of the *fimH* gene was confirmed by PCR as previously described [23]. (A) The number of IBCs per high powered field (HPF) were counted for each donor/UPEC combination. The graph represents the average number of IBCs for each UPEC isolate counted in each donor and in each replicate. (B) The bacterial load per well was measured by lysing the cells in a second culture that was unfixed to release the intracellular bacteria. Each well was serial diluted and plate counted for colony forming units (CFUs). Data is represented as a log CFU per well. (C) Representative images of a urothelial cell infection by a *fimH*- UPEC. (D) Representative images of a urothelial cell infection by a *fimH*+ UPEC. Bars are means and standard deviations and significant differences ($p<0.05$) were determined by an unpaired, nonparametric t-test (for IBCs/HPF) and parametric t-test (for logCFU/mL).

Canine urothelial cell IBC formation is more robust in UPEC isolates derived from cases of recurrent UTIs

To date, the genotypic or phenotypic relationship between UPEC strains and recurrent UTIs is unknown. Due to the variability in clinical UPEC isolates ability to form IBCs, we hypothesized that UPEC strains from recurrent UTI cases would form more IBCs *in vitro*. Therefore, we evaluated UPEC strains from recurrent cases (n=12) of symptomatic UTIs treated with appropriate antimicrobials with at least once recurrence within 6 months of the first UTI. We compared these recurrent UPEC strains with matched UPEC strains from non-recurrent cases of symptomatic UTIs. The non-recurrent cases all had well documented follow-up to ensure a second UPEC-associated UTI did not occur within 6 months of the initial UTI. We also tested for the presence of the *fimH* gene within this group. Interestingly, UPEC isolates from recurrent UTIs expressed more *fimH* (97.5%) versus UPEC isolates from non-recurrent cases (84.9%) (Fig S2A). Therefore, we only used UPEC isolates that were *fimH* positive to compare IBC formation between UPEC isolates from recurrent and non-recurrent cases. We observed a significant difference in both number of IBCs and the size of the IBCs from recurrent UPEC strains. UPEC strains from recurrent cases formed more IBCs (12.8 IBCs/HPF) as compared to UPEC from non-recurrent cases (5.5 IBCs/HPF) (Fig 6A, $p<0.001$). UPEC strains from recurrent cases also had a higher intracellular bacterial load per well (7.6 $\log_{10}$CFU/well) then UPEC from non-recurrent cases (4.2 $\log_{10}$CFU/well) (Fig 6B, $p<0.004$). Representative images of urothelial infections show that UPECs from non-recurrent cases (Fig 6C) form less IBCs then UPECs from recurrent cases (Fig 6D). As all the UPEC strains from recurrent and non-recurrent cases were confirmed *fimH*+, further investigation into the mechanisms behind the increased ability of recurrent UPEC strains to form robust IBCs is warranted as *fimH* is not the only gene involved in IBC formation.

Fig S2. Canine UPEC isolates from recurrent UTIs correlate with fimH gene expression but not multidrug resistance (MDR). Canine UPEC isolates (n=-206) were classified as recurrent with well-documented recurrence of the same strain within 6 months of the initial UTI. (A) Each isolate was evaluated for the presence or absence of the *fimH* gene was confirmed by PCR as previously described [23]. (B) Incidence of multidrug resistance was defined by resistance to greater than or equal to three antimicrobial drug classes. Bars are means and standard deviations and significant differences (p<0.05) were determined by an unpaired, nonparametric t-test.

Fig 6. Canine UPEC isolates from patients with recurrent UTIs form more robust IBCs then isolates from patients with non-recurrent UTIs. Urothelial cells from canine donors (n=3) were isolated, cultured, infected, and evaluated as in Fig 4. Specifically, canine urothelial cells were infected with canine UPEC isolates from cases with a well-documented recurrence of the same strain within 6 months of the initial UTI (n=12). We matched these UPEC isolates with those from non-recurrent cases with well document follow-up in their medical records to ensure that a second UPEC-causing UTI did not occur within 6 months of the initial episode (n=12). (A) The number of IBCs per high powered field (HPF) were counted for each donor/UPEC combination. The graph represents the average number of IBCs for each UPEC isolate counted in each donor and in each replicate. (B) The bacterial load per well was measured by lysing the cells in a second culture that was unfixed to release the intracellular bacteria. Each well was serial diluted and plate counted for colony forming units (CFUs). Data is represented as a log CFU per well. (C) Representative images of a urothelial cell infection by a non-recurrent UPEC isolate. (D) Representative images of a urothelial cell infection by recurrent UPEC isolate. Bars are means and

standard deviations and significant differences ($p<0.05$) were determined by an unpaired, nonparametric t-test (for IBCs/HPF) and parametric t-test (for logCFU/mL).

IBCs in canine urothelial cell culture are tolerant to antimicrobial treatment

Both *in vitro* and *in vivo* studies have shown that UPEC IBCs persist intracellularly despite treatment with antimicrobials[6,46]. In the clinical setting, dogs with recurrent UTIs typically receive first-line antimicrobials, which despite demonstrating *in vitro* susceptibility, may result in persistent infections, and the dog will remain culture-positive. Therefore, we wished to investigate the antimicrobial tolerance of IBC-forming UPECs to antimicrobials commonly used to treat canine UTIs. We infected canine primary urothelial cells from three separate donors (n=3) with three UPEC isolates (n=3) known to form robust IBCs and allowed IBCs to develop for 24 hours. Subsequently, we treated with infected urothelial cells cultures with enrofloxacin (ENRO, 150µg/mL), cefpodoxime (POD, 100 µg/mL) and doxycycline (DOX, 50µgmL) at the Cmax observed in urine in canine pharmacokinetic studies[47–49]. All the UPEC clinical isolates used for the urothelial infections were susceptible to these antimicrobials as determined by *in vitro* susceptibility testing. We found that no antimicrobial treatment tested could eradicate IBCs considerably at concentrations at least 10x the minimum inhibitory concentration (MIC), (No Treatment (NT) 9.1 IBCs/HPF versus enrofloxacin (ENRO) 5.2 IBCs/HPF, cefpodoxime (POD) 7.9 IBCs/HPF, and doxycycline (DOX) 6.7 IBCs/HPF) (Fig 7A). Additionally, we did not appreciate a greater than 2 log reduction in bacterial load (NT 5.3 log_{10}CFU/well versus ENRO 3.8 log_{10}CFU/well, POD 4.7 log_{10}CFU/well, and DOX 4.1 log_{10}CFU/well, respectively) (Fig 7B). Representative images showing the presence of IBCs (Fig 7C-F) despite antimicrobial treatment at well above the MIC. These results show that, similar to traditional biofilms, IBCs grown in

canine urothelial cells are tolerant to antimicrobials and therefore are likely to persist intracellularly in the face of antimicrobial treatment.

Fig 7. IBCs formed in canine urothelial cells by canine-origin canine UPECs are tolerant to treatment with several antimicrobials from different classes. Urothelial cells from canine donors (n=3) were isolated, cultured, infected and evaluated as in Fig 4. Specifically, canine urothelial cells were infected with canine UPEC isolates known to form robust IBCs based those in Fig 4 (n=3) and susceptible to the antimicrobials tested herein. (A) The number of IBCs per high powered field (HPF) were counted for each donor/UPEC/treatment combination. The graph represents the average number of IBCs for each UPEC isolate counted in each donor and in each replicate. (B) The bacterial load per well was measured by lysing the cells in a second culture that was unfixed to release the intracellular bacteria. Each well was serial diluted and plate counted for colony forming units (CFUs). Data is represented as the average log CFU per well. (C) Representative images of an untreated urothelial cell infection. (D) Representative images of urothelial cell infection treated with 150 µg/mL enrofloxacin (ENRO). (E) Representative images of urothelial cell infection treated with 50 µg/mL cefpodoxime (POD). (F) Representative images of urothelial cell infection treated with 50 µg/mL doxycycline (DOX). Bars are means and standard deviations and significant differences ($p<0.05$) as determined by ANOVA with Tukey post-hoc are indicated by differing letters

Discussion

We established the ability of canine-derived uropathogenic *Escherichia coli* (UPEC) to form intracellular bacterial communities (IBCs) in canine urothelial cells. This study also demonstrated the feasibility of the dog as a translational model of human urinary tract infections (UTIs). Using a 5-year retrospective analysis, we found that UTIs in dogs are dominated by similar

uropathogens as those found in human UTIs with similar frequencies of antimicrobial resistance, multidrug resistance, and recurrent infection as human UTIs[28–30,34–36]. Next, we evaluated and observed IBCs in urine cytologies and biopsies from dogs with positive *E. coli* urine cultures. After validating the clinical appearance of IBCs in dogs, we sought to determine if UPECs of canine origin could establish IBCs in canine urothelial cells *in vitro*. We discovered that canine UPEC isolates formed robust IBCs in canine urothelial cells and that IBC formation was dependent upon *fimH*. Canine UPEC isolates from recurrent cases formed more robust IBCs then those from non-recurrent cases. Lastly, we also found that IBCs were tolerant to treatment with several classes of antimicrobials when used at concentrations achievable in canine urine after standard dosing. Overall, the results of this manuscript show the potential of the dog as a model of human UTIs and demonstrates a similar pathogenesis in canine UPEC UTIs as in human UPEC UTIs. Future experiments using an *in vivo* canine model of IBCs will be required to validate the model for pathogenesis and drug development studies.

Urinary tract infections (UTI) are one of the most common bacterial infections in both humans and dogs[1,50]. Uropathogenic Escherichia coli (UPEC) account for >75% of human UTIs[28]. We found that UPEC accounted for 40% of canine urinary tract infections. Although UPEC rates were lower in dogs, UPEC is still the main uropathogen causing UTIs in dogs. Recurrent infections are a major concern during the treatment and management of UTIs in both dogs and humans[4,51]. In fact, approximately 20-30% of women with UTIs will develop a recurrent infection within 6 months of the initial UTI[52]. In dogs, we found a slightly higher recurrence rate of 42% which may be due to the case selection: all cases were from a referral hospital and not a primary care clinic. UPEC account for most human recurrent infections associated with the same UPEC strain in >65% of human recurrent UTIs[32,52]. Similarly, in

dogs we found that UPEC accounted for the majority (52%) of recurrent cases. In recent studies, multi-drug resistant UPEC strains are identified in >60% of human isolates[53,54]. We found that 45% of canine UPEC isolates were resistance to at least 3 classes of antimicrobials. In addition, extended spectrum beta-lactamase production (ESBL) has been identified in >30% of human UPEC isolates[36,53]. In our canine population, we observed 33% of canine UPEC isolates harboring one or more beta-lactamase or ESBL associated genes.

In both human and murine UTI studies, the formation of intracellular bacterial communities (IBCs) within the urothelium is key to UPEC pathogenesis[55]. UPEC readily attach, invade, and colonize urinary epithelial cells[11,56] and this ability is often implicated in recurrent UTIs[57,58]. A study in mice showed UPEC persisting within the bladder epithelium and causing recurrence in one third of the mice[59]. A publication from Rosen et al,. 2007 was the first to show IBCs within sloughed urothelial cells from urine of women with UTI symptoms[27]. In that study they found evidence of IBCs in 1 out of 5 urine samples and filamentous bacteria, which are associated with the IBC lifecycle, in 50% of the urine samples. We evaluated urine samples from dogs with UTI symptoms and a positive UPEC culture. We found evidence of IBC formation and filamentous bacteria in 60% and 77% of urine cultures, respectively. We speculate that the higher rates of IBCs and filamentous bacteria observed in our canine study was because we only evaluated dogs with a positive UPEC culture while Rosen et al., 2007 evaluated UTIs caused by a variety of uropathogens. Other uropathogens such as *Klebsiella pneumoniae* and *Proteus mirabilis* can also form IBCs, but no evidence of IBC formation in gram-positive species has been reported[60–62]. We also evaluated canine bladder biopsies for the presence of IBCs to visualize IBCs i*n situ* versus sloughed urothelial cells in urine only. We found evidence of IBCs within the urothelium of the bladder of dogs with a positive UPEC culture and UTI symptoms. These IBCs were similar in

structure and appearance to the IBCs seen within murine urothelium during experimental UPEC infection[11,27].

Other research groups have shown that human primary urothelial cells, human urothelial cells lines and bladder-on-chip models are able to support IBC formation by UPECs[41,63–65]. Therefore, we hypothesized that canine urothelial cells might be able to support IBC formation as well. Indeed, we demonstrated that most UPEC isolates were able to form IBCs in our *in vitro* canine urothelial cell model. These results correlate well with a murine study showing that most human derived UPECs could form IBCs in a murine *in vivo* UTI model[12,59,64].

The ability of UPEC to adhere to and invade into the urothelium to form IBCs is facilitated by the type 1 pilus-associated adhesin FimH, a protein encoded by the *fimH* gene, and which binds to mannosylated uroplakin-proteins that line the apical surface of urothelial cells in the bladder, triggering bacterial internalization about 3-hours post-infection[12,16,37,38]. Other studies in mice have shown that UPEC lacking the *fimH* gene are unable to form IBCs[12,43]. Therefore, we investigated the ability of canine derived UPEC harboring or lacking the fimH gene to from IBCs in our canine urothelial cell model. We found that UPEC isolates lacking the *fimH* gene formed fewer IBCs than those isolates with harboring the *fimH* gene. However, in those UPEC isolates harboring the *fimH* gene we observed a range of IBCs from rare to robust.

We also found that UPECs isolated from recurrent UTI cases formed more IBCs then those UPEC isolates from non-recurrent cases. Other studies have shown that certain SNPs within the *fimH* gene can results in a gain or loss of function in binding to urothelial cells[44,45,69]. If UPEC are unable to bind to the urothelial cell, they are subsequently unable to invade into the cell to form IBCs. Therefore, we hypothesize that this variation and association with recurrence could be due

to the FimH type or SNPs within the *fimH* gene resulting from pathoadaptation to the urinary environment which will be a line of inquiry for future work.

UPEC within IBCs are protected from killing by antimicrobials[6,17]. This protection arises from both decreased penetration of the antimicrobial into the bacterial community within the urothelial cell and the reduced metabolic state of bacteria within the IBC[63,70,71]. Therefore, we set out to determine if our canine derived UPECs forming IBCs with canine urothelial cells were protected from antimicrobial effect, including killing. We infected canine urothelial cells with UPEC strains know to form robust IBCs from previous screening for 24 hours prior to treatment with a variety of first-line antimicrobials from different drug classes used to treat canine UTIs[72,73]; specifically, the fluoroquinolone enrofloxacin, the cephalosporin cefpodoxime, and the tetracycline doxycycline. We challenged the UPEC-infected urothelial cells with concentrations achievable in canine urine after standard dosing of each antimicrobial[74–76]. We found that none of the antimicrobials at clinically relevant concentrations were able to substantially reduce the number of IBCs or the bacterial load greater than 2-log. These results are supported by several studies showing that IBCs are tolerant to antimicrobial treatment[6,17,77]. For example, in a murine bladder infection model treatment with trimethoprim-sulfamethoxazole did not eradicate bacteria within the bladder[17]. Another study showed persistence of UPEC within the bladder epithelium using multiple antibiotics with urine concentration well above the therapeutic threshold of planktonic (single cell) UPEC[6]. In a bladder on chip model, treatment with a beta-lactam, ampicillin, did not reduce the IBC size or distribution within the tissue[46]. These findings correlate with the clinical observations that approximately 14% of women require a second antibiotic prescription within 28 days of diagnosis[77].

In conclusion, we found that the dog is a robust translational model for human UTIs. A recent review article calls for the development of new animal models that better recapitulate human disease[78]. As the dog is a naturally occurring model of UPEC UTIs it holds promise as a more clinically relevant model then the induced murine or porcine models currently in use[79–83]. The results of this study show that the pathogenesis of canine UPEC UTIs, with the formation of antimicrobial tolerant *fimH* dependent IBCs, are similar to human UTIs caused by UPECs. Importantly, the use of the dog would serve as a One Health model as results from these studies could directly apply to both human and veterinary medicine. However, future work is needed that expands upon the presented in this manuscript into an *in vivo* experimental model that can be used to study pathogenesis and develop novel therapeutics. In addition, direct genomic comparisons of canine and human UPECs could be useful in further assessing the canine UTI model.

Methods

Bacterial strains

Clinical UPEC isolates (n = 206) were obtained from routine clinical aerobic cultures of canine urine submitted to the North Carolina State University's College of Veterinary Medicine (NCSU-CVM), Microbiology and Molecular Diagnostics Laboratory (Raleigh, NC) at the discretion of the treating clinician. Isolates were included if 1) *E. coli* was the sole etiological agent (monospecies) 2) urine samples were collected via cystocentesis 3) the quantity of *E. coli* recovered was greater than 100,000 CFU/mL as determined by plating of a standard urine volume and 4) the dog was considered to have a UTI based on clinical presentation and diagnosis by the attending veterinarian. All isolates were confirmed as *E. coli* using matrix assisted laser desorption/ionisation time of flight mass spectrometry (MALDI-TOF)(Vitek MS, Biomerieux;

Marcy-l'Étoile, France). Isolates were saved as frozen stocks on glycerol at -80°C. Antimicrobial susceptibility testing was performed on all isolates using the broth microdilution method by a commercial system for the clinical report (Sensititre™; ThermoFisher Scientific, Waltham, MA) using the Companion Animal MIC Plate (Companion Animal MIC Plate). The minimum inhibitory concentrations (MIC) of each antimicrobial reagent were classified as susceptible (including intermediate) or resistant based on the Clinical & Laboratory Standards Institute (CLSI) breakpoints when available[85]. Isolates are characterized for the following UPEC-associated virulence genes attributed to extraintestinal *E. coli* (ExPEC): *cnf-1, hlyA, fimH* and *papG* using previously described PCR assays[26].

Recurrent UTI Classification

Medical records of the North Carolina State University (Raleigh, NC) Veterinary Teaching Hospital (VTH) were searched to identify UPEC isolates from dogs that were diagnosed with recurrent UTIs. Isolates were designated as recurrent isolates based on at least ≥2 UTIs confirmed by positive aerobic bacterial cultures of *E. coli* during a 6-month period.

Clinical Specimens

Urine cytologies and bladder biopsies were sourced from North Carolina State University, College of Veterinary Medicine, Clinical Pathology, Immunology and Histopathology Laboratories (Raleigh, NC). Cytologies and biopsies were submitted at the discretion of the treating clinician as part of a clinical work-up and were not submitted for inclusion in the study. Retrospectively, cytologies and biopsies were chosen for evaluation if the dog had a concurrent positive *Escherichia coli* monoculture by NCSU-CVM's Clinical Microbiology and Molecular Diagnostics Laboratory, presented with a symptomatic UTI, and had evidence of bacteria by Diff-Quick staining for cultures as assessed by a qualified clinical pathology technician and evidence

of inflammation and infection on histology as assessed by a board-certified clinical pathologist. Cytologies and biopsies were then assessed for the presence of IBCs by immunofluorescence. For cytologies (n=30), prepared cytospin slides were fixed with 2% paraformaldehyde, washed with sterile phosphate-buffered saline (PBS) and permeabilized with 1% Bovine Serum Albumin (BSA)/0.3% Triton X-100 for 1 hour at room temperature and blocked for 1 hour with 5% BSA. Slides were then incubated overnight with Rabbit Anti-*E. coli* FITC antibody (1:100, ab30522, Abcam, Cambridge, UK) and Mouse Anti-Uroplakin III primary antibody (1:300, ab78196, Abcam, Cambridge, UK). Slides were washed three times in sterile PBS and incubated with the secondary antibody Goat Anti-Mouse IgG H&L Alexa Fluor® 647 (1:1000, ab150115, Abcam, Cambridge, UK) for 1 hour. Slides were then washed again with sterile PBS and mounted with DAPI Fluoromount-G (Southern Biotech, Birmingham, AL). For biopsies (n=10), paraffin-embedded tissue was deparaffinized with Histo-Clear II® (Electron Microscopy Sciences, VWR International, Radnor, PA) followed by ethanol dehydration and heat induced antigen retrieval. Slides were then permeabilized in 1% BSA/0.3% Triton X-100 for 10 minutes and blocked for 1 hour with 5% BSA. Slides were then incubated overnight with Rabbit Anti-*E. coli* FITC antibody (1:100, ab30522, Abcam, Cambridge, UK) and Rabbit Anti-E Cadherin (1:250, ab15148, Abcam, Cambridge, UK). Slides were washed three times in sterile PBS and the secondary antibody Goat Anti-Rabbit IgG H&L Alexa Fluor® 555 (ab150078) (1:1000, ab150115, Abcam, Cambridge, UK) for 1 hour. Slides were then washed again with sterile PBS and mounted with DAPI Fluoromount-G (Southern Biotech, Birmingham, AL). Stained cytologies and biopsies were imaged on an Olympus IX73 inverted scope with DP80 camera using appropriate fluorescent channels (Olympus Corporation, Shinjuku, Tokyo, Japan).

Canine Urothelial Cell Isolation

Whole canine bladders (n=10) were aseptically collected postmortem from apparently healthy dogs after planned humane euthanasia unrelated to our study by animal control facility personnel using facility established protocols. All dogs were euthanized by the animal control facility for reasons not reported to the study team, and patients were not recruited by study personnel. No decision, input, or demographics were provided from the study team; IACUC approval was not required as the animals were deceased at the time of sample collection. The use of these deceased patients as the bladder cell source was due to the invasive sample collection process, number of cells needed to establish a cell culture model and the biological importance of a bladder to living patients; we also considered the ability to preserve cell integrity by collecting immediately postmortem. The study team was notified the morning of a planned euthanasia and would respond if we could ensure that a specimen would be made available and our laboratory could immediately utilize the specimen. At the time of collection, sterile urine was collected for culture after surgical incision into the distal abdomen by cystocentesis. The bladder was removed by severing of the ureters and ligaments and excising the bladder at the neck before placing the bladder in PBS containing antibiotic-antimycotic (Gibco® Antibiotic-Antimycotic ThermoFisher Scientific, Waltham, MA). Bladders were transported to the laboratory on ice and all subsequent work was completed in a biosafety cabinet. The bladder was turned inside out to expose the mucosa. Mucosa was evaluated for any noticeable gross pathology. If a bladder had a positive urine culture or the mucosa appeared grossly abnormal, the bladder or any isolated cells were excluded from further use. The mucosa neck of the bladder was sutured closed to ensure only mucosa was exposed. The tissue was then suspended in an Erlenmeyer flask in HBSS containing antibiotic-antimycotic and 2.4U/mL Gibco® Dispase II (ThermoFisher Scientific, Waltham, MA) and incubated for 2 hours at 5% CO2, 90% humidity, and 37°C under constant rotation (Fig S1A).

After 2 hours, any remaining tissue on the mucosal surface was gently scraped off and added to the dispersed cells, passed through a 100µm filter, and centrifuged at 300g for 10 min. The cell pellet was washed and resuspended in fresh HBSS and a live cell count was determined using a Cellometer® Auto 2000 and ViaStainTM AOPI Staining Solution (Nexcelom Bioscience LLC, Lawrence, MA). Cells were then seeded onto Nunc™ Collagen I Coated EasYFlasks™ (ThermoFisher Scientific, Waltham, MA) at 40,000 cells/cm^2 in Urothelial Cell Proliferation Media (UCPM, Gibco™ Defined Keratinocyte SFM containing Defined Keratinocyte-SFM Growth Supplement and antibiotic-antimycotic) and incubated overnight at 5% CO2, 90% humidity, and 37°C. Dead or non-adherent cells were removed the next day by washing with warm PBS three times and adding fresh UCPM. The culture medium was changed every 48 hours for 5-7 days. UCPM is a serum-free media and was used to proliferate urothelial cells while decreasing fibroblast contamination; by the end of 5-7 days, depending on the donor, a pure urothelial cell culture was appreciated by brightfield microscopy and analysis of cellular morphology as described by others (Fig S1B) [40,66,68]. When cultures reached a confluency of 75-95% pure morphologically epithelial-like cells, cells were detached from the growth surface with the use of Invitrogen™ Accutase (0.12 mL/cm^2) (ThermoFisher Scientific, Waltham, MA). Cells were counted again as above and frozen in aliquots of 1×10^6 cells/mL in liquid nitrogen until future use.

Canine Urothelial Cell Infection and Antimicrobial Treatment

Canine urothelial cells were thawed and seeded at 40,000 cells/cm^2 onto collagen coated 24-well tissue culture plates (Nunc™ Collagen I Coated Multidishes, ThermoFisher Scientific, Waltham, MA) in UCPM for 24 hours. Dead or non-adherent cells were removed the next day by washing with warm PBS three times and adding fresh UCPM. Cells were grown for another 48

hours, or until 75-95% confluence was reached. Thereafter, the culture media was changed to promote urothelial cell differentiation for expression of uroplakins required for UPEC pathogenesis as described elsewhere[42,86,87]. Briefly, media was changed to Urothelial Cell Differentiation Media (UCDM, 3 parts Dulbecco's modified Eagle's medium to 1-part F12 containing 5% fetal bovine serum, antibiotic-antimycotic and 2mM calcium). Cells were cultured in UCDM for 48 hours, followed by a media change, and then cultured for an additional 48 hours. We have shown that this time frame allows for optimal uroplakin expression in our model (Fig S1C). Thereafter, urothelial cells (n=3 biological replicates per experiment) were infected with various individual UPEC strains (n=30) described above in "Bacterial strains" at a multiplicity of infection (MOI) of 10:1 for 3 hours at 37°C and 5% CO_2. After 3 hours, cells were washed five times with sterile PBS and incubated with media containing 100 µg/ml gentamicin to kill any extracellular bacteria. Infections were carried out for 21 hours after the gentamicin treatment. For antimicrobial challenge experiments, 24 hours post-infection, the gentamicin containing-media was removed, and the cellular monolayer was washed five times with sterile PBS before media containing the appropriate antimicrobial was added for an additional 24 hours. Enrofloxacin (ENRO), cefpodoxime (POD) or doxycycline (DOX) were used at 150, 50 and 50 µg/mL respectively which reflect achievable canine urine concentrations[47–49].

Intracellular Bacterial Community (IBC) Analysis

IBCs were assessed by two methods. First, the intracellular bacterial load was assessed in each culture by lysing urothelial cells and performing serial dilutions and plate counting to determine intracellular CFU[39]. Second, we visualized and quantified IBCs by immunofluorescence. Briefly, after infection and/or treatment, cultures were washed five times with PBS and fixed with 2% paraformaldehyde. Cultures were then permeabilized with 0.3% Triton X-100 and blocked

with 5% BSA prior to staining with Rabbit Anti-*E. coli* FITC (1:100, ab30522, Abcam, Cambridge, UK) and Alexa Fluor™ 555 Phalloidin according to manufacturer's directions (ThermoFisher Scientific, Waltham, MA). Nuclei were stained with DAPI Fluoromount-G® (SouthernBiotech, Birmingham, AL) prior to imaging on an Olympus IX73 inverted scope with DP80 camera using appropriate fluorescent channels (Olympus Corporation, Shinjuku, Tokyo, Japan). To quantify the number of IBCs by visualization, the average number of IBCs observed per high powered field (HPF) were counted; five HPFs at 10x magnification were counted per well. A secondary quantification method was performed to determine the intracellular bacterial load. Using this method, urothelial cell monolayers were washed 3x with PBS and lysed with 0.1% Triton X for 10 minutes. After lysis, serial dilutions were performed, and spot plated in triplicate at each serial dilution for colony counts.

Statistical Analysis

Data was analyzed for normality by the Shapiro- Wilk test. Single data points such as CFU were analyzed using 1-way non-parametric (Kruskal-Wallis Test) or 1-way/2-way ANOVA based on normality with Tukey's post hoc tests. Sex and other covariates were included as fixed effects in the model if indicated. All of the analyses were performed using SAS 9.4 software.

Acknowledgements

The authors would like to thank Ms. Jasmin Huang and Ms. Melissa Byrd from the Clinical Microbiology Laboratory at North Carolina State University's College of Veterinary Medicine (NCSU-CVM), Ms. Laura Miller from the Histology Laboratory at NCSU-CVM, Ms. Lynnette McCall from the Clinical Pathology Laboratory at NCSU-CVM, and Ms. Cathryn Hubbard from the Clinical Skills Laboratory at NCSU-CVM.

References

1. Klein RD, Hultgren SJ. Urinary tract infections: microbial pathogenesis, host–pathogen interactions and new treatment strategies. Nat Rev Microbiol. 2020; 1–16. doi:10.1038/s41579-020-0324-0
2. Seguin MA, Vaden SL, Altier C, Stone E, Levine JF. Persistent Urinary Tract Infections and Reinfections in 100 Dogs (1989-1999). Sep, 2003 pp. 622–631. doi:10.1111/j.1939-1676.2003.tb02492.x
3. Mysorekar IU, Hultgren SJ. Mechanisms of uropathogenic Escherichia coli persistence and eradication from the urinary tract. Proceedings of the National Academy of Sciences. 2006;103: 14170–14175. doi:10.1073/pnas.0602136103
4. Norris C, Williams B, Ling G, Franti C, Johnson, Ruby A. Recurrent and persistent urinary tract infections in dogs: 383 cases (1969-1995). J Am Anim Hosp Assoc. 2000;36: 484–492. doi:10.5326/15473317-36-6-484
5. Robino L, Scavone P, Araujo L, Algorta G, Zunino P, Vignoli R. Detection of intracellular bacterial communities in a child with escherichia coli recurrent urinary tract infections. Pathog Dis. 2013;68: 78–81. doi:10.1111/2049-632X.12047
6. Blango MG, Mulvey MA. Persistence of uropathogenic Escherichia coli in the face of multiple antibiotics. Antimicrob Agents Chemother. 2010;54: 1855–63. doi:10.1128/AAC.00014-10
7. Chen SL, Wu M, Henderson JP, Hooton TM, Hibbing ME, Hultgren SJ, et al. Genomic diversity and fitness of E. coli strains recovered from the intestinal and urinary tracts of women with recurrent urinary tract infection. Sci Transl Med. 2013;5. doi:10.1126/SCITRANSLMED.3005497/SUPPL_FILE/5-184RA60_TABLES_S1_TO_S9.ZIP
8. Scott VCS, Haake DA, Churchill BM, Justice SS, Kim JH. Intracellular Bacterial Communities: A Potential Etiology for Chronic Lower Urinary Tract Symptoms. Urology. 2015;86: 425. doi:10.1016/J.UROLOGY.2015.04.002
9. Kern ZT, Jacob ME, Gilbertie JM, Vaden SL, Lyle SK. Characteristics of Dogs with Biofilm-Forming Escherichia Coli Urinary Tract Infections. J Vet Intern Med. 2018;32. doi:10.1111/jvim.15231
10. Hall-Stoodley L, Stoodley P. Evolving concepts in biofilm infections. Cell Microbiol. 2009;11: 1034–1043. doi:10.1111/j.1462-5822.2009.01323.x
11. Anderson GG, Palermo JJ, Schilling JD, Roth R, Heuser J, Hultgren SJ. Intracellular Bacterial Biofilm-Like Pods in Urinary Tract Infections. Science (1979). 2003;301: 105–107. doi:10.1126/science.1084550
12. Martinez JJ. Type 1 pilus-mediated bacterial invasion of bladder epithelial cells. EMBO J. 2000;19: 2803–2812. doi:10.1093/emboj/19.12.2803
13. Dias RCS, Moreira BM, Riley LW. Use of fimH single-nucleotide polymorphisms for strain typing of clinical isolates of Escherichia coli for epidemiologic investigation. 2010;48: 483–8. doi:10.1128/JCM.01858-09
14. Wang H, Min G, Glockshuber R, Sun T-T, Kong X-P. Uropathogenic E. coli adhesin-induced host cell receptor conformational changes: implications in transmembrane signaling transduction. J Mol Biol. 2009;392: 352–61. doi:10.1016/j.jmb.2009.06.077
15. Mulvey MA, Lopez-Boado YS, Wilson CL, Roth R, Parks WC, Heuser J, et al. Induction and Evasion of Host Defenses by Type 1-Piliated Uropathogenic Escherichia coli. Science (1979). 1998;282: 1494–1497. doi:10.1126/science.282.5393.1494
16. Anderson GG, Dodson KW, Hooton TM, Hultgren SJ. Intracellular bacterial communities of uropathogenic Escherichia coli in urinary tract pathogenesis. Trends Microbiol. 2004;12: 424–430. doi:10.1016/J.TIM.2004.07.005
17. Schilling JD, Lorenz RG, Hultgren SJ. Effect of trimethoprim-sulfamethoxazole on recurrent bacteriuria and bacterial persistence in mice infected with uropathogenic Escherichia coli. Infect Immun. 2002;70: 7042–9. doi:10.1128/iai.70.12.7042-7049.2002

18. Barber AE, Norton JP, Wiles TJ, Mulvey MA. Strengths and Limitations of Model Systems for the Study of Urinary Tract Infections and Related Pathologies. Microbiol Mol Biol Rev. 2016;80: 351–67. doi:10.1128/MMBR.00067-15
19. Leenaars CHC, Kouwenaar C, Stafleu FR, Bleich A, Ritskes-Hoitinga M, de Vries RBM, et al. Animal to human translation: A systematic scoping review of reported concordance rates. J Transl Med. 2019;17: 1–22. doi:10.1186/S12967-019-1976-2/FIGURES/11
20. Schneider B, Balbas-Martinez V, Jergens AE, Troconiz IF, Allenspach K, Mochel JP. Model-Based Reverse Translation Between Veterinary and Human Medicine: The One Health Initiative. CPT Pharmacometrics Syst Pharmacol. 2018;7: 65–68. doi:10.1002/PSP4.12262
21. Chiou WL, Jeong HY, Chung SM, Wu TC. Evaluation of using dog as an animal model to study the fraction of oral dose absorbed of 43 drugs in humans. Pharm Res. 2000;17: 135–140. doi:10.1023/A:1007552927404
22. Ogeer-Gyles J, Mathews K, Weese JS, Prescott JF, Boerlin P. Evaluation of catheter-associated urinary tract infections and multi–drug-resistant *Escherichia coli* isolates from the urine of dogs with indwelling urinary catheters. J Am Vet Med Assoc. 2006;229: 1584–1590. doi:10.2460/javma.229.10.1584
23. Windahl U, Holst BS, Nyman A, Grönlund U, Bengtsson B. Characterisation of bacterial growth and antimicrobial susceptibility patterns in canine urinary tract infections. BMC Vet Res. 2014;10: 217. doi:10.1186/s12917-014-0217-4
24. Wong C, Epstein SE, Westropp JL. Antimicrobial Susceptibility Patterns in Urinary Tract Infections in Dogs (2010-2013). J Vet Intern Med. 2015;29: 1045–1052. doi:10.1111/jvim.13571
25. Johnson JR, O'Bryan TT, Low DA, Ling G, Delavari P, Fasching C, et al. Evidence of Commonality between Canine and Human Extraintestinal Pathogenic Escherichia coli Strains That Express papG Allele III. Infect Immun. 2000;68: 3327. doi:10.1128/IAI.68.6.3327-3336.2000
26. Gilbertie J, Levent G, Norman K, Vinasco J, Scott H, Jacob M. Comprehensive phenotypic and genotypic characterization and comparison of virulence, biofilm, and antimicrobial resistance in urinary Escherichia coli isolated from canines. Vet Microbiol. 2020;249. doi:10.1016/J.VETMIC.2020.108822
27. Rosen DA, Hooton TM, Stamm WE, Humphrey PA, Hultgren SJ. Detection of Intracellular Bacterial Communities in Human Urinary Tract Infection. Opal SM, editor. PLoS Med. 2007;4: e329. doi:10.1371/journal.pmed.0040329
28. Foxman B. The epidemiology of urinary tract infection. Nat Rev Urol. 2010;7: 653–660. doi:10.1038/nrurol.2010.190
29. Flores-Mireles AL, Walker JN, Caparon M, Hultgren SJ. Urinary tract infections: epidemiology, mechanisms of infection and treatment options. 2015;13. Available: http://www.ncbi.nlm.nih.gov/pubmed/25853778
30. Medina M, Castillo-Pino E. An introduction to the epidemiology and burden of urinary tract infections. Therapeutic Advances in Urology. SAGE Publications Inc.; 2019. doi:10.1177/1756287219832172
31. Kline KA, Lewis AL. Gram-Positive Uropathogens, Polymicrobial Urinary Tract Infection, and the Emerging Microbiota of the Urinary Tract. Microbiol Spectr. 2016;4. doi:10.1128/microbiolspec.uti-0012-2012
32. Foxman B. Epidemiology of urinary tract infections: incidence, morbidity, and economic costs. Am J Med. 2002;113 Suppl 1A: 5S-13S. doi:10.1016/s0002-9343(02)01054-9
33. Glover M, Moreira CG, Sperandio V, Zimmern P. Recurrent urinary tract infections in healthy and nonpregnant women. Urol Sci. 2014;25: 1–8. doi:10.1016/j.urols.2013.11.007
34. Ramírez-Castillo FY, Moreno-Flores AC, Avelar-González FJ, Márquez-Díaz F, Harel J, Guerrero-Barrera AL. An evaluation of multidrug-resistant Escherichia coli isolates in urinary tract

infections from Aguascalientes, Mexico: Cross-sectional study. Ann Clin Microbiol Antimicrob. 2018;17: 34. doi:10.1186/s12941-018-0286-5

35. Ali I, Rafaque Z, Ahmed S, Malik S, Dasti JI. Prevalence of multi-drug resistant uropathogenic Escherichia coli in Potohar region of Pakistan. Asian Pac J Trop Biomed. 2016;6: 60–66. doi:10.1016/j.apjtb.2015.09.022

36. Naziri Z, Derakhshandeh A, Borchaloee AS, Poormaleknia M, Azimzadeh N. Treatment failure in urinary tract infections: A warning witness for virulent multi-drug resistant ESBL-producing Escherichia coli. Infect Drug Resist. 2020;13: 1839–1850. doi:10.2147/IDR.S256131

37. Justice SS, Hung C, Theriot JA, Fletcher DA, Anderson GG, Footer MJ, et al. Differentiation and developmental pathways of uropathogenic Escherichia coli in urinary tract pathogenesis. Proceedings of the National Academy of Sciences. 2004;101: 1333–1338. doi:10.1073/pnas.0308125100

38. Lewis AJ, Richards AC, Mulvey MA. Invasion of Host Cells and Tissues by Uropathogenic Bacteria. Microbiol Spectr. 2016;4. doi:10.1128/microbiolspec.UTI-0026-2016

39. Berry RE, Klumpp DJ, Schaeffer AJ. Urothelial cultures support intracellular bacterial community formation by uropathogenic Escherichia coli. Infect Immun. 2009;77: 2762–72. doi:10.1128/IAI.00323-09

40. Pokrywczynska M, Czapiewska M, Jundzill A, Bodnar M, Balcerczyk D, Kloskowski T, et al. Optimization of porcine urothelial cell cultures: Best practices, recommendations, and threats. Cell Biol Int. 2016;40: 812–820. doi:10.1002/CBIN.10614

41. Berry RE, Klumpp DJ, Schaeffer AJ. Urothelial Cultures Support Intracellular Bacterial Community Formation by Uropathogenic Escherichia coli. Infect Immun. 2009;77: 2762. doi:10.1128/IAI.00323-09

42. Thumbikat P, Berry RE, Schaeffer AJ, Klumpp DJ. Differentiation-induced uroplakin III expression promotes urothelial cell death in response to uropathogenic E. coli. Microbes Infect. 2009;11: 57–65. doi:10.1016/j.micinf.2008.10.008

43. Wright KJ, Seed PC, Hultgren SJ. Development of intracellular bacterial communities of uropathogenic *Escherichia coli* depends on type 1 pili. Cell Microbiol. 2007;9: 2230–2241. doi:10.1111/j.1462-5822.2007.00952.x

44. Schwartz DJ, Kalas V, Pinkner JS, Chen SL, Spaulding CN, Dodson KW, et al. Positively selected FimH residues enhance virulence during urinary tract infection by altering FimH conformation. Proceedings of the National Academy of Sciences. 2013;110: 15530–15537. doi:10.1073/pnas.1315203110

45. Sokurenko E v., Chesnokova V, Dykhuizen DE, Ofek I, Wu X-R, Krogfelt KA, et al. Pathogenic adaptation of Escherichia coli by natural variation of the FimH adhesin. Proceedings of the National Academy of Sciences. 1998;95: 8922–8926. doi:10.1073/pnas.95.15.8922

46. Sharma K, Dhar N, Thacker V v, Simonet TM, Signorino-Gelo F, William Knott G, et al. Dynamic persistence of UPEC intracellular bacterial communities in a human bladder-chip model of urinary tract infection. Elife. 2021;10. doi:10.7554/ELIFE.66481

47. Daniels J, Tracy G, Irom S, Lakritz J. Fluoroquinolone levels in healthy dog urine following a 20-mg/kg oral dose of enrofloxacin exceed mutant prevention concentration targets against Escherichia coli isolated from canine urinary tract infections. J Vet Pharmacol Ther. 2014;37: 201–204. doi:10.1111/JVP.12069

48. Brown S, Arnold T, Hamlow P, Speedy A, Deleeuw N, Hubbard V, et al. Plasma and urine disposition and dose proportionality of ceftiofur and metabolites in dogs after subcutaneous administration of ceftiofur sodium. J Vet Pharmacol Ther. 1995;18: 363–369. doi:10.1111/J.1365-2885.1995.TB00605.X

49. Wilson B, Norris J, Malik R, Martin P, Wigney D, Baral R, et al. Susceptibility of bacteria from feline and canine urinary tract infections to doxycycline and tetracycline concentrations attained in urine four hours after oral dosage. Aust Vet J. 2006;84: 8–11. doi:10.1111/J.1751-0813.2006.TB13115.X
50. Thompson MF, Litster AL, Platell JL, Trott DJ. Canine bacterial urinary tract infections: New developments in old pathogens. The Veterinary Journal. 2011;190: 22–27. doi:10.1016/j.tvjl.2010.11.013
51. Hooton TM. Recurrent urinary tract infection in women. International Journal of Antimicrobial Agents. Elsevier; 2001. pp. 259–268. doi:10.1016/S0924-8579(00)00350-2
52. Glover M, Moreira CG, Sperandio V, Zimmern P. Recurrent urinary tract infections in healthy and nonpregnant women. Urological Science. Elsevier B.V.; 2014. pp. 1–8. doi:10.1016/j.urols.2013.11.007
53. Parajuli NP, Maharjan P, Parajuli H, Joshi G, Paudel D, Sayami S, et al. High rates of multidrug resistance among uropathogenic Escherichia coli in children and analyses of ESBL producers from Nepal. Antimicrob Resist Infect Control. 2017;6: 9. doi:10.1186/s13756-016-0168-6
54. Dehbanipour R, Rastaghi S, Sedighi M, Maleki N, Faghri J. High prevalence of multidrug-resistance uropathogenic Escherichia coli strains, Isfahan, Iran. J Nat Sci Biol Med. 2016;7: 22–6. doi:10.4103/0976-9668.175020
55. Hatt JK, Rather PN. Role of Bacterial Biofilms in Urinary Tract Infections. Springer, Berlin, Heidelberg; 2008. pp. 163–192. doi:10.1007/978-3-540-75418-3_8
56. Delcaru C, Alexandru I, Podgoreanu P, Grosu M, Stavropoulos E, Chifiriuc M, et al. Microbial Biofilms in Urinary Tract Infections and Prostatitis: Etiology, Pathogenicity, and Combating strategies. Pathogens. 2016;5: 65. doi:10.3390/pathogens5040065
57. Soto SM, Smithson A, Horcajada JP, Martinez JA, Mensa JP, Vila J. Implication of biofilm formation in the persistence of urinary tract infection caused by uropathogenic Escherichia coli. Clinical Microbiology and Infection. 2006;12: 1034–1036. doi:10.1111/j.1469-0691.2006.01543.x
58. Ejrnæs K, Stegger M, Reisner A, Ferry S, Monsen T, Holm SE, et al. Characteristics of Escherichia coli causing persistence or relapse of urinary tract infections: Phylogenetic groups, virulence factors and biofilm formation. http://dx.doi.org/104161/viru2618189. 2011;2: 528–537. doi:10.4161/VIRU.2.6.18189
59. Mulvey MA, Schilling JD, Hultgren SJ. Establishment of a persistent Escherichia coli reservoir during the acute phase of a bladder infection. Infect Immun. 2001;69: 4572–9. doi:10.1128/IAI.69.7.4572-4579.2001
60. Rosen DA, Pinkner JS, Jones JM, Walker JN, Clegg S, Hultgren SJ. Utilization of an Intracellular Bacterial Community Pathway in Klebsiella pneumoniae Urinary Tract Infection and the Effects of FimK on Type 1 Pilus Expression. Infect Immun. 2008;76: 3337–3345. doi:10.1128/IAI.00090-08
61. Schaffer JN, Norsworthy AN, Sun T-T, Pearson MM. Proteus mirabilis fimbriae- and urease-dependent clusters assemble in an extracellular niche to initiate bladder stone formation. Proc Natl Acad Sci U S A. 2016;113: 4494–9. doi:10.1073/pnas.1601720113
62. Nielubowicz GR, Mobley HLT. Host–pathogen interactions in urinary tract infection. Nat Rev Urol. 2010;7: 430–441. doi:10.1038/nrurol.2010.101
63. Conover MS, Hadjifrangiskou M, Palermo JJ, Hibbing ME, Dodson KW, Hultgren SJ. Metabolic requirements of Escherichia coli in intracellular bacterial communities during urinary tract infection pathogenesis. mBio. 2016;7: e00104-16. doi:10.1128/mBio.00104-16
64. Garofalo CK, Hooton TM, Martin SM, Stamm WE, Palermo JJ, Gordon JI, et al. Escherichia coli from urine of female patients with urinary tract infections is competent for intracellular bacterial community formation. Infect Immun. 2007;75: 52–60. doi:10.1128/IAI.01123-06

65. Sharma K, Dhar N, Thacker V v., Simonet TM, Signorino-Gelo F, Knott G, et al. Dynamic persistence of intracellular bacterial communities of uropathogenic escherichia coli in a human bladder-chip model of urinary tract infections. Elife. 2021;10. doi:10.7554/ELIFE.66481

66. Kloskowski T, Uzarska M, Gurtowska N, Olkowska J, Joachimiak R, Bajek A, et al. How to isolate urothelial cells? Comparison of four different methods and literature review. Hum Cell. 2014;27: 85–93. doi:10.1007/s13577-013-0070-y

67. Zhang Y, Atala A. Urothelial Cell Culture: Stratified Urothelial Sheet and Three-Dimensional Growth of Urothelial Structure. Humana Press, Totowa, NJ; 2012. pp. 383–399. doi:10.1007/978-1-62703-125-7_23

68. Sangha N. Isolation of Urothelial Cells from Bladder Tissue. Methods in molecular biology (Clifton, NJ). 2013. pp. 21–33. doi:10.1007/978-1-62703-363-3_3

69. Weissman SJ, Beskhlebnaya V, Chesnokova V, Chattopadhyay S, Stamm WE, Hooton TM, et al. Differential Stability and Trade-Off Effects of Pathoadaptive Mutations in the Escherichia coli FimH Adhesin. Infect Immun. 2007;75: 3548–3555. doi:10.1128/IAI.01963-06

70. Walters MC, Roe F, Bugnicourt A, Franklin MJ, Stewart PS, Stewart PS. Contributions of antibiotic penetration, oxygen limitation, and low metabolic activity to tolerance of Pseudomonas aeruginosa biofilms to ciprofloxacin and tobramycin. Antimicrob Agents Chemother. 2003;47: 317–23. doi:10.1128/AAC.47.1.317-323.2003

71. Stewart PS. Antimicrobial Tolerance in Biofilms. Microbial Biofilms, 2nd Edition. 2015;3: 269–285. doi:10.1128/microbiolspec.MB-0010-2014

72. Weese JS, Blondeau JM, Boothe D, Breitschwerdt EB, Guardabassi L, Hillier A, et al. Antimicrobial use guidelines for treatment of urinary tract disease in dogs and cats: antimicrobial guidelines working group of the international society for companion animal infectious diseases. Vet Med Int. 2011;2011: 263768. doi:10.4061/2011/263768

73. Weese JS, Blondeau J, Boothe D, Guardabassi LG, Gumley N, Papich M, et al. International Society for Companion Animal Infectious Diseases (ISCAID) guidelines for the diagnosis and management of bacterial urinary tract infections in dogs and cats. The Veterinary Journal. 2019;247: 8–25. doi:10.1016/J.TVJL.2019.02.008

74. Frazier DL, Thompson L, Trettien A, Evans EI. Comparison of fluoroquinolone pharmacokinetic parameters after treatment with marbofloxacin, enrofloxacin, and difloxacin in dogs. J Vet Pharmacol Ther. 2008;23: 293–302. doi:10.1111/j.1365-2885.2000.00285.x

75. Daniels JB, Tracy G, Irom SJ, Lakritz J. Fluoroquinolone levels in healthy dog urine following a 20-mg/kg oral dose of enrofloxacin exceed mutant prevention concentration targets against Escherichia coli isolated from canine urinary tract infections. J Vet Pharmacol Ther. 2014;37: 201–204. doi:10.1111/JVP.12069

76. Kumar V, Madabushi R, Lucchesi MBB, Derendorf H. Pharmacokinetics of cefpodoxime in plasma and subcutaneous fluid following oral administration of cefpodoxime proxetil in male beagle dogs. J Vet Pharmacol Ther. 2011;34: 130–135. doi:10.1111/J.1365-2885.2010.01198.X

77. Lawrenson RA, Logie JW. Antibiotic failure in the treatment of urinary tract infections in young women. Journal of Antimicrobial Chemotherapy. 2001;48: 895–901. doi:10.1093/jac/48.6.895

78. Murray BO, Flores C, Williams C, Flusberg DA, Marr EE, Kwiatkowska KM, et al. Recurrent Urinary Tract Infection: A Mystery in Search of Better Model Systems. Front Cell Infect Microbiol. 2021;11: 440. doi:10.3389/FCIMB.2021.691210/BIBTEX

79. Nielsen TK, Petersen NA, Stærk K, Grønnemose RB, Palarasah Y, Nielsen LF, et al. A Porcine Model for Urinary Tract Infection. Front Microbiol. 2019;10. doi:10.3389/FMICB.2019.02564

80. Barber AE, Norton JP, Wiles TJ, Mulvey MA. Strengths and Limitations of Model Systems for the Study of Urinary Tract Infections and Related Pathologies. Microbiol Mol Biol Rev. 2016;80: 351–367. doi:10.1128/MMBR.00067-15

81. Chockalingam A, Stewart S, Xu L, Gandhi A, Matta MK, Patel V, et al. Evaluation of Immunocompetent Urinary Tract Infected Balb/C Mouse Model For the Study of Antibiotic Resistance Development Using Escherichia Coli CFT073 Infection. Antibiotics 2019, Vol 8, Page 170. 2019;8: 170. doi:10.3390/ANTIBIOTICS8040170

82. Hannan TJ, Hunstad DA. A Murine Model for Escherichia coli Urinary Tract Infection. Methods Mol Biol. 2016;1333: 159–75. doi:10.1007/978-1-4939-2854-5_14

83. Hung C-S, Dodson KW, Hultgren SJ. A murine model of urinary tract infection. Nat Protoc. 2009;4: 1230–1243. doi:10.1038/nprot.2009.116

84. LeCuyer TE, Byrne BA, Daniels JB, Diaz-Campos D v, Hammac GK, Miller CB, et al. Population Structure and Antimicrobial Resistance of Canine Uropathogenic Escherichia coli. J Clin Microbiol. 2018;56. doi:10.1128/JCM.00788-18

85. CSLI. Performance Standards for Antimicrobial Susceptibility Testing; Twenty-Seventh Informational Supplement. CLSI Document M100-S27, Clinical and Laboratory Standards Institute, Wayne, Pa. 2017.

86. Thumbikat P, Berry RE, Zhou G, Billips BK, Yaggie RE, Zaichuk T, et al. Bacteria-Induced Uroplakin Signaling Mediates Bladder Response to Infection. Finlay BB, editor. PLoS Pathog. 2009;5: e1000415. doi:10.1371/journal.ppat.1000415

87. Cross WR, Eardley I, Leese HJ, Southgate J. A biomimetic tissue from cultured normal human urothelial cells: analysis of physiological function. American Journal of Physiology-Renal Physiology. 2005;289: F459–F468. doi:10.1152/ajprenal.00040.2005

PLOS One
Editor and Reviewer comments:

We thank the reviewers for their time and thoughtful suggestions on improving this manuscript. There are detailed responses to each concern noted.

Reviewer #1: In this paper, Gilbertie and colleagues describe a series of experiments identifying intracellular uropathogenic E. coli (UPEC) in the urothelial tissues of dogs with UTIs, as well as the capability of canine uroepithelial cells to be propagated and to support an intracellular, IBC-like state for UPEC. Several suggestions for improvement are given below.

Major points:

1. The Introduction could do a better job of specifying what is and what is not known about canine UTI prior to the present study. In the passage starting on line 69, the authors state that "dogs have similar UTI pathogenesis" to humans and that dogs have similar rates of drug-resistant uropathogens, giving multiple references for these statements. Then, in the subsequent paragraph, the authors state that the goal of the present study was to "determine if dogs demonstrate similar UTI pathogenesis" to humans, and that the present paper "demonstrated that dogs are clinically affected by similar uropathogens with similar resistance rates…" On a related note, the authors state (line 70) that dogs have "urinary biology comparable to humans" but this statement needs more specifics. What aspects of urinary biology are similar between the two species?

We agree that the introduction was confusing, and we appreciate the reviewers point. We have edited the introduction to state that we know dogs have similar UTI epidemiology in that they are infected with similar etiologic agents, are infected by similar UPEC sequence types, have similar MDR and ESBL carriage within their UPEC population and are afflicted by similar recurrence rates. However, if canine and human UTIs have a similar pathogenesis is unknown. Therefore, the goal of this manuscript was to determine if IBCs form in canine UTIs in a similar manner as observed in humans.

2. The letter-labels for the panels in Figure 1 don't match between the legend (lines 116-128) and the figure itself.

Thank you for noting this mistake. This has now been corrected.

3. In the paragraph starting on line 250, it appears that 12 recurrent UTI strains and 12 non-recurrent strains were evaluated. But then "97.5%" and "84.9%" of the strains carry fimH, which presumably reflects n=206 (Figure S2 legend)? The text in this paragraph should be clarified as to how many strains were evaluated for each analysis.

We edited this experimental description for clarity.

4. In Figure 7, it isn't clear what is meant by "significant differences are indicated by differing letters" – in other words, what the a's and b's mean in panels A and B.

Letter designations are given when using a Tukey post hoc for multiple comparisons. Therefore, a bar with an "a" above it is statistically different from a bar with any other letter designations such as "b" or "c" but it not different from another "a" or a bar with an "a,b" above it. We tried to clarify that in the figure legend more.

Accompanying this, it would be helpful if in the Discussion, the authors commented on these differences and how they might correlate with the likely intracellular penetration of the chosen antibiotics. Specifically, fluoroquinolones (e.g., enrofloxacin) and doxycycline would be expected to penetrate cells better than a beta-lactam (here cefpodoxime), and there appears to be a correlation between this property and the antibacterial effect as measured by CFU and IBC numbers.

Good point! We have added this observation to the discussion section; in the interest of discussion length as previously noted by reviewers, we did not go into detail as it was not the primary outcome of interest; we can certainly develop this further if the reviewer feels like it would be important.

5. In Figure S2, the measurements of both fimH carriage (panel A) and MDR (panel B) are binary in nature. So, what do the bars represent here? There should not be standard deviations in this type of data. Accompanying that, one would expect that a chi-square analysis rather than “an unpaired, nonparametric t-test” (line 279) would be appropriate for comparisons.

We removed the error bars and performed a chi-square test instead of a t-test. The figure has been updated to represent the edits.

6. The vertebrate animals information would benefit from additional clarification. The authors state that all the samples were collected for clinical purposes and prior to the start of any analysis (i.e., they were banked). What was the clinical indication for bladder biopsies in dogs with active UTIs?

Bladder biopsies are taken during uroscopy at the discretion of the treating veterinarian. The biopsies are sent to the pathology service to evaluate the bladder tissue for the presence of inflammation, infection, or neoplasm. These samples are banked and can be used for research purposes. We have tried to clarify this in the manuscript by adding that these are diagnostic specimens submitted for the management of a patient and not because of inclusion in the study. Still, all samples submitted to the NCSU CVM are approved to be used for research purposes.

Minor points:

1. Given that no methodologic details are not fully available in the abstract, one should remove the specific numerical data from the abstract. For example, “4.2 log10 CFU/well” doesn’t have specific meaning as the reader hasn’t yet read the methods.

This numerical data has been removed from the abstract per this reviewer’s request.

2. Line 44: “20% of dogs with UTIs will develop a recurrent infection…” during what period of follow-up (in reference 4)?

This has been updated.

3. Line 182: Beyond “similar morphology,” can the authors be more specific as to the size of intracellular communities or the apparent number of bacteria, in canine uroepithelial cells vs the immortalized human bladder cell line?

Not at this stage but this would be a good idea for future work.

4. Some of the figure legends could be shortened by reducing methodologic detail (these details already appear in the Methods section).

We have followed this suggestion and reduced the figure legends.

5. The Discussion could probably also be shortened by reducing the degree to which the current Results are recapped.

We have followed this advice and removed paragraph 1 that recapped the results.

6. Panels A and B of Figure 2 could probably be eliminated, as they reflect simple percentages that are described in the text.

We prefer to leave panels A and B in Figure 2 in but will leave this to the discretion of the editor.

Reviewer #2: The authors have written an informative manuscript. However, the authors should proofread the manuscript and correct some minor spelling mistakes and strictly follow the journal's author guidelines.

The sentence in line 22 and 23 is incomplete.

Corrected.

The sentence in line 63 and 64 is not well understood.

We have edited this sentence.

Please check the spelling of ' their) in line 66.

Corrected.

Please elaborate more on the statistical analysis. Most of the statement have been made in the 'results' section. For example, non-parametric t-test.

We have added to the statistical analysis section with statements from our figures/results sections.

The authors did not include control strains in the experimental section.

We did not use any control strains only clinical UPEC strains from canine UTIs as appropriate per the study design.

Authors should please take a second look at the clarity of the labels.

We have double checked the labels and corrected where appropriate. Most of this clarification was addressed through other reviewer comments.

Authors should make comments on the ethical approval.

We have added further comments on the ethical approval.
