# Supplementary material for: Canine urothelial cell model to study intracellular bacterial community development by uropathogenic Escherichia coli
Source: PLoS One. 2025 Jan 9;20(1):e0316834. doi: 10.1371/journal.pone.0316834 (PMC11717241; doi:10.1371/journal.pone.0316834)
Supplement: Attachment — Submitted filename: Cover Letter for canine urothelial model.pdf [file pone.0316834.s008.pdf]

NC STATE UNIVERSITY

College of Agriculture and Life Sciences
Office of the Dean

cals.ncsu.edu

Campus Box 7601
112 Patterson Hall
Raleigh, NC 27695-7601
P: 919.515.2668

December 4, 2024

Dear PLOS One editor:

As corresponding author, I am pleased to submit the revision of *Canine urothelial cell model to study intracellular bacterial community development by uropathogenic Escherichia coli*. I have reviewed the checklist and provided a response to the reviewer comments below:

Reviewer #9: The authors addressed some of the point raised at the first round of revision. However, the following point lacks of response.

"Lines 310-317 These results are not very solid. Are the antibiotics tested capable to permeate epithelial cells? If not, no conclusions can be drawn from these experiments. I suggest to use an antibiotic that penetrate the cells to evaluate if bacteria forming IBCs are somehow resistant or not."

Yes, we did use and have added two references on the two antimicrobials with intracellular activity (doxycycline and enrofloxacin; Line 363).

Reviewer #11: All issues raised in the previous round have been satisfactority addressed, except the one about the duplicates in the reference list. In version R3, References 39 and 88 are the same, as are 50 and 69. Please check these to ensure that they are not meant to refer to different papers by people with the same name prior to acceptance.

We appreciate you pointing this out. We have checked the references and removed the those duplicates.

Thank you for your time and consideration, we look forward to a favorable reply.

With sincere thanks and gratitude

Megan E Jacob

Dr. Megan Jacob, MS, PhD
Professor, Veterinary Microbiology
Senior Associate Dean, Administration
College of Agriculture and Life Sciences
NC State University
