# Supplementary material for: Canine urothelial cell model to study intracellular bacterial community development by uropathogenic Escherichia coli
Source: PLoS One. 2025 Jan 9;20(1):e0316834. doi: 10.1371/journal.pone.0316834 (PMC11717241; doi:10.1371/journal.pone.0316834)
Supplement: Attachment — Submitted filename: Response to Reviewers 2.docx [file pone.0316834.s006.docx]

We thank the reviewers for their continued support and investment in this manuscript. With 11 reviewers, it is challenging to address all comments, but we have provided a point by point response below and continue to look for ways to improve our work.

**Reviewer #3**

Thank you for your manuscript submission. I believe that your study has fundamental problem. As you know, E.coli has a powerful arsenal including a wide range of virulence factors. You can not draw a conclusion on the basis of a virulence factor like FimH or other virulence factor in solo regarding a feature like IBC. There are many paradox in your manuscript which each part deny the other. Hence, my decision regarding the present manuscript is Definite Rejection.

We appreciate your opinion; we never state that FimH is the only gene responsible for IBC formation. We evaluated this gene as it has been shown in other publications in other species to play a vital role in the first step of IBC formation. Therefore, we examined if this gene was important in canine IBC, and we found that it was. As this is in accordance with studies in other mammalian species, it serves to support that IBC pathophysiology is similar in dogs which is the basis of this comparative model. In future studies, we plan to examine additional virulence factors. The goal of this paper, however, is to establish the dog as a relevant UTI model, and additional virulence determination is beyond the scope of this paper.

Reviewer #4

The only concern that has no been addressed is related with the microscope technique selected for visualization and quantification of IBC. Epifluorescence do not allow to differentiate if a bacteria is on the surface of the cell or inside because of the type of images it gave. In this cases, confocal microscopy is the best option as it is possible to take optical images in the z-plane. Confocal microscopy will confirm that the bacteria are really inside the cell and not in the surface.

Thank you for your concern. We believe we have addressed this possibility in the methods section where we describe using gentamicin to kill any extracellular bacteria starting 1 hr post infection. The gentamicin remains in the media until the end of the experiment. We also wash extensively before imaging to remove dead organisms.

Reviewer #6

Please ensure the manuscript is submitted as per guidelines of the journal. This is interesting study, and hard work has been done to provide ne information, but there is significantly change needed to write in research manuscript format. Specific comments are provided in the manuscript. The author has made change as requested from previous review. However, the manuscript is not up to the standard of journal. The method section has all previous work description, and some current work description. Result section has method and discussion.

Thank you for your notes on the formatting of the manuscript. We have simplified several of the results section in this version to remove more of the methods. However, we feel that having a brief leading description of the experiment leads to a better understanding of the results we are presenting. We have formatted our sections like this in other manuscripts submitted to PLoS with success. We hope that the edits we have made have addressed this comment appropriately.

The ANOVA analysis performed, and did not mention the result. For what section did you performed ANOVA? Please elaborate. The figure still needs clear caption. Results for each experiment conducted in this study would added value in terms of data sharing as there are only 9 sample size.

In the statistical analysis, we state the ANOVA was used to assess the antimicrobial treatments. In that specific figure legend (Fig 7), we state that an ANOVA was used. The results of the ANOVA are presented in the figure as differing letters indicating p<0.05 based on the Tukey post hoc analysis. However, we have removed the letters and instead our now presenting the p-values of the untreated control (NT) against each individual antimicrobial treatment (ENRO, POD, DOX). We hope that this presentation is what the reviewer was looking for.

Figure with IBCs and bar graph do not provide enough data to researcher who want to replicate the study in future.

We understand that the bar graphs may not give enough detail for replication. Therefore, we changed the bar graphs to a bar graph with an overlaid scatter plot so individuals can see each individual data point. We hope that aids in the replicability of the data.

Reviewer #7

Authors have written in the Ethics statement that IACUC approval was not required. I think this statement should be revisited.

We have addressed the ethics statement and IACUC need in the manuscript with justification. If additional information is needed, please be specific what is missing so we can be more clear.

Reviewer #8

This study is aimed to investigate whether dogs evidence similar UPEC-associated UTI pathogenesis with IBC formation, and to validate an in vitro IBC model using primary canine urothelial cells. The work overall is solid, although I do have some comments (noted below).

Comments
- In all figures, font types are unreadable, please modify. Similar problems arise with the scale bar in most of the micrographs. We have edited the figures to increase legibility.
- Information regarding the retrospective study performed should be added in Materials and Methods Section, including its ethical approval. We have added information regarding the retrospective analysis into the methods
- L334. Data regarding antimicrobial susceptibility should be shown. We have added the MIC breakpoints to line 334 in replace of “data not shown”.
- Authors should proofread the manuscript and correct some minor spelling mistakes. The manuscript has been re-proofread to catch any spelling mistakes.

Review #9

The authors described the capability of uropathogenic Escherichia coli strains, isolated from canine UTIs, to invade bladder cells and to form the intracellular bacterial communities (IBCs). This represents a very interesting topic for microbiologists characterizing UPEC strains and the paper provides a basis to study the genotypic and phenotypic relationship between human and dog associated UPEC. Images of IBCs are solid and results reported in the manuscript are quite in line with the published data. However, authors should address the following points:

Minor points:
Introduction section should be improved. We have updated this section, however, this comment is not particularly specific so if holes are noted please let us know.
Lines 44-47 Is there any genetic/phylogenetic relationship among human- and dog-associated UPEC? Can the author describe this point in the introduction? The only comparisons that have been performed are presented in paragraph 4. This work is currently being performed in the first author’s laboratory as a collaboration between a veterinary school and medical school in the same geographic region. We expect that manuscript to follow this one in the next year.
Lines 49-51 Can the authors explain the mechanism through which the biofilm is associated to recurrent infections? In paragraph 2 we state that had no found a correlation between traditional surface attached biofilms and strains that cause recurrent infections. Therefore, IBCs, which are biofilm-like, may be associated but yet proven.
Lines 52-54 To be precise, IBCs are bacterial communities with biofilm-like properties. Moreover, the capability to form IBCs depends mainly on the ability of bacteria to be internalized within a eukaryotic cell rather than their biofilm forming activity. Please, rephrase the paragraph. This paragraph has been rephrased.
Lines 63-64 What did the authors mean for antimicrobials? Also molecules from innate immunity? This has been edited for clarity to mean prescribed antimicrobial agents not naturally derived from the innate immune response.
Lines 71-73 See comment above about the genetic/phylogenetic relationship among human- and dog-associated UPEC? To date, there is nothing in the literature and this manuscript focuses on IBC pathophysiology. However, this work is currently the focus of a grant in the first authors laboratory.
Lines 76-77 What did the authors mean for “similar uropathogens”? This has been changed to etiologic agents.
Line 79 Please, replace “cytologies” with “cytology” throughout the text. As we are referring to multiple not individual we would argue to keep as cytologies, similar to biopsy vs biopsies.
Line 108 Did the authors confirm the phenotypic resistance to beta-lactams? Yes, this has been rephrased to indicate this.
Line 277 Ref 23 is not correct. I suggest to replace it with the following: 10.3390/molecules25020316; 10.1186/s12879-018-3467-0. This has been corrected.
Line 335 In my opinion letters are confusing. Please, report statistically significant differences by using asterisks. This has been changed to astericks.
Lines 252-253 This sentence is not correct. There are several papers describing the characteristics of E. coli responsible for recurrent UTIs in humans. Probably the authors refer to those associated with dogs. The authors could only find references showing that recurrent strains carry more virulence genes but no specific genes have been associated apart from fimH which we evaluate in this manuscript. In addition, there is controversy over the associated between surface-associated biofilm grown in a microtiter plate correlating with recurrent infections. Which is why the authors propose that IBCs may have a higher association. Nevertheless, we have rephrased this to better explain our literature findings.

Major points:
Lines 123-125 Did the authors perform real time PCR or PCR? Evaluation of gene expression seems in contrast to the results described in lines 107-108. Can the authors clarify this point? What did the author mean for “standard screening with the Clinical Microbiology Laboratory for any Escherichia coli”

The clinical assessment of virulence and antimicrobial susceptibility as was done when collecting these isolates through clinical canine submissions was performed by conventional PCR. We have corrected the description that inferred real time PCR.

Line 145 Fig. 3 shows huge bacterial communities located beneath the superficial layers of the bladder. Is this right? Usually IBCs are observed within cells at the superficial layer of the epithelium. Can the authors describe this point? I suggest also to insert arrows showing IBCs.

The authors note that they did not point out the autofluorescence that hemorrhage within the bladder epithelium causes. Therefore, we have added a note about that in the figure legend and added arrows to denote the IBCs in each image.

Line 180 Bars in figure 4 should show the IBC-CFU/ml counting for each tested strains to point out differences among strains. In this form the bars are not understandable. Moreover, negative and positive controls should be added in these type of analyses. A lab E. coli strain can be used as negative control. Vice versa, an already reported IBC producing strain should be used as positive control.

We have changed the bars to scatter plots with bars to aid here. As UTI89 is not a canine strain nor any other published strains, we do not have a positive and negative controls specific for our species. We have robust IBC formation noted by several of our strains in addition to those that form no IBCs at all. Therefore, our screening has identified strains we can use moving forward as positive and negative canine-specific controls.

Line 229 Did these fimH negative strains belong to the 30 analyzed? In line 180 the authors reported 5 strains with no capability to form IBCs. Can the authors clarify the origin of these strains? How did the authors evaluate the absence of fimH gene?

Only 3 of the fimH negative strains belonged to the 30 analyzed. We used our repository of 206 to find the other 6. We only had 9 total in all 206. We added this to the text to further clarify.

Lines 260-261 Is this analysis statistically valid? Considering the low number of analyzed isolates anything can be concluded.

Yes, this is statistically valid. Now that the scatter plots are shown you can see how distinct the populations are further supporting the statistics.

Lines 277-278 FIG S2B is not mentioned in the text and due to the lack of significant results I would suggest the authors to delete totally this analysis.

We have removed this graph from the supplemental figure.

Lines 310-317 These results are not very solid. Are the antibiotics tested capable to permeate epithelial cells? If not, no conclusions can be drawn from these experiments. I suggest to use an antibiotic that penetrate the cells to evaluate if bacteria forming IBCs are somehow resistant or not.

We have added text to further clarify this. Even though cephalosporins have lower intracellular penetration then fluoroquinolones, we used their known penetration rates to ensure that the MIC was achieved within the cell. However, we understand that this is not well described and has now been updated throughout the text.

Major points:
Discussion section should be shortened We have removed a paragraph.

Lines 338-340 Please, lower your enthusiasms about the implications of your model. This type of conclusion cannot be drawn based on the reported results. We changed the use of robust to potential to dampen the enthusiasm.

Lines 348-349 As stated before, this conclusion cannot be drawn. See above.
Lines 350-351 As stated before, this conclusion cannot be drawn. See above.
Line 442 See comment above. Are these antibiotics able to permeate the bladder cells? See above.

Line 462 With the current knowledge it is very difficult to assign one specific pathotype to an E. coli isolate. So it would be better to increase the number of gene tested to increase the probability of a correct classifications. The list of genes associated with UPEC isolates is reported in [https://doi.org/10.1007/s10123-022-00235-y](https://nam02.safelinks.protection.outlook.com/?url=https%3A%2F%2Fdoi.org%2F10.1007%2Fs10123-022-00235-y&data=05%7C01%7Cjgilbertie%40vt.vcom.edu%7C805122feab494a9ec5ed08dbdaee89a9%7C9b8f1f7bb0134471a8c499a15ecc8b1c%7C0%7C0%7C638344491483842650%7CUnknown%7CTWFpbGZsb3d8eyJWIjoiMC4wLjAwMDAiLCJQIjoiV2luMzIiLCJBTiI6Ik1haWwiLCJXVCI6Mn0%3D%7C3000%7C%7C%7C&sdata=oLh1JagX2bG1fpu4eyO8ic9isw57Nz0BNCorTXi%2BE%2Fs%3D&reserved=0) and references therein.

We appreciate this evolving field and the additional characterization that is available. At this point, we are most interested in replicating intracellular bacterial communities with canine UTI strains; we could continue to further characterize these isolates but for use in this purpose of this study we don’t think it would change or enhance our findings.

Minor points:
Line 335 Please, use only the acronym. This has been corrected
Line 357 Can the authors compare these rates with published data? We have added this.
Lines 408-410 Are there evidences of the presence of other adhesins involved in bacterial internalization? Not in bladder epithelium. In kidney cells, the P fimbriae are important. In the intestine a variety depending on the pathotype and location are important.
Lines 433-434 In my opinion, a model for canine related UTIs is needed to study canine UTIs. Now there are several advanced models based on human cells to evaluate human UTIs. We disagree respectfully. In our opinion as translational scientists, having large animal models of spontaneous disease can fit the NIH’s species definition of “Dual Purpose with Dual Benefit”. In that discoveries made in the canine model can be translated to human medicine. If you think about new drugs that need to be tested in relevant models, the canine model could be the key step between murine experiments and first-in-human trials. In fact, clinical trials performed in dogs for relevant diseases can be used as preclinical data supporting IND submissions to the FDA. Therefore, showing that the pathophysiology for UPEC-associated UTIs is the first step towards this.
Line 437 What is the meaning of one health model? Probably it would be better to highlight the fact that UPEC strains can infect humans and dogs, if this is correct. For example, are there studies describing the relationship among human and canine isolates? As stated in the above response, One Health approaches are powerful tools to advance both veterinary and human medicine. If one can show similarities in disease processes, than findings in one species can be directly relevant to another species. In addition, new therapeutics that are brought to market can aid both populations of affected species. Of course, understanding zoonotic potential is very important as well. If the pathophysiology of UTIs is similar between dogs and humans, then is an assumption that canine-specific UPEC isolates could infect humans and visa vera. This is currently a line of questioning in the first-authors independent laboratory in collaboration with human urologists in her institutional sphere. In addition, a new UTI therapeutic is being developed in the authors laboratory that has efficacy in treating canine recurrent UTIs. If the canine model can be proven, then this therapeutic has a greater potential to make an impact in humans suffering from recurrent UTIs. But this paper is the necessary first step.

Reviewer 11

In this manuscript, Gilbertie et al provide a characterization of the role of intracellular bacterial communities (IBCs) in urinary tract infections in dogs. The authors have addressed several of the concerns of the reviewers from Round 1, but overall, there are still areas where the manuscript can be improved, and the claims clarified. The review was also made very difficult by the fact that there are many versions of the text, including one with comments between the authors still included. It was therefore hard to access which is the final revised text – the comments below are based on the text with the track changes on. Figure 2 is also missing from the revised manuscript, and Figure S2 is repeated as ‘Figure 2 revised’ which is incorrect.

As all the figures have been revised this issue should be corrected in the resubmission.

Major comments:
IBCs vs intracellular bacteria: It is unclear to me how the authors define and identify IBCs. At the first glance, it appears that the manuscript appears to characterize all intracellular growth of UPEC as IBCs, especially in the later figures. IBCs are only a subset of intracellular bacteria and are characterized by a high intracellular load of bacteria in umbrella cells, with many of the bacteria within the IBCs being smaller and of coccoid shape. These sorts of structures are evident in Figure 3, where the authors clearly indicate these structures in Figure 3 so that the reader can appreciate them better. Thereafter, some clarity is needed as to how IBCs were enumerated in the high-power field images, and how the total bacterial load was enumerated. If the authors have treated every instance of intracellular growth as an IBC, then they should use the term intracellular bacteria rather than IBCs, especially for the data in Figures 5 and 6. If not, then pointing out examples of IBCs, especially in the FimH+ samples would help the reader.

We have added an additional supplemental figure showing the size ranges we observed in 6 dogs from Fig. 4. In addition, we added a microscopy image with arrows pointing towards representative cells with and without IBCs. We did not count cells that had intracellular bacteria that were not aggregated into a community or within a community that was very small (only a few cells). This supplemental figure is noted in the results section that describes Fig. 4 (the first presentation of IBCs. We hope this clarifies the reviewer’s concerns.

Antibiotic treatment data: Please show the individual points in the bar plots of Figure 7 (as shown in the other figures) to help the reader appreciate the spread in CFU. What do the letters a, b represent in the statistical comparisons – the Figure legend does not clarify this either. Please provide p values from an ANOVA test corrected for multiple comparisons. There does appear to be quite a significant decrease in the number of IBCs in the treatment with Enro. The authors need to discuss the differences between antibiotics more clearly in the Main Text and Discussion. It is also unclear why an arbitrary 2log threshold is cited in the Main Text as the cutoff for any significant difference in CFU. The duration of antibiotic treatment is also unclear. These methodological points need to be made clear and if a comparison is made to the results in the bladder-on-chip study that the authors reference in the Discussion, then it is important to note that there was some clearance by Ampicillin in that study (but very delayed compared to clearance of lumenal bacteria.

We have corrected the formatting of the graphs to show individual values based upon your, and other reviewers, notes. In addition, the a,b,c has been changed to astericks as yourself and others noted this as well. We have also added data further described the antibiotics results.

Minor points
Recurrent UTIs: the main text should be edited to clarify that these are canine-specific strains.

We have added canine or dog to several instances to help clarify that these are not human results.

Figure 1: Percentage numbers are missing on the figure-1B and 1C. Corrected.

Amalgamation of some figures: The manuscript would be improved if the number of main figures were reduced. For example, the low power field images in Figure 4 could be moved to a supplementary, and Figures 5 and 6 could be merged to help the reader identify the clear trend FimH-< FimH+
Use of colours in the figures in the paper: The authors could use non-primary colours to enable colour-blind readers to appreciate the images better. UPEC are identified as labelled green in Figures 3 and 4 when they are in fact shown in yellow.

Because we have not received this request from any other review, we are choosing to keep the figures as is and leave it to the discretion of the editor.

Role of other tissue-resident populations in persistent infections: Quiescent intracellular bacteria in the bladder PMID: 16968784 are also thought to contribute to recurrence; similar populations of solitary bacteria were also observed in a recent study with mouse bladder organoids PMID: 34289360. Could the authors comment on whether similar mechanisms might operate in the canine bladder and whether some of the bacteria they observe in the canine tissue might belong to these sub-populations?

While we agree with the premise of the reviewer, our comments on this point as related to the work in this manuscript would only be speculative and we are using other comments on the length and clarity of the discussion to refrain from adding this comment at this time.

Additional papers on human IBCs in human epithelial cells: At Line 171 or 181 depending on whether track changes are off or on, the authors could cite these relevant papers with UTI work in human cell lines: PMID: 32540870, 29352171 in addition to the study with the PD07i line.

With respect to this reviewer, we already have over 85 references and we feel like we have already cited this point as indicated; at this point we are electing to not add additional citations but will defer to the editor.

Comparison with murine models for UTIs in the Discussion: At line 389 or 414 depending on whether track changes is off or on, the authors should also include murine bladder organoids PMID: 34289360 in the discussion, where IBC formation was also shown and visualized by confocal and volumetric electron microscopy.

With respect to this reviewer, we already have over 85 references and do not feel like additional citations for this point will significantly improve the manuscript; at this point we are electing to not add additional citations but will defer to the editor.

Comparison with human models in the Discussion: At lines 390 or 415, the authors should also include references to Transwell models that have been used by several groups PMID: 29352171, 37051302

With respect to this reviewer, we already have over 85 references and we feel like we have already cited this point as indicated; at this point we are electing to not add additional citations but will defer to the editor.
