# Supplementary material for: Canine urothelial cell model to study intracellular bacterial community development by uropathogenic Escherichia coli
Source: PLoS One. 2025 Jan 9;20(1):e0316834. doi: 10.1371/journal.pone.0316834 (PMC11717241; doi:10.1371/journal.pone.0316834)
Supplement: Attachment — Submitted filename: Response to Reviewers 3.docx [file pone.0316834.s007.docx]

Response to Reviewers

Review #9

Lines 56-57 of the revised manuscript track change on, Several papers described the association between IBCs and rUTIs in humans. The authors should underline that this association is still unknown for canine rUTIs. **Corrected.**

Please, use E. coli instead of Escherichia coli. Correct it throughout the text. **Corrected.**
Bars in Fig 4 are still not understandable and informative. The authors should underline that the tested isolates showed high variability in IBCs formation capacity. There are isolates with no capability to be internalized within bladder cells, vice versa there are isolates forming IBCs. Moreover, CFU/ml counting should be expressed for each isolates known to form IBCs. Finally, fig legend has some mistakes, please revise it. **Changed from bars to lines. The black lines denote the mean and standard deviation and the open blue circles represent the individual UPEC strains. The figure legend has also be updated and edited.**
Lines 201-203 of the revised manuscript track change on. The size cannot be expressed in molarity. **Corrected.**
Line 536 of the revised manuscript track change on, the ref 26 does not report the PCR method used to verify the presence of ExPEC associated genes. Which primers did the authors use? Can the authors add the more appropriate reference? **Corrected.**

Reviewer 11

1. The exact duration of antibiotic treatment is still not mentioned. Could the authors include this in the Main Text? **Corrected.**
2. It would be helpful to the reader if the correspondence between the number of stars and the p values are also mentioned in the Figure Legends wherever appropriate. **Corrected.**
3. Line 54: “To date, it is unknown if IBCs play a role in UTI recurrence” some of the evidences regarding IBC playing a role in UTI recurrence can be recognized from movies from mouse models by Justice et. al (2004) and bladder-on-a-chip model by Sharma [et.al](https://nam02.safelinks.protection.outlook.com/?url=http%3A%2F%2Fet.al%2F&data=05%7C02%7Cjgilbertie%40vt.vcom.edu%7C418d4b8cb0b2435dcb0b08dbfb36f73a%7C9b8f1f7bb0134471a8c499a15ecc8b1c%7C0%7C0%7C638379985767398554%7CUnknown%7CTWFpbGZsb3d8eyJWIjoiMC4wLjAwMDAiLCJQIjoiV2luMzIiLCJBTiI6Ik1haWwiLCJXVCI6Mn0%3D%7C3000%7C%7C%7C&sdata=%2FZEirSlYFs66bEX3nMr3GjVMOqQoBWvSMh1UFPXTnbI%3D&reserved=0) (2021). Consider rephrasing the sentence. **Corrected.**
4. Line 176 – 178 and Figure S2a: the unit should be micron and not micromolar. Please use a small ‘m’. **Corrected.**
5. Line 261: Figure S3 not S2. **Corrected.**
6. The figure S3 is not referenced anywhere in the text. Please also mention the p values for this figure. **Corrected.**
7. Line 292: ‘with’ appears twice in the sentence. **Corrected.**
8. Line 293: typo µg/mL **Corrected.**
9. Line 297: what exactly do the authors mean by ‘eradicate considerably’? Do they mean the complete absence of IBCs. Please edit for clarity. **Amended for clarity with numbers in parentheses.**
10. Line 306: could the authors add the appropriate reference for the C/E values for the antibiotics as this is the first time that this parameter is mentioned? **We are unclear what C/E means. We are reporting Cmax or peak concentration in urine based on the referenced canine pharmacokinetics studies. We attempted to reword the sentence to clarify this statement better.**
11. Line 313. Delete one of the extra periods at the end of the sentence. **Corrected.**
12. Figure 7B. The X axis labels are missing, and the description of how the CFU values are obtained is unclear based on the text given in the legend. Are the authors measuring the total bacterial load per well (intracellular and extracellular)? What does ‘unfixed to release the release the intracellular bacteria’ mean? Does it mean that the host cells were lysed? **Corrected for clarity. The gentamicin kills extracellular bacteria, so when we lyse the urothelial cells we are only quantifying the intracellular bacteria.**
13. Reference duplication: Reference 41 is the same as reference 39, and reference 65 is the same as reference 46. **Did the authors mean to refer to other papers written by the same authors in each case? Reference manager issues that have now been corrected.**
