# Supplementary material for: Canine urothelial cell model to study intracellular bacterial community development by uropathogenic Escherichia coli
Source: PLoS One. 2025 Jan 9;20(1):e0316834. doi: 10.1371/journal.pone.0316834 (PMC11717241; doi:10.1371/journal.pone.0316834)
Supplement: Attachment — Submitted filename: Reviewer Comments Canine UPEC.docx [file pone.0316834.s004.docx]

**PLOS One
Editor and Reviewer comments:**

We thank the reviewers for their time and thoughtful suggestions on improving this manuscript. There are detailed responses to each concern noted.

Reviewer #1: In this paper, Gilbertie and colleagues describe a series of experiments identifying intracellular uropathogenic E. coli (UPEC) in the urothelial tissues of dogs with UTIs, as well as the capability of canine uroepithelial cells to be propagated and to support an intracellular, IBC-like state for UPEC. Several suggestions for improvement are given below.

Major points:

1. The Introduction could do a better job of specifying what is and what is not known about canine UTI prior to the present study. In the passage starting on line 69, the authors state that “dogs have similar UTI pathogenesis” to humans and that dogs have similar rates of drug-resistant uropathogens, giving multiple references for these statements. Then, in the subsequent paragraph, the authors state that the goal of the present study was to “determine if dogs demonstrate similar UTI pathogenesis” to humans, and that the present paper “demonstrated that dogs are clinically affected by similar uropathogens with similar resistance rates…” On a related note, the authors state (line 70) that dogs have “urinary biology comparable to humans” but this statement needs more specifics. What aspects of urinary biology are similar between the two species?

We agree that the introduction was confusing, and we appreciate the reviewers point. We have edited the introduction to state that we know dogs have similar UTI epidemiology in that they are infected with similar etiologic agents, are infected by similar UPEC sequence types, have similar MDR and ESBL carriage within their UPEC population and are afflicted by similar recurrence rates. However, if canine and human UTIs have a similar pathogenesis is unknown. Therefore, the goal of this manuscript was to determine if IBCs form in canine UTIs in a similar manner as observed in humans.

2. The letter-labels for the panels in Figure 1 don’t match between the legend (lines 116-128) and the figure itself.

Thank you for noting this mistake. This has now been corrected.

3. In the paragraph starting on line 250, it appears that 12 recurrent UTI strains and 12 non-recurrent strains were evaluated. But then “97.5%” and “84.9%” of the strains carry fimH, which presumably reflects n=206 (Figure S2 legend)? The text in this paragraph should be clarified as to how many strains were evaluated for each analysis.

We edited this experimental description for clarity.

4. In Figure 7, it isn’t clear what is meant by “significant differences are indicated by differing letters” – in other words, what the a’s and b’s mean in panels A and B.

Letter designations are given when using a Tukey post hoc for multiple comparisons. Therefore, a bar with an “a” above it is statistically different from a bar with any other letter designations such as “b” or “c” but it not different from another “a” or a bar with an “a,b” above it. We tried to clarify that in the figure legend more.

Accompanying this, it would be helpful if in the Discussion, the authors commented on these differences and how they might correlate with the likely intracellular penetration of the chosen antibiotics. Specifically, fluoroquinolones (e.g., enrofloxacin) and doxycycline would be expected to penetrate cells better than a beta-lactam (here cefpodoxime), and there appears to be a correlation between this property and the antibacterial effect as measured by CFU and IBC numbers.

Good point! We have added this observation to the discussion section; in the interest of discussion length as previously noted by reviewers, we did not go into detail as it was not the primary outcome of interest; we can certainly develop this further if the reviewer feels like it would be important.

5. In Figure S2, the measurements of both fimH carriage (panel A) and MDR (panel B) are binary in nature. So, what do the bars represent here? There should not be standard deviations in this type of data. Accompanying that, one would expect that a chi-square analysis rather than “an unpaired, nonparametric t-test” (line 279) would be appropriate for comparisons.

We removed the error bars and performed a chi-square test instead of a t-test. The figure has been updated to represent the edits.

6. The vertebrate animals information would benefit from additional clarification. The authors state that all the samples were collected for clinical purposes and prior to the start of any analysis (i.e., they were banked). What was the clinical indication for bladder biopsies in dogs with active UTIs?

Bladder biopsies are taken during uroscopy at the discretion of the treating veterinarian. The biopsies are sent to the pathology service to evaluate the bladder tissue for the presence of inflammation, infection, or neoplasm. These samples are banked and can be used for research purposes. We have tried to clarify this in the manuscript by adding that these are diagnostic specimens submitted for the management of a patient and not because of inclusion in the study. Still, all samples submitted to the NCSU CVM are approved to be used for research purposes.

Minor points:

1. Given that no methodologic details are not fully available in the abstract, one should remove the specific numerical data from the abstract. For example, “4.2 log10 CFU/well” doesn’t have specific meaning as the reader hasn’t yet read the methods.

This numerical data has been removed from the abstract per this reviewer’s request.

2. Line 44: “20% of dogs with UTIs will develop a recurrent infection…” during what period of follow-up (in reference 4)?

This has been updated.

3. Line 182: Beyond “similar morphology,” can the authors be more specific as to the size of intracellular communities or the apparent number of bacteria, in canine uroepithelial cells vs the immortalized human bladder cell line?

Not at this stage but this would be a good idea for future work.

4. Some of the figure legends could be shortened by reducing methodologic detail (these details already appear in the Methods section).

We have followed this suggestion and reduced the figure legends.

5. The Discussion could probably also be shortened by reducing the degree to which the current Results are recapped.

We have followed this advice and removed paragraph 1 that recapped the results.

6. Panels A and B of Figure 2 could probably be eliminated, as they reflect simple percentages that are described in the text.

We prefer to leave panels A and B in Figure 2 in but will leave this to the discretion of the editor.

Reviewer #2: The authors have written an informative manuscript. However, the authors should proofread the manuscript and correct some minor spelling mistakes and strictly follow the journal's author guidelines.

The sentence in line 22 and 23 is incomplete.

Corrected.

The sentence in line 63 and 64 is not well understood.

We have edited this sentence.

Please check the spelling of ' their) in line 66.

Corrected.

Please elaborate more on the statistical analysis. Most of the statement have been made in the 'results' section. For example, non-parametric t-test.

We have added to the statistical analysis section with statements from our figures/results sections.

The authors did not include control strains in the experimental section.

We did not use any control strains only clinical UPEC strains from canine UTIs as appropriate per the study design.

Authors should please take a second look at the clarity of the labels.

We have double checked the labels and corrected where appropriate. Most of this clarification was addressed through other reviewer comments.

Authors should make comments on the ethical approval.

We have added further comments on the ethical approval.
